# Circular Engineered Sortase for Interrogating Histone H3 in Chromatin

**DOI:** 10.1021/jacs.4c12585

**Published:** 2024-11-25

**Authors:** Samuel D. Whedon, Kwangwoon Lee, Zhipeng A. Wang, Emily Zahn, Congcong Lu, Maheeshi Yapa Abeywardana, Louise Fairall, Eunju Nam, Sarah DuBois-Coyne, Pablo De Ioannes, Xinlei Sheng, Adelina Andrei, Emily Lundberg, Jennifer Jiang, Karim-Jean Armache, Yingming Zhao, John W. R. Schwabe, Mingxuan Wu, Benjamin A. Garcia, Philip A. Cole

**Affiliations:** † Division of Genetics, Department of Medicine, Brigham and Women’s Hospital, Department of Biological Chemistry and Molecular Pharmacology, Harvard Medical School, Boston, Massachusetts 02115, United States; ‡ Department of Biochemistry and Molecular Biophysics, Washington University School of Medicine, St. Louis, Missouri 63110, United States; § Epigenetics Institute, Department of Biochemistry and Biophysics, Perelman School of Medicine, University of Pennsylvania, Philadelphia, Pennsylvania 19104, United States; ∥ Leicester Institute of Structural and Chemical Biology, Department of Molecular and Cell Biology, University of Leicester, Leicester LE1 7RH, U.K.; ⊥ Department of Biochemistry and Molecular Pharmacology, New York University Grossman School of Medicine, New York, New York 10016, United States; # Ben May Department for Cancer Research, The University of Chicago, Chicago, Illinois 60637, United States

## Abstract

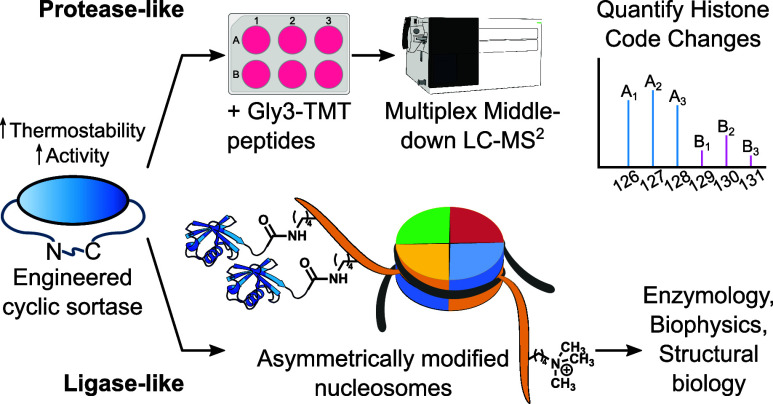

Reversible modification of the histone H3 N-terminal tail is critical in regulating the chromatin structure, gene expression, and cell states, while its dysregulation contributes to disease pathogenesis. Understanding the crosstalk between H3 tail modifications in nucleosomes constitutes a central challenge in epigenetics. Here, we describe an engineered sortase transpeptidase, cW11, that displays highly favorable properties for introducing scarless H3 tails onto nucleosomes. This approach significantly accelerates the production of both symmetrically and asymmetrically modified nucleosomes. We demonstrate the utility of asymmetrically modified nucleosomes produced in this way in dissecting the impact of multiple modifications on eraser enzyme processing and molecular recognition by a reader protein. Moreover, we show that cW11 sortase is very effective at cutting and tagging histone H3 tails from endogenous histones, facilitating multiplex “cut-and-paste” middle-down proteomics with tandem mass tags. This cut-and-paste proteomics approach permits the quantitative analysis of histone H3 modification crosstalk after treatment with different histone deacetylase inhibitors. We propose that these chemoenzymatic tail isolation and modification strategies made possible with cW11 sortase will broadly power epigenetic discovery and therapeutic development.

## Introduction

Understanding the patterns and functional interactions of histone tail post-translational modifications (PTMs) has emerged as a central challenge in epigenetics.^1^ The building blocks of cellular chromatin are nucleosomes, which comprise a histone octamer (four pairs of histones H2A, H2B, H3, and H4) wrapped by 147 bp DNA. Conformational changes in chromatin contribute to the regulation of cell growth, differentiation, and gene expression.^2,3^ Such chromatin structural changes are influenced by reversible histone modifications, which are inscribed by “writer” enzymes, removed by “eraser” enzymes, and functionally interpreted by “reader” domain proteins. Histone H3 N-terminal modifications are the focus of particular attention due to their central importance in gene regulation. Histone H3 N-terminal tail modifications include well-established Lys acetylation, methylation, and ubiquitination and lesser studied acylation modifications like propionylation, butyrylation, crotonylation, and succinylation.^4,5^ These and other PTMs are found in various combinations on histone H3 tails. This complex pattern of modifications affects chromatin structure and the writers, erasers, and readers that act on histones, but detailed molecular insights into how histone PTM crosstalk modulates these processes are generally lacking.^6,7^

In this study, we address two significant challenges in the field: limitations on the ready availability of designer nucleosomes and middle-down mass spectrometric analysis of histone H3 modifications. Although progress has been made over the past 15 years in the ability to prepare nucleosomes containing site-specific modifications on the H3 tail, this is still an onerous multistep task. This workflow requires individual expression and purification of all four histone proteins, including a truncated form of the modified histone, semisynthesis of the modified histone, octamer refolding, DNA isolation, and finally nucleosome reconstitution.^8,9^ Starting from scratch, this process takes about a month even in laboratories with experience. Moreover, substantial additional labor is required to produce nucleosomes containing distinct histone H3 tails, “asymmetric nucleosomes”, that can be employed to distinguish between biochemical effects mediated by modifications of H3 that occur on the same tails versus different tails.^10−13^

Middle-down mass spectrometric analysis of histone H3 can provide precise information about the interplay between modifications within individual histone H3 tails by evaluating intact H3 protein tails isolated from cellular histones.^14^ Current middle-down methods require purification of cellular histone H3 prior to treatment with the protease GluC.^15−17^ The resultant aa1–51 H3 peptide tails typically require complex and specialized chromatography for separation and characterization by tandem mass spectrometry, which is particularly challenging for such large peptide segments. Furthermore, this, like other middle-down proteomics approaches, lacks the quantitative strength of current tandem mass tag (TMT) isotopic labels, which are widely used in bottom-up mass spectrometry.^18^

**Figure 1:**
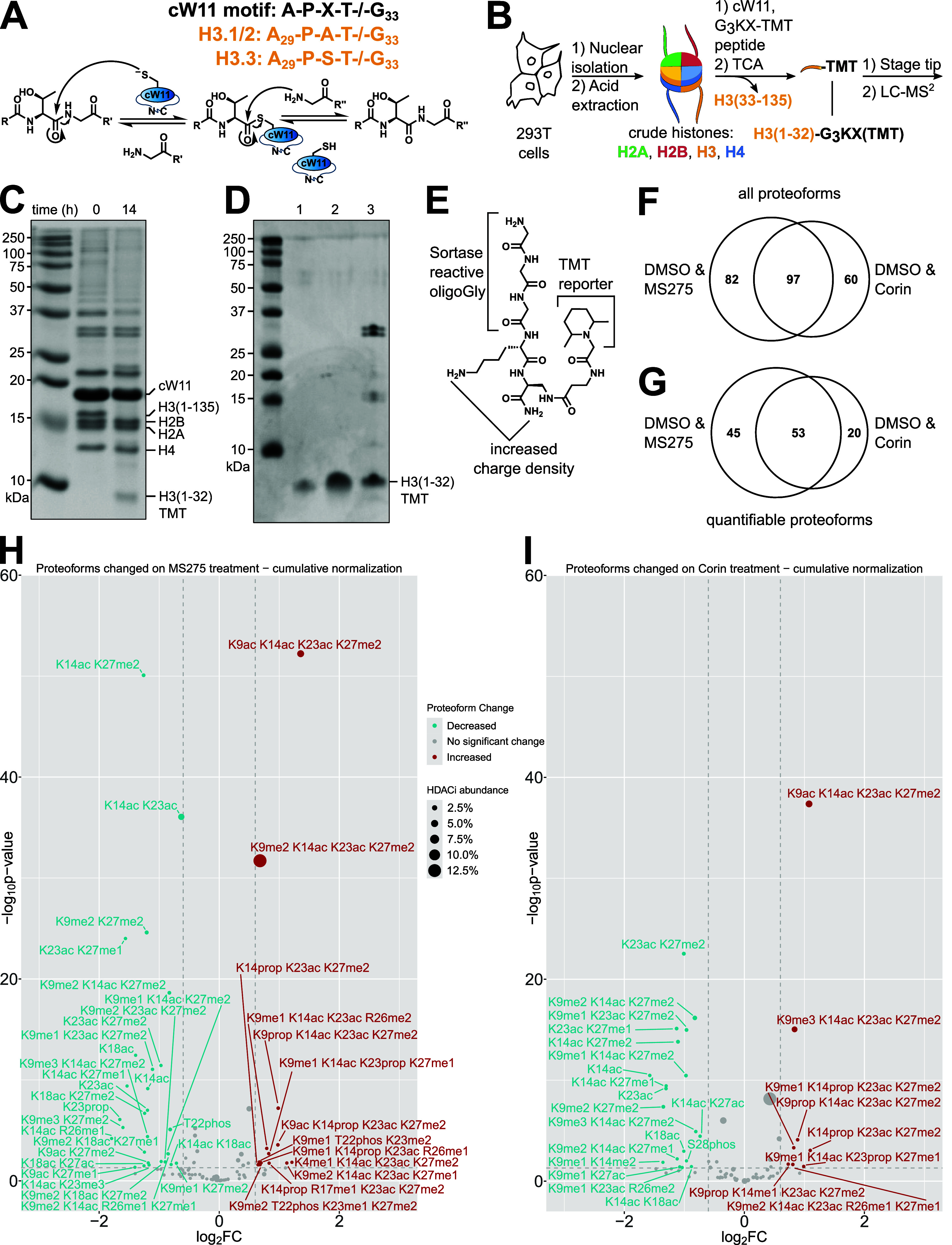
“Cut-and-paste” isolation of histone H3 tail peptides with cW11 sortase enables quantitative middle-down proteomics. (A) cW11 sortase recognition motif in H3 and transpeptidation mechanism. (B) Workflow for isolating tandem mass-tagged histone H3 tails with cW11 sortase. (C) SDS-PAGE of 14 h sortase reaction in a nuclear acid extract. (D) Tris-tricine PAGE of histone H3(1−34) synthetic standards (lane 1: 140 ng; lane 2: 420 ng) and TCA-precipitated H3(1−32)-TMT from the acid extract reaction (lane 3); bands between 25 and 37 kDa are histone H1. (E) Structure of the TMT-labeled oligoglycine peptide illustrating sortase reactive GGG, charge carrying K and carboxamide, and TMT-labeled aminoalanine. (F) Unique proteoforms detected in each 6plex sample and shared between the two samples. (G) Quantifiable proteoforms detected in each 6plex sample and shared between the two samples. Volcano plot of significant (*p* < 0.05) log 2-fold change and overall abundance of H3 proteoforms in HEK293T cells following treatment with (H) pan class I HDAC inhibitor MS275 or (I) LSD1/HDAC1/CoREST complex-specific inhibitor Corin.

Here, we address these limitations in histone H3 analysis through the application of a novel engineered sortase transpeptidase, cW11. The development of cW11 builds on the earlier work of Schwarzer and colleagues, who reported F40 sortase as a tool for histone H3 semisynthesis.^19^ F40 sortase was developed as a chemoenzymatic tool to catalyze H3 tail attachment to tailless histone H3 tail recombinant protein, which contains the sequence APXTG (aa29–33) rather than the natural sortase recognition epitope LPXTG (Figure 1a).^19,20^ While useful, F40 sortase shows relatively slow rates of H3 tail attachment with a standard amide bond sequence and relatively low yields of semisynthetic histone H3s. Sortase cW11 has been found to be a more effective catalyst of histone H3 transpeptidation reactions, facilitating H3 tail ligation to prefabricated tailless nucleosomes. Moreover, cW11 sortase can be employed to create asymmetric nucleosomes bearing distinct patterns of modifications on the H3 tails. The availability of such asymmetric nucleosomes has enabled new insights into molecular recognition of nucleosomes by reader, writer, and eraser proteins.^7,17,21^ In addition, the efficient isolation of H3 peptide tails from crude extracts of endogenous histones with cW11 permits concurrent labeling with TMTs. This “cut-and-paste” approach enhances the middle-down proteomics analysis of histone H3 tails.

## Results

### Development of cW11 Sortase

To improve on F40 sortase, mutant sortases containing amino acid replacements were designed based on structural considerations and previous studies on the development of enhanced sortase A (eSrtA).^20^ These mutations were introduced individually and then in combination, and mutant sortases were screened for transpeptidation-based cleavage of histone H3 in the presence of excess oligoglycine peptide. First-pass screening employed purified histone H3 with no modifications (Figure S1). D165A, the single most activating mutation identified in eSrtA, falls within the helix mutagenized in F40 and generally hinders the cleavage reaction. Combined mutations of D160, K190, and K196 synergistically enhanced activity without disrupting the selectivity imparted by F40 mutations (T164 and V168-Q172). The best-performing enzyme was tested against synthetic histone substrates with single modifications, and no significant bias was observed in the cleavage reaction (Figure S2).

With a sequence-selective sortase, sample handling steps in the typical middle-down proteomic workflow can be reduced (Figure 1b). Conducting the sortase reaction in a crude nuclear extract eliminates histone purification as a prerequisite for proteolytic isolation of the histone tail (Figure 1c and Figure S3). Sortase performs optimally at neutral pH and low salt concentrations; however, this compromises the solubility of endogenous histones. Sortase exhibits minimal tolerance for detergents, cosolvents, and chaotropes, which we sought to mitigate (Figure S4).^22^ Individual thermostabilizing mutations were identified using the FireProt web server and screened in combination (Figure S4).^23^ Sortase backbone cyclization was previously reported to enhance stability and was also incorporated, leading to enzyme cW11.^22,23^ Sortase cW11 exhibits superior activity in nuclear acid extracts at physiological temperatures. The cleaved histone tail peptides are readily separated from sortase and other components of the nuclear acid extract reaction by trichloroacetic acid precipitation (Figure 1d and Figure S5). Following supernatant buffer exchange and cleanup with C18 stage tips, the peptides are suitable for LC–MS/MS analysis.

### “Cut-and-Paste” Middle-Down Proteomics of Histone H3 Modifications

In the sortase transpeptidation, an N-terminal Gly is required for reaction with the enzyme-thioester intermediate, while C-terminal diversity is tolerated. By solid-phase peptide synthesis, a GGGKX peptide was prepared with β-aminoalanine at the C-terminal position. After orthogonal deprotection of the side chain β-amino group, the peptide resin was split into 6 portions, each of which was reacted with a unique TMT 6-plex NHS-ester reagent (Figure 1e). This peptide tag 6-plex system allowed concurrent analysis of histone tails from two cell treatment conditions, each in biological triplicate.

To explore cW11 sortase-mediated mass spectrometry analysis of H3 tails in a pharmacological application, we utilized this approach to evaluate the changes in the patterns of tail modifications in response to two different histone deacetylase (HDAC) inhibitors: MS275 and Corin.^24^ MS275 is a class I selective HDAC inhibitor, targeting HDACs 1–3, whereas Corin is a bivalent inhibitor that contains HDAC as well as an LSD1 demethylase targeting warhead. In this way, Corin appears to show selectivity toward CoREST-containing HDAC complexes. Treatment of HEK293T cells with these HDAC inhibitors or DMSO was performed for 6 h, after which nuclear acid extracts were isolated. Samples from these experiments were divided into separate bottom-up and middle-down proteomic analyses. Histone H3 tails isolated for the middle down were tandem mass tagged by cW11 sortase, with each replicate assigned a unique TMT. Quantification of individual H3 PTMs by GluC-based middle-down and bottom-up proteomics typically results in a Pearson correlation between 0.3 and 0.6.^26^ In this case, middle-down analysis of the sortase-derived H3 peptides resulted in a stronger correlation with bottom-up analysis of the same acid-extracted histone samples (Pearson correlations of ∼0.8) (Table S1).

By LC–MS/MS, we identified ∼150 uniquely modified peptides (proteoforms) in a combined DMSO vehicle/Corin sample and ∼180 in a combined DMSO vehicle/MS275 sample (Figure 1f and Tables S2 and S3). Nearly half of the proteoforms identified had sufficient TMT ion signals for quantitation (≥2 per sample, Figure 1g and Tables S4 and S5). This quantifiable fraction accounted for ∼45–50% of all signal intensity attributable to H3 tail peptides. Relative to the DMSO vehicle, both HDAC inhibitors significantly decreased H3 tail proteoforms with one or two modifications while increasing those with three or four modifications (Figure 1h,i). The most significant increase was seen in H3K9acK14acK23acK27me2; however, a greater increase was seen with MS275 than with Corin (155% vs 110% increase). It was hypothesized that H3K4me1/2 proteoforms would increase following Corin treatment due to LSD1 inhibition by Corin; however, H3K4 modifications were detected infrequently in this analysis, consistent with prior middle-down analyses.^27^ H3K4me1K14acK23acK27me2 was the sole H3K4me proteoform to exhibit a significant change, an increase following MS275 treatment. This increase is in line with recent work pointing toward a general stimulatory effect of H3 acetylation on the MLL family K4 methyltransferases.^17^

We also noted that HDAC inhibition led to accumulation of H3 tail propionylation (2–4%, Table S1). The recently reported acetyl/methyl PTM has an identical mass and is a theoretical alternative to our propionyl assignment.^28^ However, the two modifications differ in their diagnostic immonium ions, which contributed to our PTM scoring.^28^ Generally, the abundance of acetyl-methyl-Lys, as reported by Simon et al., was less <1% and predominantly on histone H4. Moreover, high-quality anti-propionyl antibodies have revealed the abundance of H3 propionylation, particularly at sites like K23, where we observed the greatest accumulation.^29^

### Streamlined Production of Modified Nucleosomes

Histone octamers are typically prepared by the individual purification of each core histone protein, followed by in vitro octamer assembly. Coexpression of the four core *Xenopus* histones in *E. coli* has been used to directly furnish octamer.^30^ In the course of this work, we attempted coexpression of the core histones with tailless histone H3 (aa33–135) and found that, in our hands, deletion of the H3 tail facilitated the isolation of intact histone octamer about 5-fold over comparable full-length H3 octamer (5 ± 2.5 mg/L culture) (Figure S6). This octamer could be readily wrapped by DNA (147 or 185 bp) to produce H3 tailless nucleosomes. These findings led us to pursue late-stage tail attachment to tailless nucleosomes by cW11 sortase. This would enable rapid designer nucleosome production from a common late-stage precursor and minimize the consumption of the synthetic H3 tail peptide. Enhanced sortase variants eSrtA and 5 M have been used to ligate histone H3 tail to nucleosomes and endogenous chromatin, respectively, but require an A29L mutation that complicates recognition of the heavily modified H3K27 site.^31,32^ F40 sortase is ineffective in this setting.

**Figure 2:**
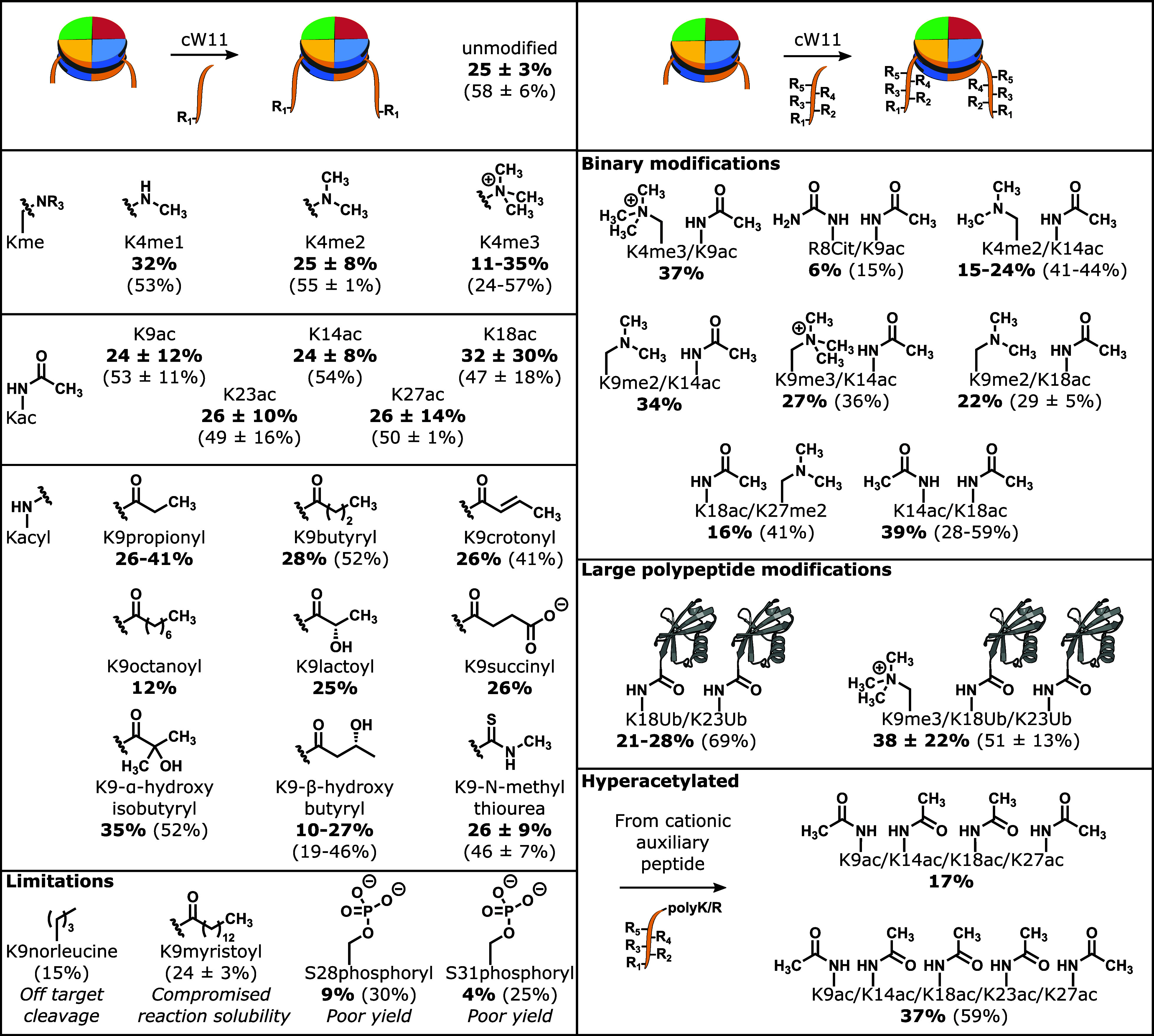
Designer nucleosome synthesis by sortase ligation. Single, combinatorial, and asymmetric modifications prepared by sortase nucleosome ligation (ubiquitin structure PDBID: 1UBQ).^25^ Isolated yields (bold) and yields estimated by area under the curve (parenthetical) are reported as a value (*n* = 1), range (*n* = 2), or mean with standard deviation (*n* ≥ 3).

We investigated the activity of cW11 sortase in ligating H3 tails to tailless nucleosomes (aa33–135), and Western blot revealed reasonable efficiency (∼90% ligation of H3) (Figure S7). Analytical anion exchange chromatography was used to precisely delineate the distribution of products, resolving nucleosomes with zero, one, or two H3 tails ligated (Figure S8). This revealed a similar ligation efficiency with respect to histone H3 (79 ± 13%), with optimized conditions converting all tailless nucleosomes to products with either one or two H3 tails ligated. Nucleosomes with two ligated H3 tails typically accounted for 58 ± 6% of products (25 ± 3% isolated yield) (Figure 2). At our standard nanomole scale, 100 μg of nucleosome is obtained for every 100 μg of peptide, which is ∼100-fold less peptide than required to make the same amount of nucleosome by sortase semisynthesis of histone protein.^33^

Late-stage H3 functionalization by cW11 ligation was evaluated for compatibility with modifications of lysine (acylations, alkylations, and ubiquitination), arginine (citrullination), and serine/threonine (phosphorylation) (Figure 2 and Figures S9 and S10). Ligation of alkylated peptides resulted in yields similar to those of an unmodified peptide (LC: 53 ± 11%; isolated: 26 ± 9%); however, modifications decreasing the peptide positive charge were observed to decrease yield. For single acylations, this could be overcome by substituting the Thr32 Gly33 amide linkage for a depsipeptide ester linkage.^19^ The first step in sortase transpeptidation, cleavage of the T-G amide bond, liberates a glycine dipeptide that competes with H3 in the transpeptidation reaction; however, the alcohol-terminated byproduct of ester cleavage is incompatible with the reverse reaction. Irreversible cleavage of the synthetic peptide C-terminus proved invaluable in the synthesis of multiply acetylated nucleosomes, where the charge masking effect of the acetylations was offset by the addition of multiple cationic residues after G34. Introduction of this cleavable C-terminal cationic auxiliary enabled ligation of peptides with five concurrent acetylations spanning all the major acetylation sites (K9, K14, K18, K23, and K27) (Figure 2 and Figure S11).

Though broadly compatible with histone PTMs, there are limitations to cW11 ligation. Introduction of a negative charge near the sorting motif (Ser28, Ser31) hinders ligation progress, resulting in isolable yields of 5–10%. Phosphorylation of Ser28 was found to have no effect on H3 tail cleavage during cW11 development (Figure S2). Thus, we hypothesize that the proximity of Gly33 to the DNA backbone (∼10–12 Å, PDBID: A1OI) leads to repulsion between the DNA backbone and proximal histone tail peptide phosphorylation.^31,34^

**Figure 3:**
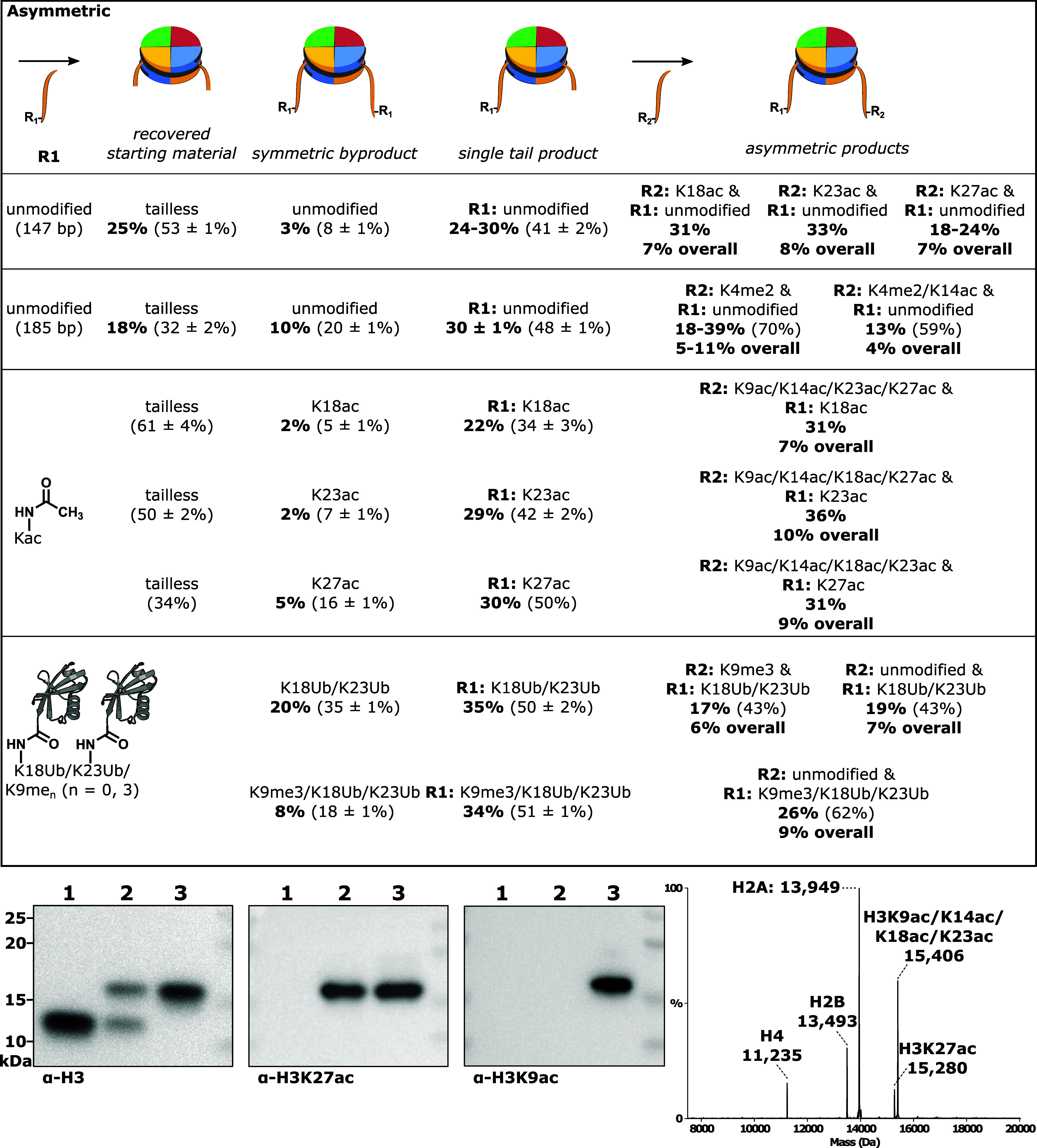
Asymmetric nucleosome synthesis by sortase ligation. (Top) Asymmetric modifications prepared by sortase nucleosome ligation. Isolated yields (bold) and yields estimated by area under the curve (parenthetical) are reported as a value (*n* = 1), range (*n* = 2), or mean with standard deviation (*n* ≥ 3). (Bottom left) Western blot characterization of 147 bp nucleosomes: (1) H3 (aa33–135) starting material; (2) asymmetric H3K27ac and H3 (aa33–135) intermediate; and (3) asymmetric H3K27ac and H3K9ac/K14ac/K18ac/K23ac product. (Bottom right) Deconvoluted mass spectrum of 147 bp asymmetric H3K27ac and H3K9ac/K14ac/K18ac/K23ac nucleosome.

Anion exchange chromatography resolves nucleosome ligation products from free DNA and also separates nucleosomes with zero, one, or two copies of H3(aa33–135). Isolation of nucleosomes with one copy of full-length H3 and one copy of H3(33–135), followed by a second round of sortase ligation, allows production of nucleosomes with asymmetric modifications of the H3 tail (Figure 3 and Figure S12). Asymmetric, or heterotypic, nucleosomes are sought after tools for unraveling crosstalk between modifications of the histone tails, but current strategies for their production are cumbersome.^11^ The cW11 ligation simplifies their production, requiring only minor changes in peptide stoichiometry to favor the production of the intermediate single-tail nucleosome and subsequent asymmetric nucleosome.

### Confirmation of Nucleosome Biochemical Integrity

We have previously reported site-specific nucleosome deacetylation rates for multiple enzymes and complexes at most of the major acetylation sites on histone H3.^33,35,36^ In theory, our new approach to preparing nucleosomes by late-stage tail addition could lead to unintended consequences in biochemical analysis. For example, residual peptide could be present, which might compete with nucleosome as an enzyme substrate and alter nucleosome recognition by masking DNA, either of which was predicted to affect the deacetylase rate. As a quality control for the cW11 ligation, we conducted parallel deacetylation rate studies using nucleosome substrates prepared by our new approach and using previously established methods. Using those HDACs most extensively characterized in our lab, we found no significant difference in deacetylation rates (Figures S13 and S14).^36,37^ Finally, cryo-EM was used to validate nucleosome structure and integrity and resulted in a structure consistent with numerous other 147 bp nucleosome structures (Figure S15).

### Revealing Histone Deacylase Activity with Acylated Nucleosomes

Reports of histone lysine succinylation, propionylation, and butyrylation, among others, have steadily accumulated in recent years, while characterization of their erasers has lagged. Broad functional group compatibility in the sortase ligation enabled the characterization of histone deacylase activity toward these variant acylations. Drawing upon previously reported histone acylation selectivity and proposed modes of nucleosome recognition, we selected the nuclear or nucleocytoplasmic HDACs, mitosis-associated HDAC complex (MiDAC) and LSD1/HDAC/CoREST (LHC) complex, as representatives of class I HDAC complexes, and sirtuins 1, 2, and 6 as representatives of class III HDACs with which to explore the class- or complex-specific modes of deacylation.^33,36,38,39^

**Figure 4:**
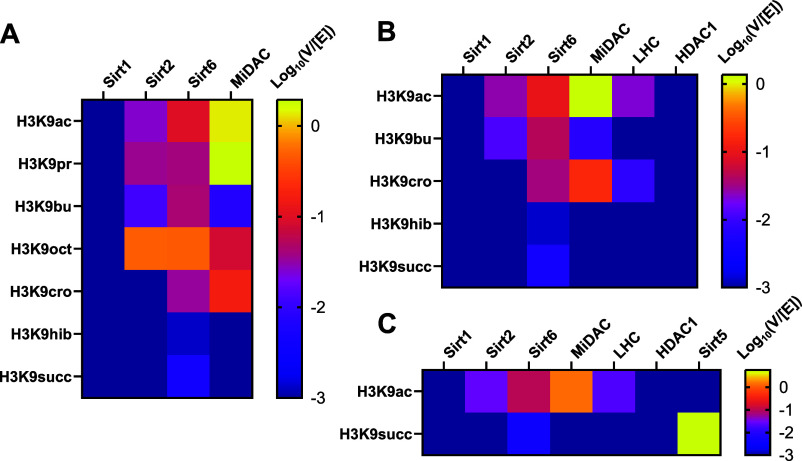
Trends in histone deacylase activity toward metabolic acylations. (A) Log 10 transformed *V*/[*E*] (min^–1^) of sirtuins 1, 2, and 6 and MiDAC toward 147 bp nucleosomes with one to eight carbon acylations of H3K9. (B) Log 10 transformed *V*/[*E*] (min^–1^) of class I and class III HDACs toward 147 bp nucleosomes with four carbon metabolism-linked acylations of H3K9. (C) Log 10 transformed *V*/[*E*] (min^–1^) of class I and class III HDACs toward 147 bp nucleosomes with H3K9 succinylation.

Across all sites on the H3 tail, K9 is generally the most rapidly deacetylated and was therefore selected to probe reactivity trends. Removal of acetyl, propionyl, butyryl, and octanoyl modifications were compared (Figure 4a and Figures S16–S19), while myristoylation proved intractable in the sortase nucleosome ligation (Figure 2). Sirtuins 2 and 6 were superior long-chain deacylases processing K9octanoyl substrates 18-fold faster than K9ac and ∼5-fold faster than K9ac, respectively. Sirtuin 1 exhibited no activity toward any acylation, consistent with prior observations. Long-chain deacylation by the class I HDACs is not much evidenced, so MiDAC was selected for comparison as its baseline deacetylation of K9 is the fastest measured by an order of magnitude. Deacylation by MiDAC slowed with chain length, decreasing ∼180-fold (K9butyryl) and ∼16-fold (K9octanoyl) relative to that of K9ac. That octanoylation was processed at all is chemically interesting, and particularly that MiDAC favors it ∼10-fold over butyrylation (Figure 4a and Table S6).

For each deacylase enzyme with detectable activity, butyrylated nucleosomes were consistently the slow substrate, perhaps accounting for its observed metabolic accumulation in specific tissue types.^40^ However, sirtuin catalysis is reported to be enhanced by long-chain acylations that pack within the active site. Exploring the conformational contribution to substrate selectivity for deacylases, four carbon acylations with links to metabolic state were tested, including crotonylation, hydroxyisobutyrylation, and succinylation (Figure 4b and Figures S20–S25). Of these decrotonylation exhibited a rate similar to debutyrylation for Sirt6 and faster than debutyrylation for MiDAC and LHC (Table S7). Removal of the branched and charged modifications was unmeasurable for all but Sirt6, which was exceptionally slow. Succinylation is the preferred substrate of Sirt5, which desuccinylated nucleosomes at the fastest rate measured among all deacylases and acylations surveyed here (*V*/[*E*] = 5.7 min^–1^) (Figure 4c, Figure S26, and Table S8).

### Symmetric and Asymmetric Nucleosome Tools for Testing Consequences of Histone Hyperacetylation

Among the synthetic nucleosomes prepared were those with five acetylations of H3 at K9, K14, K18, K23, and K27, which are the predominant sites of H3 acetylation detected in the sortase middle-down data and reported by others. The sharp rise in multiply acetylated peptides following HDAC inhibition, and prior reports of histone acetyltransferase “acetyl spray” activity, prompted investigation of these hyperacetylated substrates.^41^ Extensive acetylation increases histone tail dynamic motion and alters the local electrostatic environment encountered by regulators like HDACs.^42−44^ Whether this influences the rate of site-specific deacylation was tested using a K9ac/K14ac/K18ac/K23ac/K27ac penta-acetylated substrate, and site-specific rates were compared to previously reported rates for each of K9ac, K14ac, K18ac, K23ac, and K27ac.^35,36,45^

**Figure 5:**
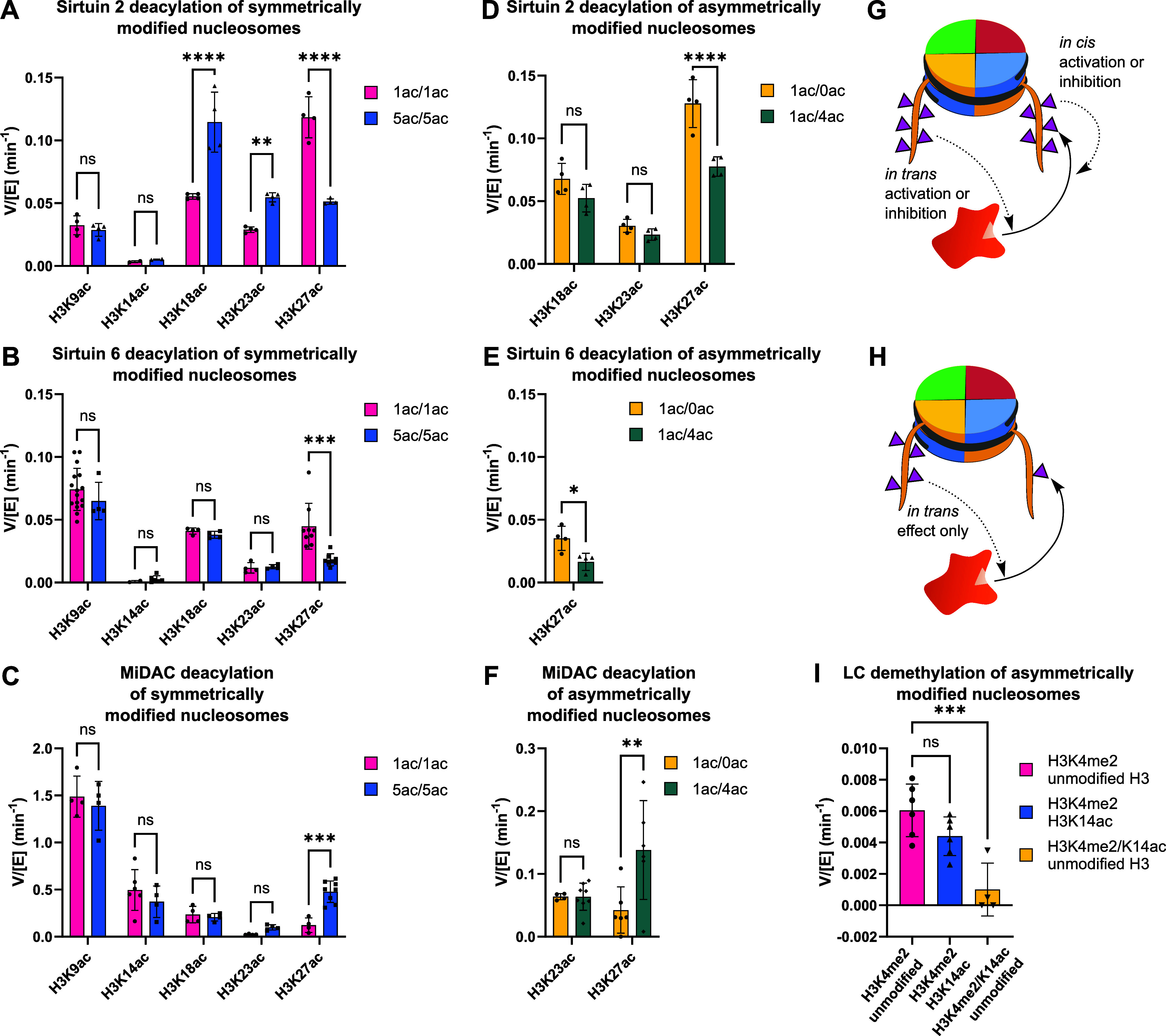
Symmetric and asymmetric effects of hyperacetylation on deacylase and demethylase activity. (A) Sirt2, (B) Sirt6, and (C) MiDAC site-specific deacetylation rates with symmetrical monoacetylated (1ac/1ac) and penta-acetylated (5ac/5ac) 147 bp nucleosomes. (D) Sirt2, (E) Sirt6, and (F) MiDAC site-specific deacetylation rates with asymmetric 147 bp nucleosomes isolating the specified acetylation site on a single tail; the second H3 tail was modified with either four acetylations (1ac/4ac) at the other predominant acetylation sites or zero acetylations (1ac/0ac). (G) Illustration of in cis and in trans PTM interactions with a regulatory enzyme acting on a nucleosome substrate. (H) Illustration of an asymmetric nucleosome used to test for an in trans effect on a PTM regulatory enzyme. (I) LSD1-CoREST1 (LC) demethylation rates with asymmetric nucleosomes containing H3K14ac concurrently on the same histone H3 with H3K4me2 (yellow), H3K4me2, and H3K14ac separately on different histone H3 (blue) and without H3K14ac (pink) (* indicates *p* < 0.05, ** indicates *p* < 0.01,*** indicates *p* < 0.001, and **** indicates *p* < 0.0001, all error bars indicate standard deviation).

The rate of deacetylation of penta-acetylated nucleosomes by different enzymes was monitored by Western blot with site-selective antiacetyl-Lys antibodies, the site-specificity of which was validated (Figure S27) with our designer nucleosomes (Figure 5a–c and Figures S28–S31). Penta-acetylation influenced removal for a select set of acetyl-Lys locations with distinct impacts among the different enzymes. Deacetylation of H3K27 by both Sirt2 and Sirt6 slowed ∼2-fold, while deacetylation by MiDAC increased nearly 4-fold. Sirt2 exhibited a striking change in site selectivity from K27 to K18, due to ∼2-fold deacetylation rate increases at K18 and K23. To try to better understand the mechanistic basis for these effects, we used the sequential ligations enabled by cW11 sortase to prepare asymmetrically modified nucleosome substrates.

For each site exhibiting a rate changed by penta-acetylation, an asymmetrically modified nucleosome was prepared to isolate that site from the other four acetylations. For example, all three enzymes surveyed exhibited altered rates of deacetylation at K27, and the nature of those rate changes could be assayed using nucleosomes with one copy of H3K27ac and one copy of H3K9ac/K14ac/K18ac/K23ac. Compared to the symmetrically acetylated substrates assayed previously, this substrate has half the effective concentration of K27ac, one copy per nucleosome (Figures S32 and S33). To control for this, rate comparisons were made by using asymmetric nucleosomes with one unmodified copy of H3 and one copy of H3K27ac (Figure S33). Deacetylation of asymmetric K9ac/K14ac/K18ac/K23ac and K27ac nucleosomes by Sirt2 and Sirt6 was ∼2-fold slower than the matched control (Figure 5d,e, Figures S34 and S35, and Tables S9 and S10). This is consistent with competition between acetylation sites driving the rate decrease, rather than a local acetylation slowing the rate through altered enzyme recognition. With the same substrates, MiDAC showed ∼3-fold faster deacetylation of the more acetylated asymmetric substrate than the singly acetylated asymmetric substrate (Figure 5f, Figure S36, and Table S11). This in trans stimulation (Figure 5g,h) suggests a processive mechanism at the level of the nucleosome rather than a single histone tail and suggests that histone tail mobility alone does not explain the accelerated deacetylation of K27 by MiDAC in a hyperacetylated context. Notably, MiDAC exists as a predominantly tetrameric complex with four catalytic HDAC1/2 modules, and the dimeric MiDAC subunit DNTTIP1 is a nucleosome acidic patch binder.^38,46^ We speculate that multivalent binding through these domains could drive crosstalk between H3 tails. At this stage, it is unclear why nearby H3K23ac is not subject to the same in trans stimulation, although it is possible that this could depend on the context of the neighboring amino acids, as has been previously observed with class I HDAC complexes.^35^

Deacetylation of K18 and K23 by Sirt2 was accelerated in the penta-acetylated context, prompting assays in which each site was isolated individually (Figure 5d). In each case, whether the opposite H3 tail had zero or four acetylations, the deacetylation rates were insignificantly different. Here, accelerated deacetylation depends on the in cis modification of one H3 tail with multiple acetylations.

### Symmetric and Asymmetric Nucleosomes as Tools for Unraveling Methylation-Acetylation Crosstalk

We have previously reported that demethylation of H3K4me1/2 by LSD1-CoREST1 (LC) or LHC slows >5-fold in the presence of H3K14ac specifically.^47^ Recent crystallographic evidence points at a candidate in cis mechanism but cannot rule out in trans inhibition.^37^ Final resolution of the inhibitory effect was attainable using a series of asymmetrically modified nucleosome substrates in which K4me2 and K14ac modifications could be sequentially isolated. Demethylation rates for nucleosomes with H3K4me2 and one of either unmodified H3 or H3K14ac were insignificantly different (Figure 5i and Figure S37). To control for absolute PTM concentration, these were both compared to asymmetric nucleosomes with H3K4me2/K14ac and unmodified H3, which again exhibited >5-fold reduction in demethylase activity by LC.

### DNMT1 Recognition of Symmetric versus Asymmetric Nucleosomes

**Figure 6:**
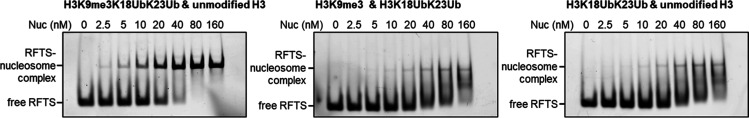
Combinatorial effect of H3K9me3 and K18Ub/K23Ub on DNMT1 RFTS domain binding. RFTS-sfGFP EMSA following titration with asymmetric H3K9me3/K18Ub/K23Ub and unmodified H3 nucleosome (left), asymmetric H3K18Ub/K23Ub and H3K9me3 nucleosome (middle), and asymmetric H3K18Ub/K23Ub and unmodified H3 nucleosome (right).

Multimonoubiquitination of histone H3 has been reported to direct binding by DNMT1, as has H3K9me3. The contribution of each modification has been partially evaluated using peptide, protein, and recently nucleosome substrates.^48−50^ The replication foci targeting sequence domain (RFTS) of DNMT1 has an atypical H3K9me3 binding site, but whether this exclusively reads methylation in cis was untestable in prior investigations. To address this, peptides, and subsequently asymmetric nucleosomes, were prepared with unmodified H3 and either K18Ub/K23Ub or K9me3/K18Ub/K23Ub, as well as H3K9me3 and K18Ub/K23Ub (Figures S38 and S39). An electrophoretic mobility shift assay (EMSA) was used to assess differences in the binding of each substrate with a fluorescent RFTS domain fusion construct. The combination of K9me3/K18Ub/K23Ub nucleosome and RFTS domain resulted in a clearly resolved band shift at single-digit nanomolar concentrations (Figure 6, left). The other two nucleosomes produced diffuse and smeared shifts of the RFTS band and consistently showed a weak signal for the complex band (Figure 6, center, right). This was observed with either component (RFTS or nucleosome) used as titrant and appeared constant over time (Figures S40 and S41). Based on this EMSA behavior, we deduce that the in cis K9me3/K18Ub/K23Ub modifications confer substantially enhanced stability of the RFTS-nucleosome interaction, which are not maintained when K9me3 is present in trans.

## Discussion

Here, we identify a powerful transpeptidase, cW11 sortase, that can efficiently ligate and cleave N-terminal H3 tails from histone H3. This has facilitated three important methods in chromatin analysis. First, cW11 sortase can be used to simply and rapidly produce site-specifically modified nucleosomes by permitting late-stage attachment of H3 tails to H3 tailless nucleosomes. An unexpected dividend that accelerates this approach is that the H3 tailless histone octamer can be efficiently generated by coexpressing tailless H3 with the other three core histones. This not only reduces the time needed to generate designer nucleosomes from about one month to 1 week but also dramatically reduces synthetic tail peptide amounts needed for ligation. We have shown that this cW11 sortase-driven strategy can easily incorporate a wide range of single and multiple PTMs (acetyl, acyl, and methyl) into nucleosomes. This is especially useful for ubiquitin-like modifications, which present a formidable synthetic challenge, particularly when introduced along with other PTMs.^51−53^ Streamlining such syntheses facilitates analysis of these nucleosomes in enzymatic and binding experiments.^50,54^ These studies have uncovered interesting new selectivities of deacylases that interconnect the metabolism and chromatin structure. Of particular note, both class I and class III HDACs remove acylations with linear chains up to eight carbons; however, branched or polar acylations are exclusively processed by specific sirtuins. Interestingly, ligation of H3 tails to DNA-free histone octamers using engineered sortase is not readily achievable due to the high salt concentration (2 M NaCl) needed for octamer stability.

Second, cW11 sortase provides an attractive and reliable route to asymmetric nucleosomes, in which the two different H3 tails contain distinct patterns of site-specific PTMs. Prior work on the construction of asymmetric nucleosomes with PTMs on histones H2A, H2B, and H3 has been reported but is technically demanding and not widely used.^11,12^ Our simple stepwise addition of H3 tails with a single chromatographic isolation of the intermediate single-tail form represents a relatively convenient alternative. Using this approach, we examined in asymmetric nucleosomes the crosstalk between H3K4me2 and H3K14ac by LSD1 demethylase, multiacetylated H3 tails by various histone deacetylases, and Lys ubiquitination and methylation by the RFTS reader domain of DNMT1. These experiments have revealed cases where specific patterns can impact eraser or reader interactions specifically in cis or trans depending on the site of modification and eraser/reader involved.^7,17^ While the precise mechanisms for these PTM crosstalk influences remain to be elucidated, these multifaceted regulatory features provide interesting and novel insights into histone mark crosstalk and highlight the necessity of studying asymmetric nucleosomes to understand the molecular recognition of individual chromatin interactors.

Third, we adopted cW11 sortase to perform a “cut-and-paste” method to isolate and analyze purified intact histone H3 tails from a human cell line. Though the position of the sortase recognition motif precludes quantification of H3K36 modifications, this procedure imparts quantitative power to the LC–MS/MS analysis of these tails through multiplex tandem mass tagging. With this approach, we were able to discern some overlapping but also distinct H3 tail PTM patterns after treatment with two types of HDAC inhibitors. We believe that this method can be generally useful for analyzing other epigenetic agents and specific cell states. The use of the chemoenzymatic labeling step with isotopic barcodes avoids the more complicated electrophilic chemical tandem mass tagging, which is avoided on large peptides because of complex reactivities. We propose that the use of engineered sortase tagging may be broadly useful in middle-down proteomics.

## Experimental Methods

### Sortase Expression and Purification

Plasmids for the relevant proteins and nucleic acids were transformed into *E. coli*, or cotransformed in the case of octamer, then selected with the relevant antibiotics, and grown from cell stocks. Sortase cW11 (from LOBSTR Rosetta(DE3) *E. coli*) was grown at 37 °C from an overnight starter culture of Luria–Bertani (LB) medium (Sigma) with ampicillin (100 mg/L), of which 10 mL was used to inoculate 1 L of the same medium used for overexpression. Cells were grown to an OD_600_ of 0.6–0.8, then induced with 0.5 mM IPTG for 3 h at 37 °C. Cells were harvested by centrifugation (4000*g*, 30 min, 4 °C), and the cell pellet was resuspended in 5 volumes of chilled (4 °C) lysis buffer (10 mM Tris, pH 7.5 at 25 °C, 0.1% Tween-20). Resuspended pellets were mixed on a Dounce homogenizer to uniformity and lysed by three passages through a microfluidizer. The supernatant was cleared by centrifugation (12,000*g*, 30 min, 4 °C), and the supernatant was passed over 5 mL of Ni NTA agarose twice by gravity. Resin was washed with 10 column volumes (CV) of lysis buffer, followed by 10 CV of wash buffer (10 mM Tris, pH 7.5 at 25 °C, 500 mM NaCl), then 5 CV of imidazole wash buffer (10 mM Tris, pH 7.5 at 25 °C, 0.1% Tween-20, 10 mM imidazole), and eluted with 5 CV of elution buffer (10 mM Tris, pH 7.5 at 25 °C, 400 mM imidazole). The elution was dialyzed three times (Spectra/Por 12–14 kDa MWCO membrane, Spectrum Laboratories, 50 mM Tris, pH 7.5 at 25 °C, 150 mM NaCl, 5 mM CaCl_2_) at 4 °C and then concentrated (Amicon, 10 kDa MWCO, 4 °C) to 2–6 mM with frequent sample mixing to prevent aggregation.

### Octamer Expression and Purification

Histone octamer (from LOBSTR Rosetta(DE3) *E. coli*) was grown from an overnight starter culture (30 °C) of LB medium (Sigma) with ampicillin (100 mg/L), kanamycin (50 mg/L), streptomycin (100 mg/L), and chloramphenicol (34 mg/L), which was not allowed to reach saturation. Growth for overexpression was initiated by adding 10 mL of starter culture to inoculate 1 L of the same medium. Octamer growth is typically slow, taking up to 12 h to reach the target induction OD_600_ of 0.8. Expression was induced by adding IPTG to 0.5 mM, and cultures were grown overnight (12–16 h) at 25 °C. Cells were harvested by centrifugation (4000*g*, 30 min, 4 °C), and the cell pellet was resuspended in 5 volumes of chilled (4 °C) lysis buffer (20 mM Tris, pH 7.5 at 25 °C, 2 M NaCl, 0.5 mM TCEP). Resuspended pellets were dounced to uniformity and lysed by three passages through a microfluidizer. Supernatant was cleared by centrifugation (12,000*g*, 30 min, 4 °C), and the supernatant was batch bound to 10 mL of Ni NTA agarose with gentle agitation at 4 °C. Resin was pelleted by centrifugation (500*g*, 5 min, 4 °C), and the supernatant was removed. Resin was washed with 5 CV lysis buffer, followed by 5 CV wash buffer (20 mM Tris, pH 7.5 at 25 °C, 2 M NaCl, 0.5 mM TCEP, 20 mM imidazole), and then eluted with 5 CV elution buffer (20 mM Tris, pH 7.5 at 25 °C, 2 M NaCl, 0.5 mM TCEP, 200 mM imidazole). The elution was concentrated (Amicon, 10 kDa MWCO, 4 °C) to 5–10 mg/mL by 280 nm absorbance (nanodrop) and incubated overnight with 0.01 mass equivalents of TEV protease at 4 °C. Octamer was further purified on an FPLC using a Superdex 200 column (20 mM Tris, pH 7.5 at 25 °C, 2 M NaCl, 0.5 mM TCEP), and then purified fractions were pooled and concentrated to 5–10 mg/mL. Glycerol and NaCl (5 M) were added to final concentrations of 15% and 2 M, respectively, and the samples were mixed thoroughly and aliquoted before flash freezing for storage at −80 °C.

### Mammalian Cell Growth, Nuclear Acid Extraction, and Histone Tail Isolation

HEK293T cells (ATCC) were grown in DMEM supplemented with 5% FBS and 1% pen/strep (37 °C, 5% CO2) and regularly tested by PCR for mycoplasma contamination. All experiments were conducted within 10 passages of stock thawing. For treatment with the DMSO vehicle, MS275 (LC Laboratories, cat. no. E-3866, Lot: ENT-102) or Corin (Med Chem Express, cat. no. HY-111048/CS0034060, Lot: 41808), a single 15 cm plate of HEK293T cells was grown to near confluence and split. Cells were washed with Dulbecco’s phosphate buffered saline (DPBS, D8537, Sigma-Aldrich), liberated with TryplE, and quenched with DMEM, and then cell count and viability (99%) were determined by trypan blue staining (Countess III). Plates (10 cm) were seeded in triplicate (4.4 × 10^6^ cells/plate) and recovered for 2 days in DMEM to ∼50% confluence. Following media change, stocks of drug were prepared in sterile filtered DMSO, then diluted in DMEM and added to plates to a final concentration of 10 μM MS275, or 2 μM Corin, and 1% DMSO. Cells were incubated for 6 h in the presence of vehicle or drug, after which the media was aspirated, cells were washed with DPBS, liberated with TryplE, and quenched with DMEM. Cells were pelleted, and the supernatant was removed by vacuum, then washed once with cold (4 °C) DPBS, pelleted again, and the supernatant was again removed by vacuum before flash freezing the pellets.

Pellets were thawed on ice and gently resuspended in 10 pellet volumes of nuclear isolation buffer (15 mM Tris, pH 7.5, at 25 °C, 60 mM KCl, 15 mM NaCl, 5 mM MgCl_2_, 1 mM CaCl_2_, 250 mM sucrose, 5 mM sodium butyrate, 0.3% NP-40, 1× Pierce protease inhibitor). After incubating for 5 min on ice, nuclei were pelleted (600*g*, 5 min, 4 °C), and the supernatant was removed. Nuclear pellets were washed twice by gentle pipetting with the same volume of detergent-free (no. NP-40) nuclear isolation buffer, pelleting, and removing the supernatant as before. To the pellet was added 5 initial pellet volumes of ice cold 0.4 N H_2_SO_4_, followed by vigorous mixing, then 2 h of constant agitation at 4 °C. Insoluble material was separated by centrifugation (3400*g*, 10 min, 4 °C), and the supernatant was collected. The supernatant was dialyzed against chilled 2.5% acetic acid and then twice against chilled 0.05% trifluoroacetic acid. The concentration of histone protein in the dialyzed supernatant was estimated by A280 (nanodrop), and the solution was then aliquoted, flash frozen, and freeze-dried.

Acid extracted protein was resuspended in reagent grade water to 100 μM histone H3. This was diluted into reaction buffer (final concentration 40 mM PIPES, pH 7.5, 1 mM DTT, 5 mM CaCl_2_) to a final H3 concentration of 20 μM. To this was added cW11 sortase to 100 μM and a single oligoglycine TMT peptide to 1 mM. The reactions were placed in a 37 °C incubator overnight (12–16 h), then moved onto ice and chilled for 10 min. Saturated trichloroacetic acid was slowly added to 5% of the reaction volume and placed back on ice for 5 min, then vigorously mixed by vortexing, and incubated for a further 10 min on ice. Insoluble material was pelleted by centrifugation (16,000*g*, 10 min, 4 °C), and the supernatant was recovered. The supernatant was buffer exchanged against ice-cold 0.05% trifluoroacetic acid in a pre-equilibrated spin concentrator (Amicon, 3 kDa MWCO, 14,000*g*, 10 min, 4 °C) for a total of 5 rounds. The buffer exchanged solution was recovered and freeze-dried. The sample was resuspended in reagent grade water, and one-third was taken for duplicate quantification against an amino acid analyzed standard by 16% Tris-tricine gel. Samples were again freeze-dried, then resuspended in 0.05% trifluoroacetic acid, cleaned up using stage tips packed in house, and concentrated by SpeedVac.

### Middle-Down Proteomics

Histone tails were resuspended in 0.1% formic acid and mixed to a 1:1:1:1:1 mass ratio based on gel quantification. Mixtures of DMSO-treated samples (TMT-126, 127, and 128) and samples treated with either MS275 or Corin (TMT-129, 130, and 131) were prepared to a concentration of 10 ng/μL and mixed thoroughly. 20 ng was injected for each analysis. Samples were analyzed on a Vanquish Neo LC instrument (Thermo Fisher Scientific) coupled to an Orbitrap Ascend Tribrid mass spectrometer equipped with ETD, PTCR, and UVPD (Thermo Fisher Scientific). The LC was operated in trap-and-elute mode using a PepMap Neo C18, 100 Å, 5 μm, 0.3 mm × 5 mm Trap Cartridge (Thermo Fisher Scientific) and an Easy-Spray PepMap Neo C18, 100 Å, 2 μm, 75 μm × 150 mm column (Thermo Fisher Scientific) heated to 40 °C. The flow rate was 0.3 μL/min. Solvent A was 0.1% formic acid, and Solvent B was 0.1% formic acid in acetonitrile. The LC gradient consisted of 2 to 15% Solvent B in 40 min, followed by 15 to 25% Solvent B in 6 min. The mass spectrometer was operated in data-dependent acquisition mode with a 2 s cycle time. MS1 spectra were collected in the Orbitrap with a resolution of 60k and a scan range of 375–600 *m*/*z*. Charge state and precursor selection range filters were applied to select peptides with a charge state of 8 from 480 to 540 *m*/*z* for MS2. Dynamic exclusion was enabled, and precursors were excluded for 9 s after being selected 1 time. MS2 spectra were collected with an isolation window of 1.6 *m*/*z*, a resolution of 30,000, and 2 microscans. EThcD was performed with an ETD reaction time of 20 ms, an ETD reagent target of 1 × 10^5^, a max ETD reagent injection time of 20 ms, a supplemental activation collision energy of 15%, and a normalized AGC target of 200%.

Raw data (10.7910/DVN/3IOGQ8) was analyzed using Byonic MS/MS search engine (Protein Metrics) using the following settings:

No protease cleavage; 500 ppm precursor tolerance; 15 ppm fragment tolerance; maximum 5 precursors; fragmentation type EThcD; maximum 6 common PTMs; Common PTMs Methyl/+14.015650@K, R, max 3|Dimethyl/+28.031300@K, R, max 3|Trimethyl/+42.046950@K, max 3|Phospho/+79.966331@S, T, max 2|Acetyl/+42.010565@K, max 5; maximum 1 rare PTM; Propionyl/+56.026215@K|rare1; fixed modification of H with TMT 6-plex; TMT6plex/+229.162932@H; fixed modification of protein C-terminus with 76.1001 Da mass (accounting for the mass difference between H and [K + 2,3-diaminopropionamide]).

Data was searched against a FASTA database of histone protein sequences, common contaminants, and the following sequences for H3.1 and H3.3 peptides:

H3.1 ARTKQTARKSTGGKAPRKQLATKAARKSAPATGGGH

H3.3 ARTKQTARKSTGGKAPRKQLATKAARKSAPSTGGGH

Peptide spectrum matches (PSMs) from Byonic were exported and subsequently processed in R (4.3.2) using RStudio, custom scripts (available at 10.7910/DVN/3IOGQ8), and packages parallel (4.3.2), stats (4.3.2), purrr (1.0.2), mzR (2.36.0), tidyr (1.3.1), stringr (1.5.1), dplyr (1.1.4), xml2 (1.3.6), readr (2.1.5), readxl (1.4.3), writexl (1.5.0), tidyverse (2.0.0), RColorBrewer (1.1–3), and ggrepel (0.9.5). Briefly, PSMs were filtered for high confidence matches with a posterior error probability ≤0.05 and a Byonic delta mod score ≥10 (a measure of confidence in PTM sequence location). Spectra were further subdivided by the number of PSMs reported in Byonic, and then EThcD ions (a, b, c, y, z, and neutral loss; 15 ppm error tolerance) consistent with the PSM(s) were identified within the spectrum. Fragment ions with at least one heavy isotope peak (e.g., 13C) were treated as high confidence and used to determine proteoform abundance in the sample as a whole. This criterion was not applied to TMT reporter ions, the isotopic impurities of which were processed separately. Proteoform abundance was calculated as the fraction of annotated fragment ion intensity that can be attributed to a single proteoform relative to the sum of annotated fragment ion intensity for all proteoforms. For spectra assigned to multiple PSMs, the intensity from shared fragment ions was divided between the relevant PSMs. Reporter ion signal intensities were channel normalized to the maximum average channel (e.g., TMT-126) signal intensity, averaging over all spectra with a PSM. Mean and median channel normalization produced broadly similar results (Tables S2–S5). Channel normalized reporter ion signal intensities were log 2 transformed for statistical analysis.

Spectra/scans with ≥2 reporter ions per treatment condition were used in quantification, and all such scans attributable to a single proteoform (e.g., H3K14ac/K23ac/K27me2) were grouped. For each treatment condition (drug or vehicle), all log 2 transformed reporter ion signals from a proteoform grouping were averaged, and the difference between treatment condition averages is reported as the log 2-fold change in proteoform abundance. Statistical significance was assessed by two-sided *t*-test (*p* < 0.05) using R stats (4.3.2).

### Bottom-Up Proteomics

Nuclear acid extracts were resuspended in propionylation buffer (50 mM NH_4_ HCO_3_, pH 8.0) to 1 μg/μL and mixed with 0.5 volumes of 25% propionic anhydride in acetonitrile. The reaction was pH adjusted to 8.0 with ammonium hydroxide and incubated for 15 min at 25 °C. The reaction was mixed with a further 0.5 volumes of fresh 25% propionic anhydride in acetonitrile and again adjusted to pH 8.0 with ammonium hydroxide. After incubation for 15 min at 25 °C, the reactions were concentrated by SpeedVac. The dried, propionylated samples were suspended in digest buffer (50 mM NH_4_ HCO_3_, pH 8.0) to 1 μg/μL and adjusted to pH 8.0 with ammonium hydroxide. Sequencing grade trypsin was added to 2% (mass/mass) of the total histone protein and incubated overnight at 25 °C. Afterward, trypsinization samples were concentrated by SpeedVac, and propionylation was repeated as described above. Dried samples were resuspended in 0.05% trifluoracetic acid, cleaned up using stage tips packed in house, and concentrated by a SpeedVac.

Samples were resuspended in 0.1% acetic acid and (20 ng/injection) analyzed on a Vanquish Neo LC (Thermo Fisher Scientific) coupled to an Orbitrap Ascend Tribrid mass spectrometer equipped with ETD, PTCR, and UVPD (Thermo Fisher Scientific). The LC was operated in trap-and-elute mode using a PepMap Neo C18, 100 Å, 5 μm, 0.3 × 5 mm Trap Cartridge (Thermo Fisher Scientific) and an Easy-Spray PepMap Neo C18, 100 Å, 2 μm, 75 μm × 150 mm column (Thermo Fisher Scientific) heated to 40 °C. The flow rate was 0.3 μL/min. Solvent A was 0.1% formic acid, and Solvent B was 0.1% formic acid in acetonitrile. The gradient consisted of 2 to 32% Solvent B in 46 min and 32 to 42% B in 7 min. The MS was operated in the data independent acquisition (DIA) mode. MS1 spectra were collected in the Orbitrap with 120k resolution and scan range 290–1200 *m*/*z*. HCD MS2 was performed with collision energy 25% and resolution 15k. The DIA method consisted of 34 fixed-width isolation windows of 24 *m*/*z* with 1 *m*/*z* overlap and center masses from 307 to 1093.25.

Raw data was analyzed in Skyline and further processed with in-house software EpiProfile.^55^ Each biological replicate of a treatment condition was analyzed separately. For comparison with middle-down results, site-specific PTM abundances from each replicate were averaged across all samples grouped for a single 6plex middle-down analysis. Pearson correlation was used to compare site-specific PTM abundance measurements between bottom-up and middle-down data.

### Nucleosome Reconstitution and Sortase Ligation

Into chilled nucleosome reconstitution buffer (final concentrations 2 M KCl, 10 mM Tris, pH 7.9 at 25 °C, 1 mM EDTA, 10 mM DTT), Widom 601 DNA was added to 6 μM, followed by octamer (6–10 μM, typically 6.6 μM). Small-scale nucleosome reconstitutions (50–100 μL) are carried out with new batches of the octamer and DNA to determine the optimal octamer concentration for reconstitution. For both small scale and preparative reconstitutions, the mixture of DNA and octamer are incubated for 30 min on ice and then transferred to prechilled dialysis cassettes. These were transferred to prechilled high-salt reconstitution buffer (10 mM Tris, pH 7.9 at 25 °C, 2 M KCl, 1 mM EDTA, 1 mM DTT; 500 mL for small scale; 1 L for large scale) and dialyzed over a linear salt gradient created by constant influx (1 mL/min for small scale; 2 mL/min for large scale) of low-salt reconstitution buffer (10 mM Tris, pH 7.9 at 25 °C, 150 mM KCl, 0.1 mM EDTA, 1 mM DTT; 2 L for small scale; 5 L for large scale) and efflux of buffer from the dialysis chamber, mediated by a dual channel peristaltic pump.^56^ This exchange occurs with constant vigorous mixing over 30–48 h, after which dialysis cassettes are transferred to low-salt reaction buffer for a final 2 h of dialysis.

The concentration of the crude nucleosome reconstitution within the dialysis cassette is determined by absorbance at 260 nm (nanodrop) and is directly used in the sortase ligation reaction. Reconstituted nucleosome (1.65 μM), followed by peptide (symmetric ligation: 49.5 μM; first asymmetric ligation: 16.5 μM; second asymmetric ligation: 49.5 μM), and then cW11 sortase (200 μM) are diluted into reaction buffer (40 mM Tris, pH 7.5 at 25 °C, 150 mM total NaCl and KCl, 5 mM CaCl_2_, 1 mM DTT final concentration; KCl from the reconstituted nucleosome and NaCl in the buffer are treated as equivalent for the purpose of determining the final concentration of monovalent cation chloride salt). For ligations employing a charged C-terminal auxiliary (e.g., tetra- and penta-acetylated histone tails), the nucleosome and peptide are sequentially added to reaction buffer and incubated for 10 min at 25 °C before the addition of cW11 sortase. Reactions are transferred to a 37 °C incubator for at least 4 h. Overnight reactions are generally well-tolerated, but should be avoided for hyperacetylated substrates. Reactions are quenched by the addition of one eq. of salmon sperm DNA (mass/mass, relative to nucleosome DNA; Fisher) and NaCl to 250 mM, mixing, then sitting for 5 min at 25 °C before purification.

Purification was accomplished by weak anion exchange (Tosoh DEAE 5 pW, 7.5 mm × 7.5 cm) liquid chromatography (Waters 1525 Binary HPLC Pump, 1 mL/min, mobile phase A: 10 mM Tris, pH 7.9 at 25 °C, 150 mM KCl, 0.5 mM EDTA; mobile phase B: 10 mM Tris, pH 7.9 at 25 °C, 600 mM KCl, 0.5 mM EDTA). Purification gradients by ligation type (symmetric vs asymmetric) and DNA length (147 bp vs 185 bp) are as follows:

147 bp, symmetric: 0–22% B (3 min), 22% B (7 min), 22–49% B (1 min), 49–65% B (21 min), 65–100% B (1 min), 100% (10 min), and 100–0% B (1 min)

147 bp, asymmetric: 0–22% B (3 min), 22% B (7 min), 22–49% B (1 min), 49–61% B (21 min), 65–100% B (1 min), 100% (10 min), and 100–0% B (1 min)

185 bp, symmetric: 0–22% B (3 min), 22% B (7 min), 22–63% B (1 min), 63–80% B (21 min), 65–100% B (1 min), 100% (10 min), and 100–0% B (1 min)

185 bp, asymmetric: 0–22% B (3 min), 22% B (7 min), 22–58% B (1 min), 63–75% B (21 min), 65–100% B (1 min), 100% (10 min), and 100–0% B (1 min)

Fractions collected during purification are directly diluted with one volume of chilled dilution buffer (10 mM Tris, pH 7.5 at 25 °C, 1 mM DTT) and spin concentrated (Amicon, 10 kDa MWCO). Fractions of purity may be assessed by SDS-PAGE followed by Western blotting against histone H3 (25 ng H3 loading, Abcam, ab1791) to confirm conversion of all H3 (aa33–135) to H3 (aa1–135) or blotting against relevant PTMs for asymmetric syntheses. Pooled, concentrated fractions are dialyzed against either dilution buffer (symmetric ligation) or low-salt reconstitution buffer (asymmetric ligation), and reaction yield is determined by absorbance at 260 nm (nanodrop). Following dialysis against low-salt reconstitution buffer, a second round of ligation and purification are performed as described above (note that the equivalents of peptide increase in the second round of ligation). Following dialysis into dilution buffer, symmetric and asymmetric nucleosomes are dialyzed into storage buffer (10 mM Tris, pH 7.5 at 25 °C, 25 mM NaCl, 1 mM DTT, 20% glycerol) and concentrated to >5 μM before flash freezing and storing at −80 °C.

## Supporting Information Available

Peptides identified and quantified in middle-down mass spectrometry (XLSX)
Experimental details; materials; additional methods; expression and purification of HDACs, sirtuins, LC, and LHC; peptide synthesis; cW11 sortase activity assays; peptide mass spectrometry; nucleosome mass spectrometry; nucleosome purification; Western blotting; calculated HDAC *V*/[*E*] values; nucleosome EM; asymmetric nucleosome Western blotting; asymmetric nucleosome EMSA assays (PDF)

## Abbreviations

Ac: acetyl
Pr: propionyl
Bu: butyryl
Cr: crotonyl
Lac: lactoyl
Suc: succinyl
Oct: octanoyl
Hib: alpha-hydroxyisobutyryl
Ub: ubiquitin
Me3: trimethyl
DEAE: diethylaminoethyl
