# Supplementary material for: Circular Engineered Sortase for Interrogating Histone H3 in Chromatin
Source: J Am Chem Soc. 2024 Nov 25;146(49):33914–27. doi: 10.1021/jacs.4c12585 (PMC11638967; doi:10.1021/jacs.4c12585)
Supplement: Supplementary file 2 — ja4c12585_si_002.pdf [file ja4c12585_si_002.pdf]

Supplemental Information

A circular engineered sortase for interrogating histone H3 in chromatin

Samuel D. Whedon [a≡], Kwangwoon Lee [a≡], Zhipeng A. Wang [a≡†], Emily Zahn [b], Congcong Lu [c‡], Maheeshi Yapa Abeywardana [a], Louise Fairall [d], Eunju Nam [a], Sarah DuBois-Coyne [a], Pablo De Ioannes [e], Xinlei Sheng [f], Adelina Andrei [a], Emily Lundberg [a], Jennifer Jiang [a], Karim-Jean Armache [e], Yingming Zhao [f], John W. R. Schwabe [d], Mingxuan Wu [a‖*], Benjamin A. Garcia [b*], Philip A. Cole [a*]

[a] Division of Genetics, Department of Medicine, Brigham and Women's Hospital; Department of Biological Chemistry and Molecular Pharmacology, Harvard Medical School, Boston, Massachusetts 02115, United States
[b] Department of Biochemistry and Molecular Biophysics, Washington University School of Medicine, St. Louis, MO 63110, USA
[c] Epigenetics Institute, Department of Biochemistry and Biophysics, Perelman School of Medicine, University of Pennsylvania, Philadelphia, PA, 19104, USA
[d] Leicester Institute of Structural and Chemical Biology, Department of Molecular and Cell Biology, University of Leicester, Leicester LE1 7RH, United Kingdom
[e] Department of Biochemistry and Molecular Pharmacology, New York University Grossman School of Medicine, New York, NY 10016, United States
[f] The Ben May Department for Cancer Research, University of Chicago, Chicago, IL 60637, United States
[†] Present address: Desai Sethi Urology Institute, Sylvester Comprehensive Cancer Center, University of Miami Miller School of Medicine, Miami, FL 33136, United States
[‡] Present address: Frontiers Science Center for Cell Responses, College of Life Sciences, Nankai University, Tianjin, 300071, China
[‖] Present address: Department of Chemistry, School of Science, Westlake University, Hangzhou 310030, China
* Email: wumingxuan@westlake.edu.cn
* Email: bagarcia@wustl.edu
* Email: pacole@bwh.harvard.edu
[≡] S.D.W., K.L. and Z.A.W. contributed equally.

Table of contents

1. Materials
2. Antibodies
3. Cloning
4. Protein expression and purification
5. Widom DNA production and purification
6. Peptide synthesis
7. Cryo-EM
8. Deacetylase kinetics
9. Demethylase kinetics
10. Electrophoretic mobility shift assays
11. Figure S1. Cleavage of purified, recombinant histone H3 by sortase mutants
12. Figure S2. Cleavage of semisynthetic, modified histone H3 by W11
13. Figure S3. SDS-PAGE analysis of sortase reaction in nuclear acid extracts
14. Figure S4. Sortase W11 mutant activity in the presence of co-solvents, chaotropes and detergents
15. Figure S5. Histone tails isolated by trichloroacetic acid precipitation of reaction protein components
16. Table S1. Abundance of individual H3 post-translational modifications in bottom-up and middle-down proteomics data
17. Figure S6. Characterization of H3(33-135) octamer overexpression and purification
18. Figure S7. Western blot analysis of cW11 nucleosome ligation
19. Figure S8. cW11 nucleosome ligation time course chromatograms
20. Figure S9. Mass spectrometric characterization of cW11 nucleosome ligation products

21. Figure S10. SDS-PAGE and TBE native gel characterization of cW11 nucleosome ligation products
22. Figure S11. Mass spectrometric characterization of peptide substrates used in cW11 nucleosome ligation
23. Figure S12. Characterization of asymmetric H3K4me2 H3K14ac ligation intermediate and final products
24. Figure S13. Comparison of Sirt6 deacetylase activity toward H3K9ac nucleosomes prepared by conventional reconstitution and cW11 nucleosome ligation
25. Figure S14. Comparison of LHC deacetylase activity toward H3K9ac nucleosomes prepared by conventional reconstitution and cW11 nucleosome ligation
26. Figure S15. Cryo-electron microscopy characterization of nucleosomes prepared by cW11 nucleosome ligation
27. Figure S16. Western blot measurement of Sirt1 activity by length of H3K9 acylation carbon chain.
28. Figure S17. Western blot measurement of Sirt2 activity by length of H3K9 acylation carbon chain.
29. Figure S18. Western blot measurement of Sirt6 activity by length of H3K9 acylation carbon chain.
30. Figure S19. Western blot measurement of MiDAC activity by length of H3K9 acylation carbon chain.
31. Table S6. Calculated V/[E] values for HDAC activity by length of H3K9 acylation carbon chain
32. Figure S20. Western blot measurement of Sirt1 activity toward four carbon acylations of H3K9
33. Figure S21. Western blot measurement of Sirt2 activity toward four carbon acylations of H3K9
34. Figure S22. Western blot measurement of Sirt6 activity toward four carbon acylations of H3K9
35. Figure S23. Western blot measurement of MiDAC activity toward four carbon acylations of H3K9
36. Figure S24. Western blot measurement of LHC activity toward four carbon acylations of H3K9
37. Figure S25. Western blot measurement of free HDAC activity toward four carbon acylations of H3K9
38. Table S7. Calculated V/[E] values for HDAC activity toward four carbon acylations of H3K9
39. Figure S26. Western blot measurement of Sirtuin5 activity toward acetylated and succinylated H3K9
40. Table S8. Calculated V/[E] values for HDAC activity toward H3K9 succinylation
41. Figure S27. Validation of H3Kac single site antibody specificity
42. Figure S28. Western blot measurement of Sirt2 activity toward mono-acetylated nucleosomes.
43. Figure S29. Western blot measurement of Sirt2 activity toward penta-acetylated nucleosomes.
44. Figure S30. Western blot measurement of Sirt6 activity toward mono- and penta-acetylated nucleosomes.
45. Figure S31. Western blot measurement of MiDAC activity toward mono- and penta-acetylated nucleosomes.
46. Figure S32. Western blot characterization of asymmetric mono/tetra-acetylated nucleosomes
47. Figure S33. Mass spectrometric characterization of asymmetric mono/tetra-acetylated nucleosomes and asymmetric mono-acetylated/unmodified nucleosomes.
48. Figure S34. Western blot measurement of Sirt2 activity toward asymmetrically acetylated nucleosomes
49. Figure S35. Western blot measurement of Sirt6 activity toward asymmetrically acetylated nucleosomes
50. Table S9. Calculated V/[E] values for Sirt2 activity toward mono-, penta-, and asymmetrically acetylated nucleosomes
51. Table S10. Calculated V/[E] values for Sirt6 activity toward mono-, penta-, and asymmetrically acetylated nucleosomes
52. Figure S36. Western blot measurement of MiDAC activity toward asymmetrically acetylated nucleosomes
53. Table S11. Calculated V/[E] values for MiDAC activity toward mono-, penta-, and asymmetrically acetylated nucleosomes

54. Figure S37. Western blot measurement of LC activity toward nucleosomes with asymmetric methylation and acetylation
55. Figure S38. Mass spectrometric characterization of ubiquitinated H3 peptides and ubiquitinated nucleosome
56. Figure S39. Western blot characterization of asymmetric unmodified/ubiquitinated nucleosome
57. Figure S40. Electrophoretic mobility shift assay titrating asymmetric nucleosomes with sfGFP-RFTS fusion
58. Figure S41. Electrophoretic mobility shift assay titrating sfGFP-RFTS fusion with asymmetric nucleosomes
59. References

Materials
Chemicals were purchased from Sigma-Aldrich, Fisher Scientific, Chem-Impex, Research Products International, or Ambeed and used without further purification. Fmoc protected amino acids were purchased from Sigma-Aldrich, Chem-Impex, Ambeed, P3 biosystems or ApexBio. Solvents were purchased from Sigma-Aldrich, Fisher Scientific, of EMD-Millipore. HDAC inhibitors MS-275 and Corin were purchased from MedChemExpress. Tandem mass tag reagents were provided by Thermo Scientific as part of the Tandem Mass Tag Systems Research Award. Media for bacterial culture was purchased from Sigma-Aldrich. Media for mammalian cell culture was purchased from Caisson Labs. Restriction enzymes were purchased from New England Biosciences. DNA isolation kits were purchased from Zymo. Polymerases were purchased from Agilent Technologies, Promega or New England Biosciences. Primers were purchased from Integrated DNA Technologies or Quintara Biosciences. Sanger sequencing was performed by Integrated DNA Technologies or Quintara Biosciences. Whole plasmid sequencing was performed by Quintara Biosciences or Plasmidsaurus. Columns for HPLC were purchased from Tosoh Biosciences (DEAE), Agilent technologies (C18), or Higgins Analytical (C18). Columns for FPLC were purchased from Cytiva. Automated solid-phase peptide synthesizer was purchased from Gyros Protein Technologies (Prelude).

Antibodies
For demethylase assays, anti-H3K4me2 (1:2000 dilution, Abcam, cat ab32356) and anti-H3 (1:2000 dilution, Abcam, cat ab1791) primary antibodies were used. For deacetylase assays, anti-H3K9ac (1:2000 dilution, Abcam AB32129), anti-H3K14ac (1:2000 dilution, EMD Millipore 07-353), anti-H3K18ac (1:2000 dilution, EMD Millipore 07-354), anti-H3K23ac (1:2000 dilution, EMD Millipore 07-355), anti-H3K27ac (1:2000 dilution, Cell Signaling 81735), anti-H3K9bu (1:2000 dilution, PTM Biolabs, PTM-305 need double check), anti-H3K9cro (1:2000 dilution, PTM Biolabs, PTM-539), pan anti-Khib (1:2000 dilution, A kind gift from Dr. Yingming Zhao, University of Chicago), anti-H3K9succ (1:1000 dilution, A kind gift from Dr. Yingming Zhao, University of Chicago), and anti-H3 (Abcam, #ab1791, 1:2000 dilution) primary antibodies were used. For all assays anti-rabbit IgG (1:1000 dilution, Cell Signaling Technology, cat 7074S) or anti-mouse IgG (1:1000 dilution, Cell Signaling Technology, cat 7076S) secondary antibodies were used.

Cloning
Sortase mutants were derived from previously reported variants of S. aureus sortase A (Srt A): F40 sortase mutant, enhanced sortase pentamutant (eSrt), and cyclic sortase.[1–3] Additional mutations were identified using FireProt webserver.[4] DNA encoding S. aureus Srt A (60-206) (A kind gift of Dr. Dirk Schwarzer) was cloned into pET21 vector (Ampicillin resistant). Subsequent mutations were accomplished by sequential rounds of quick-change site directed mutagenesis. The PCR products were purified by PCR cleanup kit (Zymo), treated with DpnI (New England Biolabs), and transformed into DH5anti E. coli. Single colonies were picked and grown over night in 5 mL of Luria Bertani media supplemented with ampicillin (100 µg / mL), then pelleted (5 minutes, 4500 rcf, 4 ˚C). Plasmids were obtained from cell pellets by mini-prep (Plasmid Miniprep Classic, Zymo) and Sanger sequenced, or sequenced by nano-pore.

Sortase F40 (mutations distinguishing F40 from Srt A are underlined)
MQAKPQIPKDKSKVAGYIEIPDADIKEPVYPGPATPEQLNRGVSFAEENESLDDQNISIAGHTFIDRPNYQF
TNLKAAKMGSMVYFKVGNETRKYKMTSIRDVKPQDVGMHLAEKGKDKQLTLITCDDYNEKTGVWEKRKI
FVATEVKLEHHHHHH

Sortase W6 (mutations distinguishing F40 from Srt A are underlined, mutations from eSrt are ***bolded & italicized***)
MQAKPQIPKDKSKVAGYIEIPDADIKEPVYPGPAT***S***EQLNRGVSFAEENESLDDQNISIAGHTFIDRPNYQF
TNLKAAKMGSMVYFKVGNETRKYKMTSIR***N***VKPT***A***VMHLAEKGKDKQLTLITCDDYNEKTGVWEKRKIFV
ATEVKLEHHHHHH

Sortase W8 (mutations distinguishing F40 from Srt A are underlined, mutations from eSrt are ***bolded & italicized***)

MQAKPQIPKDKSKVAGYIEIPDADIKEPVYPGPATPEQLNRGVSFAEENESLDDQNISIAGHTFIDRPNYQF
TNLKAAKMGSMVYFKVGNETRKYKMTSIR***N***VKPQDVMHLAEKGKDKQLTLITCDDYNE***E***TGVWE***T***RKIF
VATEVKLEHHHHHH

Sortase W9 (mutations distinguishing F40 from Srt A are underlined, mutations from eSrt are ***bolded & italicized***)
MQAKPQIPKDKSKVAGYIEIPDADIKEPVYPGPAT***S***EQLNRGVSFAEENESLDDQNISIAGHTFIDRPNYQF
TNLKAAKMGSMVYFKVGNETRKYKMTSIR***N***VKPQDVMHLAEKGKDKQLTLITCDDYNEKTGVWE***T***RKIF
VATEVKLEHHHHHH

Sortase W11 (mutations distinguishing F40 from Srt A are underlined, mutations from eSrt are ***bolded & italicized***)
MQAKPQIPKDKSKVAGYIEIPDADIKEPVYPGPAT***S***EQLNRGVSFAEENESLDDQNISIAGHTFIDRPNYQF
TNLKAAKMGSMVYFKVGNETRKYKMTSIR***N***VKPQDVMHLAEKGKDKQLTLITCDDYNE***E***TGVWE***T***RKIF
VATEVKLEHHHHHH

Sortase W12 (mutations distinguishing F40 from Srt A are underlined, mutations from eSrt are ***bolded & italicized***)
MQAKPQIPKDKSKVAGYIEIPDADIKEPVYPGPAT***S***EQLNRGVSFAEENESLDDQNISIAGHTFIDRPNYQF
TNLKAAKMGSMVYFKVGNETRKYKMTSIR***N***VKPT***A***VMHLAEKGKDKQLTLITCDDYNEKTGVWE***T***RKIFV
ATEVKLEHHHHHH

Sortase W13 (mutations distinguishing F40 from Srt A are underlined, mutations from eSrt are ***bolded & italicized***)
MQAKPQIPKDKSKVAGYIEIPDADIKEPVYPGPAT***S***EQLNRGVSFAEENESLDDQNISIAGHTFIDRPNYQF
TNLKAAKMGSMVYFKVGNETRKYKMTSIR***N***VKPQ***A***VMHLAEKGKDKQLTLITCDDYNE***E***TGVWE***T***RKIF
VATEVKLEHHHHHH

Sortase W15 (mutations distinguishing F40 from Srt A are underlined, mutations from eSrt are ***bolded & italicized***)
MQAKPQIPKDKSKVAGYIEIPDADIKEPVYPGPAT***R***EQLNRGVSFAEENESLDDQNISIAGHTFIDRPNYQF
TNLKAAKMGSMVYFKVGNETRKYKMTSIR***N***VKPQDVMHLAEKGKDKQLTLITCDDYNE***E***TGVWE***T***RKIF
VATEVKLEHHHHHH

Sortase W16 (mutations distinguishing F40 from Srt A are underlined, mutations from eSrt are ***bolded & italicized***)
MQAKPQIPKDKSKVAGYIEIPDADIKEPVYPGPAT***R***EQLNRGVSFAEENESLDDQNISIAGHTFIDRPNYQF
TNLKAAKMGSMVYFKVGNETRKYKMTSIR***N***VKPQAVMHLAEKGKDKQLTLITCDDYNEKTGVWE***T***RKIFV
ATEVKLEHHHHHH

Sortase W11 E108Q S116V (mutations distinguishing F40 from Srt A are underlined, mutations from eSrt are ***bolded & italicized***, mutations from FireProt are **bolded**)
MQAKPQIPKDKSKVAGYIEIPDADIKEPVYPGPAT***S***EQLNRGVSFAEEN**Q**SLDDQNI**V**IAGHTFIDRPNYQF
TNLKAAKMGSMVYFKVGNETRKYKMTSIR***N***VKPQDVMHLAEKGKDKQLTLITCDDYNE***E***TGVWE***T***RKIF
VATEVKLEHHHHHH

Sortase cW11 (Split intein is *italicized*, linkers are **bolded** and W11 E108Q S116V is underlined)
*MIKIATRKYLGKQNVYDIGVERDHNFALKNGFIASN***CFNGGSS**MQAKPQIPKDKSKVAGYIEIPDADIKEPV
YPGPATSEQLNRGVSFAEENQSLDDQNIVIAGHTFIDRPNYQFTNLKAAKMGSMVYFKVGNETRKYKMTS
IRNVKPQDVMHLAEKGKDKQLTLITCDDYNEETGVWETRKIFVATEVKLEHHHHHH**AEY***CLSYETEILTVE
YGLLPIGKIVEKRIECTVYSVDNNGNIYTQPVAQWHDRGEQEVFEYCLEDGSLIRATKDHKFMTVDGQML
PIDEIFERELDLMRVDNLPN*

Protein expression and purification
C-terminally truncated ubiquitin (aa1-75) was obtained by expression of a C-terminal Ava intein fusion construct (A kind gift from Dr. Champak Chatterjee, University of Washington) from BL21(DE3) E. coli. An

overnight starter culture of Luria-Bertani medium (Sigma) with ampicillin (100 mg/L) was grown overnight at 37 °C, of which 10 mL was used to inoculate each 1 L of the same medium used for overexpression. Cells were grown to an OD600 of 0.6-0.8, then chilled on ice and induced with 0.3 mM IPTG for 16 hours at 16 °C. Cells were harvested by centrifugation (4 k x g, 30 min, 4 °C), and the cell pellet was resuspended in 5 volumes of chilled (4 °C) lysis buffer (50 mM sodium phosphate, pH 8.0, 300 mM NaCl, 5 mM imidazole, 1 mM beta-mercaptoethanol). Resuspended pellets were dounced to uniformity and lysed by three passages through a microfluidizer. The supernatant was cleared by centrifugation (12 k x g, 30 min, 4 °C) and the supernatant was passed over 5 mL Ni NTA agarose twice by gravity. Resin was washed with 20 CV lysis buffer, then washed with 20 CV wash buffer (50 mM sodium phosphate, pH 8.0, 300 mM NaCl, 20 mM imidazole, 1 mM beta-mercaptoethanol), and eluted with 6 CV of elution buffer (50 mM sodium phosphate, pH 8.0, 300 mM NaCl, 250 mM imidazole, 1 mM beta-mercaptoethanol). Elution fractions were pooled and dialyzed three times against dialysis buffer (100 mM sodium phosphate, pH 7.2, 150 mM NaCl, 1 mM EDTA, 1 mM sodium 2-mercaptoethanesuflonate) at 4 °C. Thiolysis was initiated by recovering the dialyzed solution, bringing it to 100 mM in sodium 2-mercaptoethanesulfonate while maintaining the pH at 7.2, then moving to a 30 °C incubator. Thiolysis was monitored by RP-HPLC, and ESI-MS, and was complete within 12 hours. Thiolyzed mutant ubiquitin (aa1-75)-thioester was dialyzed into 0.05% trifluoroacetic acid at 4 °C, then freeze dried, resuspended in a minimal volume and separated from Ava intein by RP-HPLC.[5]

The fusion construct of superfolder GFP and human DNMT1 RFTS domain (aa246-495; sfGFP-RFTS) (from LOBSTR Rosetta E. coli) was grown from an overnight starter culture (37 °C) of LB medium (Sigma) with kanamycin (50 mg/L) and chloramphenicol (34 mg/L). Growth for overexpression was initiated by adding 10 mL of starter to inoculate each 1 L of the same medium. Cells were grown to an OD600 of 0.6-0.8, then chilled on ice and induced with 0.3 mM IPTG for 16 hours at 16 °C. Cells were harvested by centrifugation (4 k x g, 30 min, 4 °C), and the cell pellet was resuspended in 5 volumes of chilled (4 °C) lysis buffer (50 mM Tris, pH 7.5 at 25 °C, 300 mM NaCl, 0.5 mM TCEP, 10% glycerol). Resuspended pellets were dounced to uniformity and lysed by three passages through a microfluidizer. Supernatant was cleared by centrifugation (12 k x g, 30 min, 4 °C) and the supernatant was passed over 1 mL Ni NTA agarose twice by gravity. Resin was washed with 20 CV lysis buffer, 10 CV wash buffer (50 mM Tris, pH 7.5 at 25 °C, 300 mM NaCl, 0.5 mM TCEP, 10% glycerol, 25 mM imidazole), and eluted with 10 CV elution buffer (50 mM Tris, pH 7.5 at 25 °C, 300 mM NaCl, 0.5 mM TCEP, 10% glycerol, 200 mM imidazole). Elution fractions were checked by Coomassie-stained SDS-PAGE and purified fractions were pooled, and concentrated (Amicon, 30 kDa MWCO). Concentrated sfGFP-RFTS was further purified by size exclusion chromatography by FPLC (superdex200) run in lysis buffer, after which fractions were again checked by SDS-PAGE, pooled, concentrated as before, aliquoted and flash frozen.

His-SUMO-Sirt2 (aa56-356) was prepared as previously described.[6] Briefly His-SUMO-Sirt2 (from BL21 (DE3) E. coli) was grown at 37 °C from an overnight starter culture of Luria-Bertani (LB) medium (Sigma) with kanamycin (50 mg/L) and chloramphenicol (35 mg/L), of which 10 mL was used to inoculate each 1 L of the same medium used for overexpression. Cells were grown with constant shaking (200 rpm) at 37 °C to an OD600 of 0.6-0.8. Culture temperature was dropped to 16 °C, then induced with 0.5 mM IPTG for 16 hours at 16 °C. Cells were harvested by centrifugation (4 k x g, 15 min, 4°C), and the cell pellet was resuspended in 5 volumes of chilled (4 °C) lysis buffer (20 mM Tris, pH 7.8 at 25 °C, 500 mM NaCl, 20 mM imidazole, and 0.5 mM TCEP). Resuspended cell pellets were lysed by three passages through a French press, and the cell lysate was clarified by centrifugation (12,000 x g, 4°C, 30 min). The lysate supernatant was incubated with pre-equilibrated Ni-NTA resin (2 mL resin bed volume/1 L culture) for 1 hour at 4°C. Resin was drained and washed with 10 column volumes (CV) of wash buffer (20 mM Tris, pH 7.8 at 25 °C, 500 mM NaCl, 50 mM imidazole, and 0.5 mM TCEP), followed by 25 CV of high-salt wash buffer (20 mM Tris, pH 7.8 at 25 °C, 2 M NaCl, 20 mM imidazole, and 0.5 mM TCEP), 25 CV of lysis buffer, and eluted with 10 CV of elution buffer (20 mM Tris, pH 7.8 at 25 °C, 500 mM NaCl, 250 mM imidazole, and 0.5 mM TCEP). The elution was concentrated (Amicon, 10 kDa MWCO, 4000 rpm, 4°C), mixed with UlpI protease (1:100 mass:mass, UlpI:Sirt2) to remove the His-SUMO tag, and dialyzed into dialysis buffer (20 mM Tris, pH 7.5 at 25 °C, 150 mM NaCl, 0.5 mM TCEP) overnight at 4°C. UlpI was depleted by re-incubation with pre-equilibrated Ni-NTA resin for 1 hour at 4°C, after which the the flow-through was collected and concentrated (Amicon, 30 kDa MWCO, 4000 rpm, 4°C). The concentrated flow through was purified by Superdex200 in SEC buffer (50 mM Tris, pH 7.5 at 25 °C, 150 mM NaCl, 0.5 mM TCEP). Fractions were evaluated by SDS-PAGE, pooled, and concentrated using an (Amicon, 10 kDa,

4000 rpm, 4°C). Sample concentration was determined by densitometry, after which protein was aliquoted, flash-frozen in liquid nitrogen, and stored at -80°C until use.

His-TEV-Sirt6 (aa1-355) was prepared as previously described.[7] Briefly, His-TEV-Sirt6 (from LOBSTR Rosetta E. coli) was grown at 37 °C from an overnight starter culture of Luria-Bertani (LB) medium (Sigma) with kanamycin (50 mg/L), of which 10 mL was used to inoculate each 1 L of the same medium used for overexpression. Cells were grown with constant shaking (200 rpm) at 37 °C to an OD600 of 0.6-0.8. Culture temperature was dropped to 25 °C, then induced with 0.5 mM IPTG for 18 hours at 25 °C. Cells were harvested by centrifugation (4 k x g, 15 min, 4°C), and the cell pellet was resuspended in chilled (4 °C) lysis buffer (20 mM Tris, pH 7.5 at 25 °C, 500 mM NaCl, 20 mM imidazole, 30 mL buffer / 1 L culture). Resuspended cell pellets were lysed by three passages through a French pressure cell, and the cell lysate was clarified by centrifugation (12,000 x g, 4°C, 30 min). The lysate supernatant was incubated with pre-equilibrated Ni-NTA resin (2 mL resin bed volume/1 L culture) for 1 hour at 4°C. Resin was drained and washed with 10 CV of wash buffer (20 mM Tris, pH 7.5 at 25 °C, 500 mM NaCl, 50 mM imidazole 0.5 mM TCEP), followed by 25 CV of high-salt wash buffer (20 mM Tris, pH 7.5 at 25 °C, 2 M NaCl, 20 mM imidazole, 0.5 mM TCEP), 25 CV of lysis buffer, and eluted with 10 CV of elution buffer (20 mM Tris, pH 7.5 at 25 °C, 500 mM NaCl, 250 mM imidazole, and 0.5 mM TCEP). The elution was concentrated (Amicon, 10 kDa MWCO, 4,000 rpm, 4 °C), mixed with TEV protease (1:100 mass:mass, TEV:Sirt6) to remove the His-TEV tag, and dialyzed overnight into dialysis buffer (20 mM Tris, pH 7.5 at 25 °C, 150 mM NaCl, 20 mM imidazole, 0.5 mM TCEP). His-TEV and uncleaved His-TEV-Sirt6 were depleted by re-incubation with pre-equilibrated Ni-NTA resin for 1 hour at 4°C, after which the flow-through was collected, diluted with excess Heparin buffer A (50 mM Tris, pH 7.5 at 25 °C, 150 mM NaCl, 5 % glycerol, 0.5 mM TCEP), and concentrated (Amicon, 30 kDa MWCO, 4000 rpm, 4°C). The concentrated sample was purified by heparin column (1 mL, Cytiva, #17040601), and eluted with a linear gradient from 0-50 % heparin buffer B (50 mM Tris, 2000 mM NaCl, 5 % glycerol, 0.5 mM TCEP, pH 7.5) over 20 column volumes (CV) at a flow rate of 0.6 mL/min. Fractions were evaluated by SDS-PAGE, pooled, and concentrated using an (Amicon, 10 kDa, 4000 rpm, 4°C). Concentrated heparin elution further purified Superdex200 (GE, #28-9909-44) with Superdex running buffer (50 mM Tris, pH 7.5, 150 mM NaCl, 0.5 mM TCEP). Fractions were again evaluated by SDS-PAGE, pooled, and concentrated using an (Amicon, 10 kDa, 4000 rpm, 4°C). Sample concentration was determined by densitometry, after which protein was aliquoted, flash-frozen in liquid nitrogen, and stored at -80°C until use.

For the demethylase assays, LSD1-CoREST1 (LC) complex was prepared. His6-tagged LSD1 (aa171-852) and His6-tagged CoREST1 (aa286-482), subcloned in pET15b and pET28a vectors respectively, were co-transformed into BL21-CodonPlus (DE3)-RIPL competent cells (Agilent). Transformed cells were inoculated in LB media containing 100 mg/L ampicillin, 50 mg/L kanamycin, and 35 mg/L chloramphenicol at 37°C. The overnight starter culture was subsequently inoculated into a larger volume of LB media. When the culture reached an A600 of 0.6, 0.5 mM isopropyl β-D-1-thiogalactopyranoside (IPTG) was added to induce protein expression at 18°C for 20 hours. After induction, cells were harvested by centrifugation at 2,702g for 10 minutes at 4°C. The cell pellets were resuspended in lysis buffer containing 20 mM Tris (pH 7.8), 200 mM NaCl, 20 mM imidazole, and 0.5 mM tris(2-carboxyethyl)phosphine (TCEP), then lysed using a french press. The lysate was centrifuged at 20,853g for 30 minutes, and the supernatant was incubated with nickel-nitrilotriacetic acid (NiNTA) resin (MCLAB) for 1 hour at 4°C. The resin was washed with lysis buffer and the bound proteins were eluted with lysis buffer containing 200 mM imidazole. The eluates were concentrated and buffer-exchanged against size exclusion chromatography (SEC) buffer (20 mM Tris, pH 7.8, 200 mM NaCl, and 0.5 mM TCEP) using a 10 kDa MWCO concentrator (Amicon, Millipore Sigma). The buffer-exchanged eluates were further purified by FPLC, using a Superdex 200 Increase 10/300 GL size exclusion column (Cytiva) pre-equilibrated with SEC buffer to obtain stoichiometric complexes. The final LC complex, verified to be >90% pure by SDS-PAGE and Coomassie staining, was collected from the major peak (around 12.5 mL elution volume), pooled, and quantified by A280 absorbance for enzymological studies without further concentration. LC protein solutions were flash-frozen and stored at -80°C.

For the deacylase assays, LSD1-CoREST1-HDAC1 (LHC) was expressed and purified following previously reported protocols.[8–10] Full-length human LSD1, N-terminally 3X FLAG-tagged CoREST1 (aa84-482), and full-length human HDAC1 in pcDNA3.1 vectors were co-transfected into HEK293F cells using polyethylenimine (PEI) (Millipore Sigma, cat 408727-100ML). After 48 hours of transfection, the transfected HEK293F cells were harvested by centrifugation at 2,702g for 10 minutes, resuspended in lysis buffer (50 mM HEPES, pH 7.5, 100 mM KCl, 0.3% Triton X-100, 5% glycerol, and a protease

inhibitor cocktail (Thermo Fisher Scientific)), lysed by sonication, and centrifuged at 20,853g for 30 minutes at 4°C. The supernatant was purified using anti-FLAG M2 affinity resin (Millipore Sigma). The FLAG tag on CoREST1 was removed by treatment with TEV protease (1:100 M/M TEV to CoREST1 ratio). Further purification was carried out using gel filtration on a Superose 6 10/300 GL column (Cytiva) pre-equilibrated with buffer containing 50 mM HEPES (pH 7.5), 50 mM KCl, and 0.5 mM TCEP. The pooled fractions from the main peak around 13 mL elution volume were concentrated down to approximately 5 μM using a concentrator with a 30 kDa MWCO (Pall). The LHC complex showed a 1:1:1 stoichiometry and was >90% pure, as confirmed by SDS-PAGE with Coomassie staining. The LHC protein samples were aliquoted, flash-frozen, and stored at -80°C.

The expression and purification of MiDAC (DNTTIP1, MIDEAS and HDAC1) was performed as described previously.[11] Plasmids (pcDNA3) encoding DNTTIP1, HDAC1, and a N-terminally 10xHis-3xFLAG-TEV tagged MIDEAS were co-transfected into HEK293F cells with PEI (Sigma). After 48 hours of growth, cells were resuspended in resuspension buffer (50 mM Tris, pH 7.5, 100 mM potassium acetate, 10% (v/v) glycerol, 0.5% (v/v) Triton X-100, Complete EDTA-free protease inhibitor (Roche)) and lysed by sonication. Insoluble material was removed by centrifugation, and the supernatant was passed over anti-FLAG immunoaffinity resin. The bound complex was washed twice with resuspension buffer, three times with wash buffer (50 mM Tris/Cl pH 7.5, 50 mM potassium acetate, 5% (v/v) glycerol, 0.5 mM TCEP), and then incubated with RNaseA for 1 h at 4 °C. After RNAse incubation the resin was washed a further five times with wash buffer. MiDAC complex was liberated from the resin by overnight incubation with TEV protease on a roller at 4 °C. The mixture was purified further using gel filtration on a Superdex 200 Increase 10/ 300 GL column (run in 25 mM HEPES, pH 7.5, 50 mM potassium acetate, 0.5 mM TCEP). The purified complex was concentrated to 1–5 mM and analyzed by SDS-PAGE stained with Coomassie to confirm purity (>80 %). Glycerol was added to 10% (final concentration), then samples were aliquoted, flash frozen, and stored at -80 °C until use.

HDAC1 was purchased from BPS Bioscience (cat 50051). Sirt1 was purchased from BPS Bioscience (cat 50081). Sirt5 was purchased from BPS Bioscience (cat 50085).

Widom DNA production and purification

Widom 601 DNAs (147 and 185 bp) were produced from multi-copy plasmids (16 x 147, 12 x 185) isolated from transformed DH5α E. coli grown for 36-48 h in CircleGrow media supplemented with antibiotic (147 bp: Ampicillin; 185 bp: Kanamycin). Cells were harvested by centrifugation (4 k x g, 30 min, 4 °C), and the cell pellet was resuspended in 5 volumes of lysis buffer (10 mM Tris, pH 8 at 25 °C, 10 mM EDTA). Lysis was initiated with lysozyme, after which 0.8 volumes of alkaline lysis buffer (2% SDS, 0.2 M NaOH) was added and the sample was mixed by inverting. One volume of neutralization buffer (1.5 M potassium acetate / acetic acid (pH 4.9)) was added and the sample was mixed by inverting, then insoluble material was removed by centrifugation. Clarified supernatant was filtered, then crude product was precipitated with isopropanol (0.6 volumes). Crude product was resuspended in 10 mM Tris (pH 8), 10 mM EDTA, and one volume of 5 M LiCl was added to precipitate RNA and proteins. The solution was clarified by centrifugation and the supernatant was combined with isopropanol (0.6 volumes) to precipitate crude product. The crude product was again resuspended in 10 mM Tris (pH 8), 1 mM EDTA and treated with RNase A, before precipitating again with isopropanol (3 volumes). The pelleted plasmid was resuspended in 10 mM Tris (pH 8), 1 mM EDTA, and digested with EcoRV in CutSmart buffer (New England Biolabs) at 37 °C. Insert DNA was purified by diluting the reaction 10-fold with dilution buffer (10 mM Tris, pH 8 at 25 °C, 1 mM EDTA) and passing twice over Sepharose fast-flow Q resin. The resin was washed with 20 volumes of wash buffer (10 mM Tris, pH 8 at 25 °C, 300 mM NaCl, 1 mM EDTA) and eluted with 5 volumes of elution buffer (10 mM Tris, pH 8 at 25 °C, 700 mM NaCl, 1 mM EDTA) then concentrated and stored at -20 °C.

Peptide synthesis

Histone H3.1 peptides (aa1-34) and oligoglycine tandem mass tag peptides were prepared by Fmoc-based solid phase peptide synthesis with modified amino acids at the relevant positions. Peptides were synthesized using a Prelude Peptide Synthesizer (Gyros Protein Technologies) using a standard deprotection and coupling protocol (vide infra). Modified amino acids were installed by either the standard protocol, or one of the following manual coupling protocols:

Standard deprotection: 20% piperidine in DMF, 2 rounds of 10 minutes, then 7 DMF washes.

Standard coupling: 4 eq. AA, 3.75 eq. HATU, 0.2 M NMM in DMF, 2 rounds of 90 minutes, then 5 DMF washes.
Tandem Mass Tag: Nα-Fmoc-Nβ-ivDde-2,3-diaminopropionic acid; standard coupling, followed by capping with 10 eq. acetic anhydride and 10 eq. DIEA.
Acetylation: Fmoc-K(Ac)-OH; standard coupling.
Dimethylation: Fmoc-K(Me2)-OH; 2 eq., 1.75 eq HATU, 4 eq DIEA, 1 round, 90 minutes, followed by capping with 10 eq. acetic anhydride and 10 eq. DIEA.
Trimethylation – Fmoc-K(Me3)-OH HCl; 2 eq., 1.75 eq HATU, 4 eq DIEA, 2 rounds, 90 minutes, followed by capping with 10 eq. acetic anhydride and 10 eq. DIEA.
Acylations – Fmoc-K(alloc)-OH; standard coupling.
Ubiquitination – Fmoc-K(Boc-Cys(Trt))-OH; 2 eq., 1.75 eq HATU, 4 eq DIEA, 2 rounds, 90 minutes, followed by capping with 10 eq. acetic anhydride and 10 eq. DIEA.

In each case Ala1 of the H3 sequence was coupled as Boc-Ala-OH. Deprotection of K(alloc) was accomplished with Pd(PPh3)4 (0.35 eq) and phenylsilane (20 eq) in anhydrous dichloromethane for 2 rounds of 60 minutes. Subsequent acylation was achieved with 4 eq. carboxylic acid, 3.75 eq. HATU, and 16 eq. DIEA in DMF for 2 rounds of 60 minutes.

In synthesis of the tandem mass tag (TMT) peptide each standard coupling was followed by capping with 4 eq. 3-phenylpropionic acid, 3.75 eq. HATU, 0.2 M NMM in DMF, for one round of 20 minutes. The most N-terminal glycine in the TMT peptide was coupled as Boc-Gly-OH. Deprotection of the 2,3-diaminopropionic acid Nβ-ivDde was accomplished with 5% hydrazine in DMF for ten rounds of 5 minutes, followed by 5 rounds of washing with DMF, and one round of washing with dichloromethane. The resin was subsequently divided into six fractions of approximately equal mass, each of which was treated with 1 eq. of TMT NHS ester reagent resuspended in anhydrous DMF, to which was added 4 eq. of silica plug dried DIEA.

Peptides were cleaved (90% trifluoroacetic acid, 5% water, 5% triisopropylsilane) and ether precipitated. Histone tail peptides were resuspended in 0.05% trilfuoroacetic acid and purified by preparative RP-HPLC (C18; mobile phase A: water + 0.05% TFA; mobile phase B: acetonitrile +0.05% TFA; 7-40% B 40 minute gradient). TMT peptides were resuspended in mobile phase A and purified by analytical RP-HPLC (Eclipse XDB-C18 4.6 x 250 mm, 0% B isocratic 13 minutes, 0-95%B 15 minutes). Fraction purity was assessed by ESI-MS (Thermo Q Exactive), and the purest fractions were pooled. H3 tail peptides were quantified by analytical RP-HPLC against a standard curve prepared from unmodified H3(1-34) peptide.

Ubiquitinated peptides were prepared by denaturing expressed protein ligation of ubiquitin (aa1-75)-mesna thioester (2.5 mM) with H3 (aa1-34) peptide (0.5 mM) in ligation buffer (250 mM HEPES, pH 7.5, 6 M Guanidine HCl, 30 mM TCEP, 30 mM methylthioglycolate) under argon. Reactions were mixed overnight at 37 °C and checked by analytical RP-HPLC followed by ESI-MS and SDS-PAGE. Once judged complete, one volume of desulfurization buffer (250 mM HEPES, pH 7, 6 M guanidine HCl, 500 mM TCEP, 60 mM VA-044) was added, and the reaction was placed under argon before mixing overnight at 37 °C.[12] Unligated peptide was removed by RP-HPLC (Higgins Analytical, C18; 30-37% B gradient), then fractions containing the ligation product were identified by ESI-MS and SDS-PAGE and pooled. Ligated peptide did not separate from hydrolyzed ubiquitin (aa1-75), and was quantified by SDS-PAGE relative to a BSA standard curve.

Cryo-EM

In-situ fixation of cW11 nucleosome for structural studies was performed according to Worden.[13] 300 mM nucleosome solution was dialyzed against crosslink buffer (25 mM HEPES pH 7.5, 25 mM NaCl, 1 mM EDTA,1 mM DTT) and mixed with 2.44 ml of 0.14% glutaraldehyde. The crosslinking reaction was incubated on ice for 60 min. The reaction was quenched for 1 hour on ice by adding 1.0 M Tris pH 7.5 to the final concentration of 100 mM. Finally, the sample was dialyzed against crosslink buffer and concentrated using an Amicon Ultra 30K MWCO spin concentrator to a final 0.2 mg/ml concertation, according to DNA absorption at 260 nm.

Cryo-EM grids of the cW11 nucleosome were prepared following an established protocol: 3.0 µL of the samples at 0.2 mg/mL were applied to glow-discharged Quantifoil gold grids (400 mesh, 1.2 µm hole size).[14] The grids were then blotted for 3 seconds at 4˚C and 100% humidity and plunge-frozen using Vitrobot Mark IV (FEI Company). All sample images were recorded on FEI Talos Arctica operated at 200kV using Gatan K2 Summit direct electron detector camera in counting mode at a nominal

magnification of 130,000x (calibrated pixel size of 1.096 Å/pixel). The total accumulated electron exposure was 50.77 electrons per Å2.

Raw Cryo-EM Images were motion corrected using UCSF MotionCor2 v1.2.1; Patch CTF was used to calculate CTF, Blob Picker for particle picking, Extract Micrograph for particle extraction, 2D classification, Ab Initio for Ab Initio reconstruction and hetero, homo and Non-homogeneous refinement for 3D refinement; all implemented on CryoSPARC 4.5.1.[15,16] This procedure led to obtaining reconstructions of cW11 nucleosome at 4.7 Å (FSC = 0.143).

For the model fitting of the canonical nucleosome into the cryo-EM map, we fitted the X-ray structure of the X. Laevis Nucleosome Core Particle (PDBID:1KX5)[17] using the map fit feature included in ChimeraX 1.4.[18] All structure images were generated using ChimeraX 1.4.

Deacylase kinetics

All deacetylation assays and analyses were performed as previously reported, these include deacetylation of symmetric H3 penta- and mono-acetylated nucleosomes (MiDAC, Sirt2, and Sirt6), deacetylation of asymmetric H3 mono-acetylated/unmodified or H3mono-acetylated/tetra-acetylated nucleosomes (MiDAC, Sirt2, and Sirt6), deacylation of symmetric H3 acylated nucleosomes, and comparative deacetylation of nucleosomes prepared by established methods and the sortase ligation method (LHC and Sirt6). Symmetric nucleosomes (H3K9ac, H3K14ac, H3K18ac, H3K23ac, H3K27ac, H3pentaAc, H3K9pr, H3K9bu, H3K9cro, H3K9hib, H3K9succ) and asymmetric nucleosomes (H3K18ac, H3K23ac, H3K27ac) (final 100 nM) were diluted into HDAC reaction buffer (50 mM HEPES at pH 7.5, 100 mM KCl, 0.2 mg/mL BSA, and 100 µM inositol hexaphosphate (IP6)), or Sirtuin reaction buffer (50 mM HEPES at pH 7.5, 1 mM DTT, 0.2 mg/mL BSA, and 1 mM NAD). The reaction solution was kept on ice until the addition of HDAC complex (LHC, MiDAC, HDAC1 free enzyme) or Sirtuin (Sirt1, Sirt2, Sirt5, Sirt6) then incubated at 37 ˚C. Each complex was assayed at a minimum of two enzyme concentrations to ensure consistency in calculated V/[E] values. At each assay time points, replicate aliquots were taken from a single reaction, and quenched. Typically, 6.5 µL aliquots of the reaction were taken and quenched with Dual quenching buffer (6.5 µL of a 1:1 mixture of 4 × Laemmli sample buffer and 40 mM EDTA) such that the final quenched aliquot contains 1 × Laemmli sample buffer with 10 mM EDTA. For typical nucleosome assays, time points were collected at 0, 30, 60, 90 and 120 min. Each sample was then boiled for 3-5 min at 95 ˚C and resolved on a 4-20 % gradient SDS-PAGE gel (TGXTM, Bio-Rad, 4561096) at 180 Volts for ~30 min. Gels were then transferred to nitrocellulose membrane (Transfer Stack, Invitrogen, IB301031) for western blot analysis (WB) by iBlot (Invitrogen) with P3 (20 V) for 5.0 min. Membranes were blocked for 60 minutes with 5% BSA in TBST (20 mM Tris pH 7.5 at 25 C, 150 mM NaCl, 0.1% Tween-20) then incubated overnight at 4 ˚C with primary antibody in 5% BSA in TBST. Site-specific antibodies for acetylated H3 and acylated H3 (1:2000 unless noted otherwise), such as for anti-H3K9ac (Abcam AB32129), anti-H3K14ac (EMD Millipore 07-353), anti-H3K18ac (EMD Millipore 07-354), anti-H3K23ac (EMD Millipore 07-355), anti-H3K27ac (Cell Signaling 81735),, anti-H3K9bu (PTM Biolabs, PTM-305), anti-H3K9cro (PTM Biolabs, PTM-539), pan anti-Khib (A kind gift from Dr. Yingming Zhao, University of Chicago, 1:1000 dilution), and anti-H3K9succ (A kind gift from Dr. Yingming Zhao, University of Chicago, 1:1000 dilution) were used to blot the corresponding membranes. Meanwhile, anti-H3 (Abcam, #ab1791, 1:2000 dilution) was used to visualize total H3. The specificities of the primary antibodies against H3K9ac, H3K14ac, H3K18ac, H3K23ac, and H3K27ac were validated on both H3 mono-acetylated and H3 penta-acetylated nucleosome substrates.

After washing, membanes were incubated with an HRP-linked secondary antibody (either anti-Rabbit IgG, Cell signaling #7074S; or anti-Mouse IgG, Cell signaling #7076S) for 1 h at room temperature, then washed. Membranes were then treated with ECL substrate reagent (Bio-Rad, #170-5061), and visualized by G:BOX mini gel imager (Syngene). The bands on the membrane were then quantified by ImageJ (Download from imagej.nih.gov/ij/). All intensity values were divided by the intensity value at t=0 to get relative intensity, and then fit to a single-phase exponential decay curve with constrain Y0=1, Plateau=0 (GraphPad Prism 10). Each plotted point represents at least 2 replicate measurements from the same assay. The kinetic parameter V/[E] was calculated, log10 transformed and plotted as heatmaps using GraphPad Prism 10.

Demethylase kinetics

All demethylase assays and analyses were performed as previously reported.34 Three different 185 bp asymmetric nucleosomes with the following modifications (tail modification #1/tail modification #2) were

prepared: 1) H3K4me2/H3unmodified, 2) H3K4me2/H3K14ac, and 3) H3K4me2-K14ac/H3unmodified.The nucleosomes at 100 nM were mixed with LSD1-CoREST1 (LC) at 180 nM in a reaction buffer containing 50 mM HEPES at pH 7.5, 7 mM KCl, 2.1% glycerol, and 0.2 mg/mL BSA at 25°C. At 0 min, 10 min, 30 min, and 60 min timepoints, 18 µL samples were mixed with 12 µL 4X SDS sample loading buffer, followed by heating at 95°C for 1 min. Then, the samples were resolved by SDS PAGE using 4-20% gradient precast Tris-glycine gels (Thermo Fisher Scientific), transferred to nitrocellulose membranes (iBlot, Thermo Fisher Scientific), blocked with a 1X TBST buffer supplemented with 5% BSA, washed with 1X TBST, and blotted with anti-H3K4me2 (1:2000 dilution, Abcam, cat ab32356) and anti-H3 antibodies (1:2000 dilution, Abcam, cat ab1791). After washing the membranes with 1X TBST, secondary antibodies were added and incubated for 1 hour at room temperature (anti-rabbit IgG HRP-linked antibody, 1:1000 dilution, Cell Signaling Technology, cat 7074S). After the incubation, the membranes were washed with 1X TBST. Each membrane was visualized by ECL (Clarity, Bio-Rad) using a gel imager (G:Box mini, SynGene) and the Genesys (v1.8.5.0) software. The density of each anti-H3K4me2 and anti-H3 band was quantified by ImageJ (NIH), the data were normalized by total H3 at each time point. The relative H3K4me2 intensities (normalized by total H3, and relative to T0) at each time point were fitted into an exponential decay function using GraphPad Prism 9 with the constraints of Y0 at 1 and plateau at 0. The extrapolated rates were converted to V/[E], min-1. All measurements were done in 4 replicates, and One-way ANOVA analysis with Dunnett's multiple comparisons test, with a single pooled variance was employed to compare statistical differences in LC complexes.

Electrophoretic mobility shift assays

Modified 185 bp nucleosomes were thawed on ice and serially diluted in nucleosome storage buffer (10 mM Tris, pH 7.5 at 25 °C, 25 mM NaCl, 1 mM DTT, 20% glycerol) on ice. A solution of sfGFP-RFTS protein in RFTS storage buffer (50 mM Tris, pH 7.5 at 25 °C, 300 mM NaCl, 0.5 mM TCEP, 10% glycerol) was thawed on ice. These were combined in EMSA buffer (15 mM Tris, pH 7.5 at 25 °C, 105 mM NaCl, 0.7 mM DTT, 0.3 mg/mL BSA, 4% glycerol final concentrations) to final concentrations of 20 nM sfGFP-RFTS and 0-160 nM nucleosome, and incubated at 4 °C for 1 h or 24 hours. After incubation, samples were mixed with native sample loading buffer (2.5 x TBE, 50% glycerol), loaded on native 4-20% TBE gels. Gels were run in 0.5 x TBE for 2 hours at 100 V, then sfGFP fluorescence was visualized (Typhoon 5, ex: 488 nm, em: 532 nm). After fluorescent visualization gels were stained with ethidium bromide (30 minutes), rinsed, and DNA was visualized. This titration and visualization was repeated with the concentration of nucleosome fixed at 20 nM, and the concentration of sfGFP-RFTS varied from 0-160 nM. The fraction bound was quantified in ImageJ, and data was fit using GraphPad Prism 10.2.2 (Nonlinear, one site specific binding).

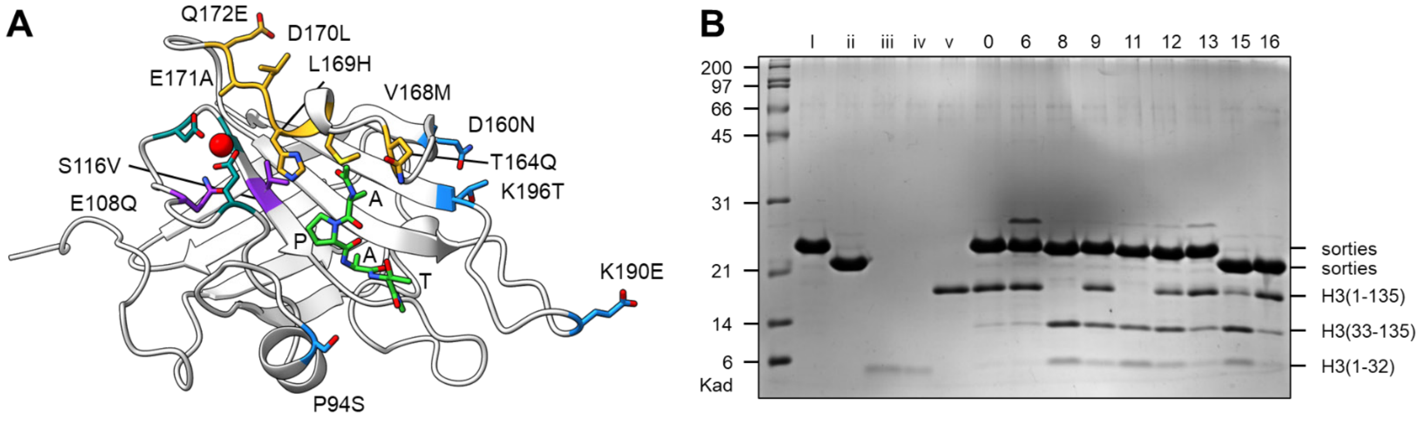

C

#	P94	D160	T164	D165	K190	K196	cleavage yield
0	-	-	Q	-	-	-	**4%**
6	S	N	-	A	-	-	**7%**
8	-	N	Q	-	E	T	**79%**
9	S	N	Q	-	-	T	**29%**
11	S	N	Q	-	E	T	**82%**
12	S	N	-	A	-	T	**39%**
13	S	N	Q	A	E	T	**16%**
15	R	N	Q	-	E	T	**55%**
16	R	N	Q	-	-	T	**20%**

Figure S1. Cleavage of purified, recombinant histone H3 by sortase mutants. (**A**) Sites of mutations found in W11 sortase, with origin indicated by color: F40 (yellow); enhanced sortase (blue); FireProt (purple). Calcium (red) binding residues are depicted in dark green, and bound substrate is depicted in light green. Mutations introduced in pymol (PDBID: 2KID), followed by Rosetta energy minimization. (**B**) SDS-PAGE of sortase cleavage reaction with recombinant, unmodified histone H3: i & ii – sortase mutant standards; iii & iv – H3 tail peptide standards; v – H3 protein standard; 0 – F40 sortase; 6-16 – F40-derived mutants. (**C**) Specific enhanced sortase mutations introduced to F40 in mutants W6 through W16 and resultant H3 tail cleavage yields.

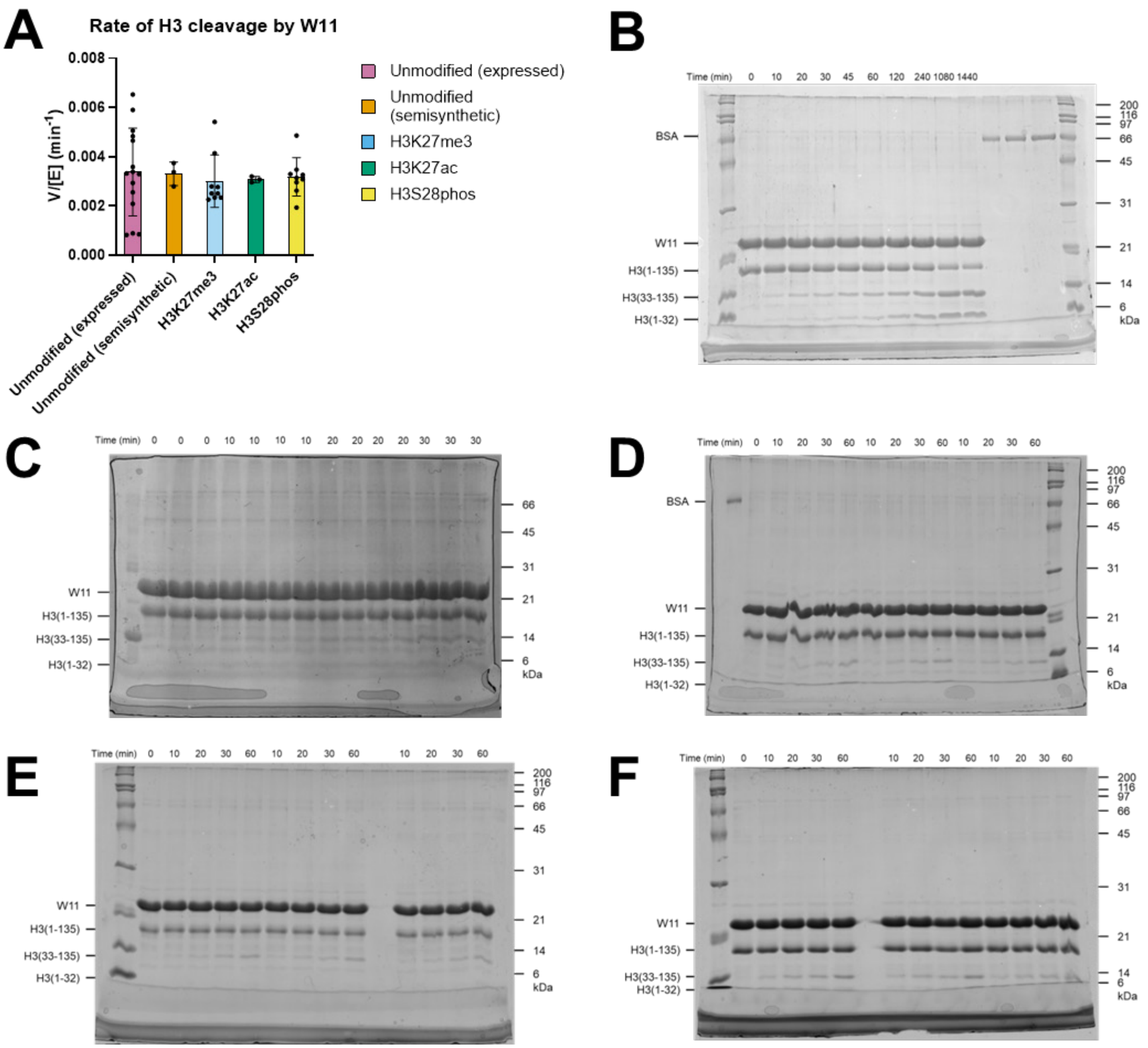

Figure S2. Cleavage of semisynthetic, modified histone H3 by W11. (**A**) Rates of H3 cleavage by W11 sortase in the presence of post-translational modifications near the sortase motif (A29-G33). Rates were determined by densitometry (ImageJ) from SDS-PAGE analysis of starting material, H3(1-135), disappearance and product, H3(33-135), formation over time (unmodified expressed n=15; unmodified semisynthetic n=3; H3K27me3 n=9; H3K27ac n=3; H3S28phos n=9). Data was fitted to a non-linear one-phase decay using GraphPad Prism 10.2.3. Error bars depict standard deviation. (**B-F**) Representative SDS-PAGE gels used for evaluating W11 sortase cleavage rate: (**B**) unmodified, heterologously expressed H3; (**C**) unmodified, semisynthetic H3; (**D**) semisynthetic H3K27me3; (**E**) semisynthetic H3K27ac; (**F**) semisynthetic H3S28phos. Reactions run with 30 μM H3, 100 μM sortase, 1 mM GGG peptide, 20 mM PIPES (pH 7), 1 mM CaCl2, 1 mM DTT.

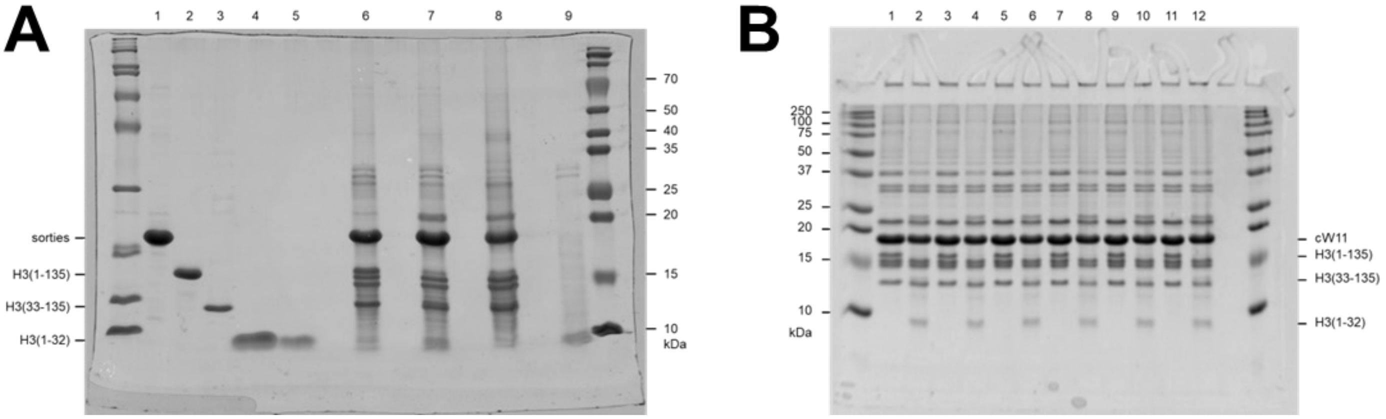

Figure S3. SDS-PAGE analysis of sortase W11 & cW11 reactions in nuclear acid extracts. (**A**) W11 reaction with standards: lane 1 – W11; lane 2 – H3(1-135); lane 3 – H3(33-135); lane 4 & 5 – H3(1-32) standard; lane 6 – 0 hr acid extract reaction; lane 7 – 16 hr acid extract reaction; lane 8 – TCA pelleted protein from 16 hr acid extract reaction; lane 9 – buffer exchanged supernatant from TCA precipitation. (**B**) Representative replicate acid extract reactions with cW11: lane 1 – 0 hr acid extract reaction replicate 1; lane 2 – 16 hr acid extract reaction replicate 1; lane 3 – 0 hr acid extract reaction replicate 2; lane 4 – 16 hr acid extract reaction replicate 2; lane 5 – 0 hr acid extract reaction replicate 3; lane 6 – 16 hr acid extract reaction replicate 3; lane 7 – 0 hr acid extract reaction replicate 4; lane 8 – 16 hr acid extract reaction replicate 4; lane 9 – 0 hr acid extract reaction replicate 5; lane 10 – 16 hr acid extract reaction replicate 5; lane 11 – 0 hr acid extract reaction replicate 6; lane 12 – 16 hr acid extract reaction replicate 6. Lanes 1 & 2 reprinted from main text figure 1.

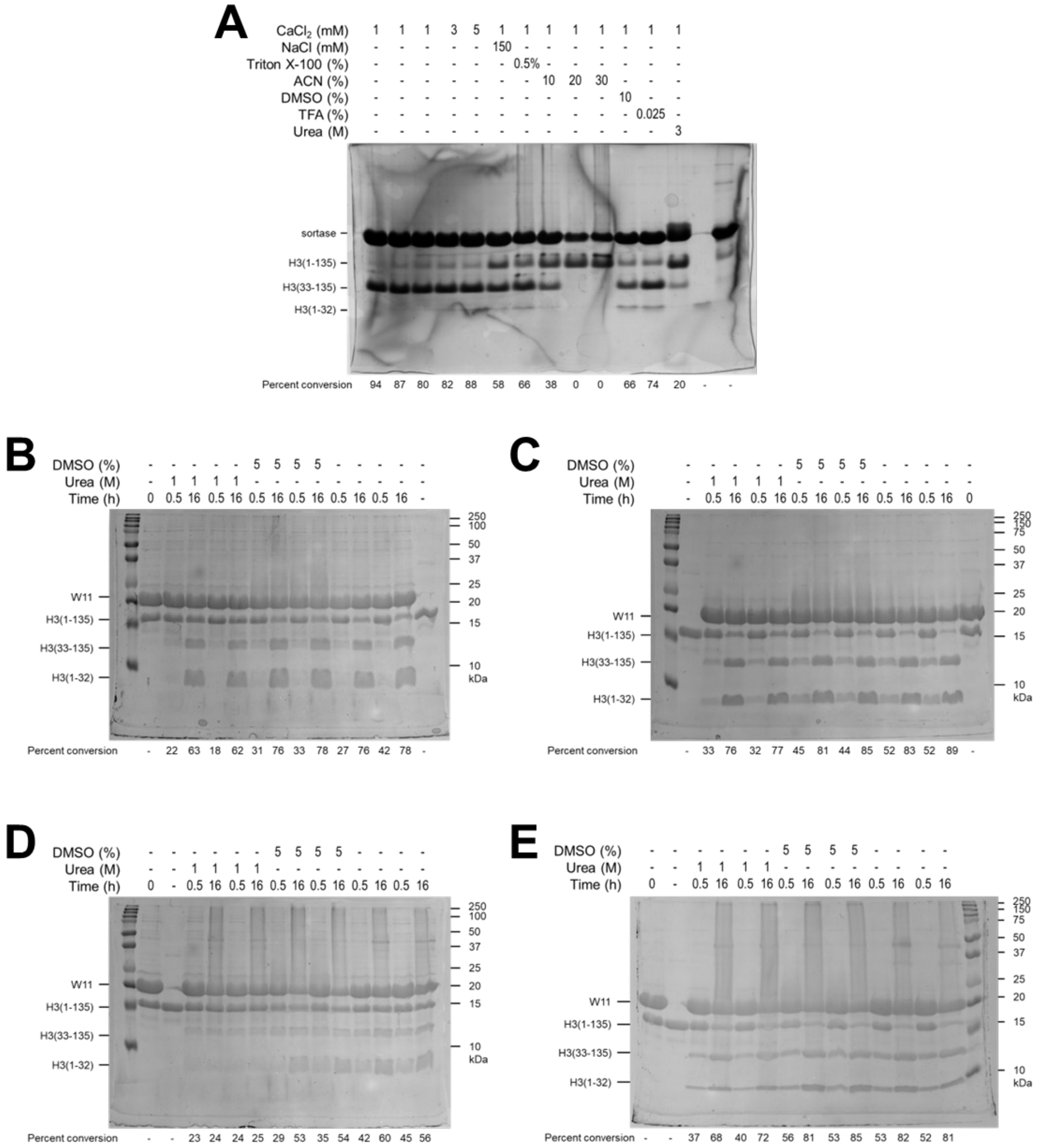

Figure S4. Sortase W11 mutant activity in the presence of co-solvent, detergent or chaotrope. (**A**) Endpoint SDS-PAGE analysis of 25 °C sortase W11 reaction run with variant buffer conditions including increased salt, added detergent, organic co-solvent, or chaotrope. (**B** & **C**) Endpoint SDS-PAGE analysis of duplicate 25 °C sortase reaction with W11 (**B**) or FireProt mutant W11(E108Q, S116V) (**C**). (**D** & **E**) Endpoint SDS-PAGE analysis of duplicate 37 °C sortase reaction with W11 (**D**) or FireProt mutant W11(E108Q, S116V) (**E**). In all cases cleavage of H3 was evaluated by densitometry (ImageJ), and percent conversion was calculated as the sum of H3(33-135) and H3(1-32) over the cumulative intensity of all H3-derived bands.

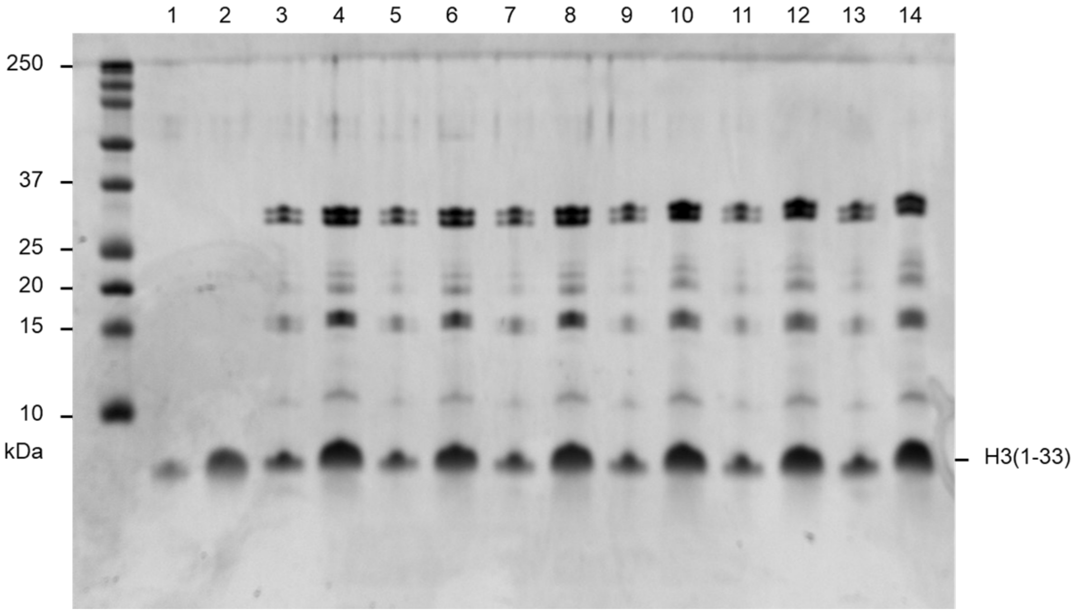

Figure S5. Histone tails isolated by trichloroacetic acid precipitation of reaction protein components. Representative histone tails isolated by TCA precipitation from the sortase reaction conducted in nuclear acid extracts. Samples are derived from reactions shown in figure S3b, and a portion (lanes 1-3) of this gel is re-printed in main figure 1. Representative TCA products from replicate acid extract reactions with cW11: lane 1 & 2 – H3(1-32) peptide standards for quantification; lane 3 & 4 – replicate 1 product; lane 5 & 6 – replicate 2 product; lane 7 & 8 – replicate 3 product; lane 9 & 10 – replicate 4 product; lane 11 & 12 – replicate 5 product; lane 13 & 14 – replicate 6 product.

	Middle-down		Bottom-up			Middle-down		Bottom-up	
PTM	DMSO & Corin H3.1 & H3.3	DMSO & MS275 H3.1 & H3.3	DMSO & Corin H3.1 & H3.3	DMSO & MS275 H3.1 & H3.3	PTM	DMSO & Corin H3.1 & H3.3	DMSO & MS275 H3.1 & H3.3	DMSO & Corin H3.1 & H3.3	DMSO & MS275 H3.1 & H3.3
unmodified	0.02%	0.10%	0.00%	0.00%	K18me1	0.08%	0.10%	0.01%	0.00%
R2me1	0.00%	0.00%	0.00%	0.00%	K18me2	0.05%	0.00%	1.34%	1.76%
R2me2	0.00%	0.00%	0.00%	0.00%	K18me3	0.00%	0.00%	0.04%	0.05%
T3phos	0.00%	0.00%	0.00%	0.00%	K18ac	1.31%	1.55%	0.00%	0.00%
K4me1	0.21%	0.05%	24.06%	24.24%	K18prop	0.23%	0.15%	0.00%	0.00%
K4me2	0.00%	0.00%	0.00%	0.00%	T22phos	11.18%	14.69%	0.00%	0.00%
K4me3	0.00%	0.00%	0.00%	0.00%	K23me1	0.34%	0.97%	0.00%	0.00%
K4ac	0.00%	0.00%	0.00%	0.00%	K23me2	0.41%	0.37%	0.00%	0.00%
K4prop	0.00%	0.00%	0.00%	0.00%	K23me3	0.00%	0.05%	0.00%	0.00%
T6phos	0.00%	0.00%	0.00%	0.00%	K23ac	66.14%	66.15%	54.89%	57.79%
R8me1	0.20%	0.00%	0.00%	0.00%	K23prop	4.07%	3.99%	0.00%	0.00%
R8me2	0.12%	0.24%	0.00%	0.00%	R26me1	7.19%	5.83%	0.00%	0.00%
K9me1	12.84%	11.10%	34.21%	30.82%	R26me2	0.94%	0.72%	0.00%	0.00%
K9me2	32.04%	32.33%	17.41%	15.88%	H3.1_K27me1	7.57%	7.94%	23.45%	23.45%
K9me3	6.40%	5.91%	8.81%	8.04%	H3.1_K27me2	67.70%	68.95%	44.06%	44.12%
K9ac	6.48%	7.63%	3.54%	3.54%	H3.1_K27me3	0.15%	0.13%	14.58%	14.88%
K9prop	0.84%	0.81%	0.00%	0.00%	H3.1_K27ac	1.56%	0.90%	0.03%	0.03%
S10phos	0.00%	0.05%	0.00%	0.00%	H3.1_K27prop	0.20%	0.14%	0.00%	0.00%
T11phos	0.16%	0.11%	0.00%	0.00%	H3.3_K27me1	0.00%	0.00%	31.08%	31.16%
K14me1	0.47%	0.20%	0.00%	0.00%	H3.3_K27me2	9.01%	47.55%	35.06%	34.61%
K14me2	0.11%	0.15%	0.00%	0.00%	H3.3_K27me3	0.00%	0.00%	14.64%	14.31%
K14me3	0.00%	0.13%	0.00%	0.00%	H3.3_K27ac	0.00%	0.00%	0.00%	0.00%
K14ac	71.65%	70.81%	31.28%	31.15%	H3.3_K27prop	2.90%	0.00%	0.00%	0.00%
K14prop	1.61%	2.50%	0.00%	0.00%	S28phos	0.24%	0.16%	0.00%	0.00%
R17me1	0.06%	0.28%	0.00%	0.00%	S31phos	0.00%	0.00%	0.00%	0.00%
R17me2	0.00%	0.04%	0.00%	0.00%	T32phos	0.00%	0.00%	0.00%	0.00%
	Pearson correlation								
middle-down v. bottom-up			75.01%	80.17%					

Table S1. Abundance of individual H3 post-translational modifications in bottom-up and middle-down proteomics data.

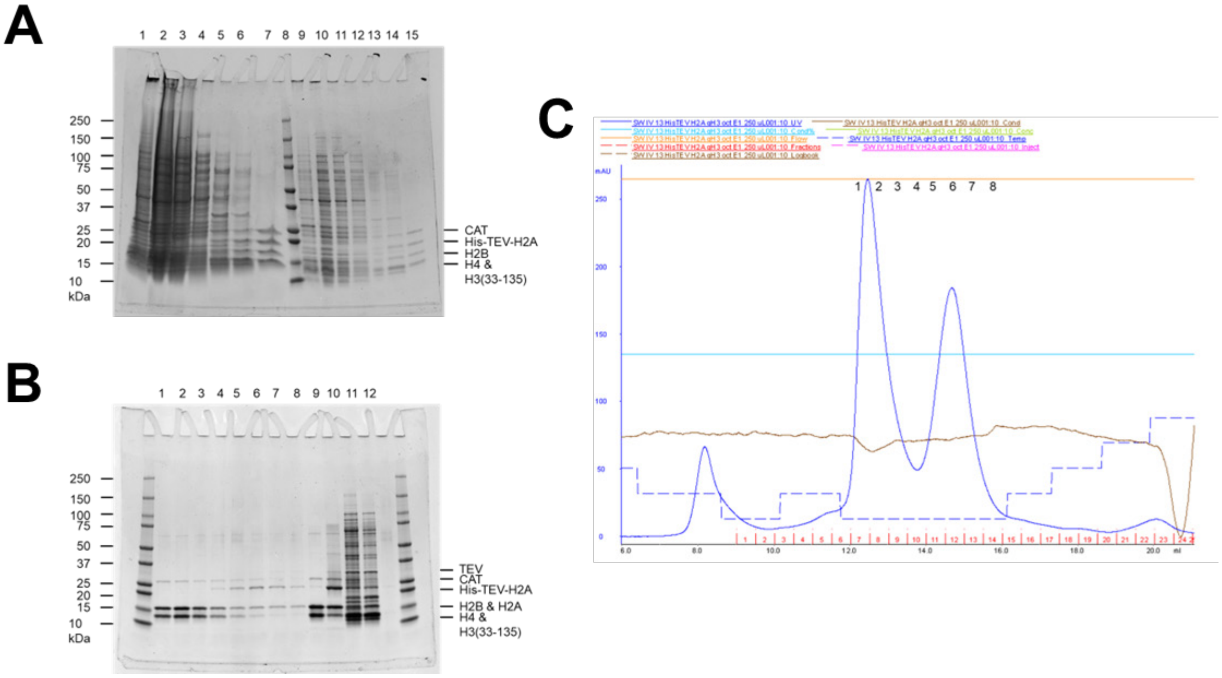

Figure S6. Characterization of H3(33-135) octamer overexpression and purification. (**A**) SDS-PAGE evaluation of fractions from IMAC purification of co-overexpressed His-TEV-H2A, H2B, H3(33-135) and H4. Lanes: 1 – lysate pellet; 2 – lysate supernatant; 3 – IMAC column flow through; 4 – lysis buffer wash; 5 – 20 mM imidazole wash 1; 6 – 20 mM imidazole wash 2; 7 – 200 mM imidazole elution; 8 – ladder; 9 – lysate pellet; 10 – lysate supernatant; 11 – IMAC column flow through; 12 – lysis buffer wash; 13 – 20 mM imidazole wash 1; 14 – 20 mM imidazole wash 2; 15 – 200 mM imidazole elution. CAT – chloramphenicol acetyltransferase. (**B**) SDS-PAGE evaluation of TEV cleavage of His-TEV-H2A octamer, and superdex200 fractions from post-TEV purification. Lanes: 1-8 – sequential superdex200 fractions (see **C**); 9 – TEV cleavage endpoint; 10 – TEV cleavage starting point; 11 – IMAC 20 mM imidazole wash 1; 12 – IMAC 20 mM imidazole wash 2. (**C**) Superdex200 chromatogram from purification following TEV cleavage of His-TEV-H2A octamer. Numbering (top) reflects corresponds to gel lanes in figure S6B.

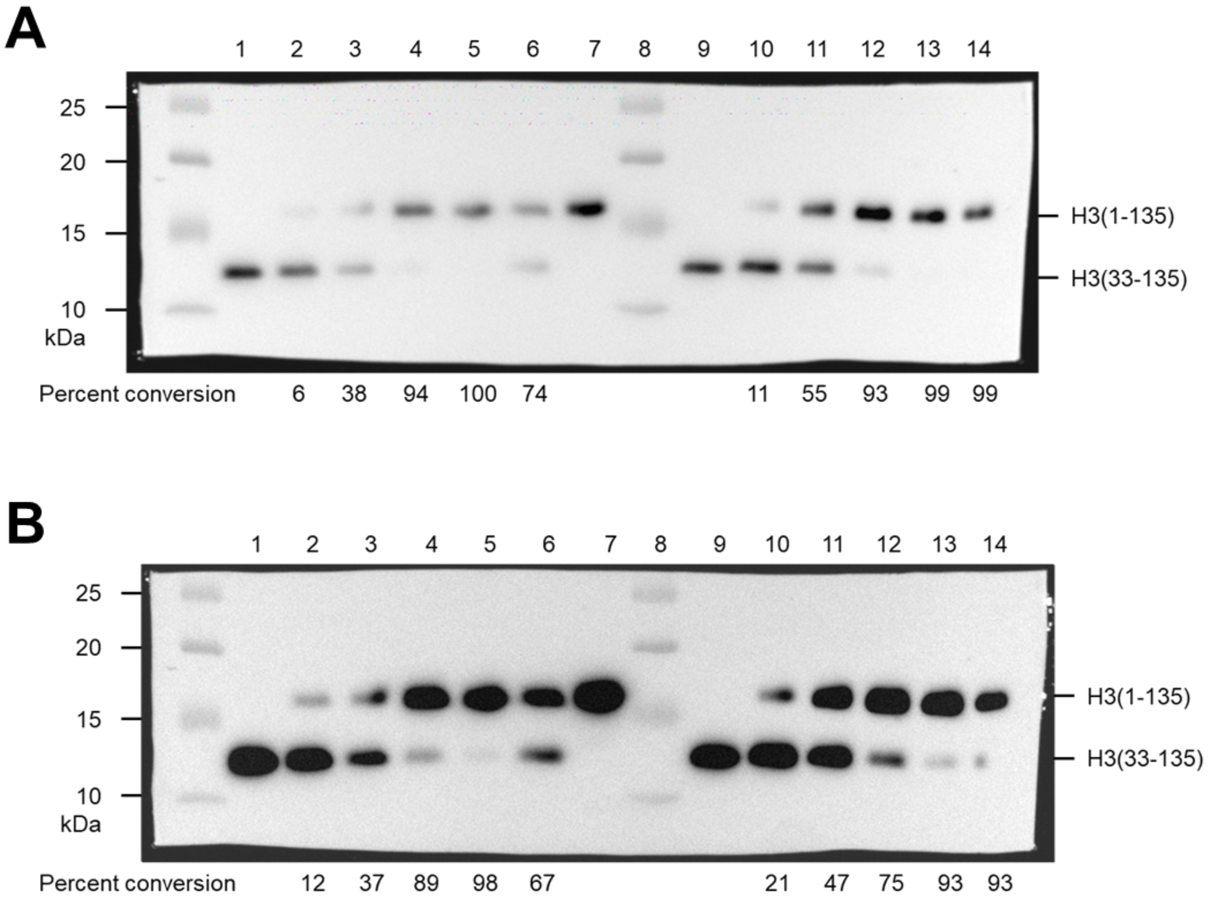

Figure S7. Western blot analysis of cW11 nucleosome ligation. (**A**) Anti-H3 western blot (1 minute exposure) of cW11 sortase ligation time-course replicates and DEAE fractions from an un-optimized gradient. Lanes: 1 – H3(33-135) standard; 2 – 0.5 hours; 3 – 1 hour; 4 – 4 hours; 5 – DEAE first peak; 6 – DEAE second peak; 7 – H3(1-135) standard; 8 – ladder; 9 – H3(33-135) standard; 10 – 0.5 hours; 11 – 1 hour; 12 – 4 hours; 13 – DEAE first peak; 14 – DEAE second peak. (**B**) Long exposure (10 minutes) of western blot from **A**. Percent conversion was assessed by densitometry using ImageJ.

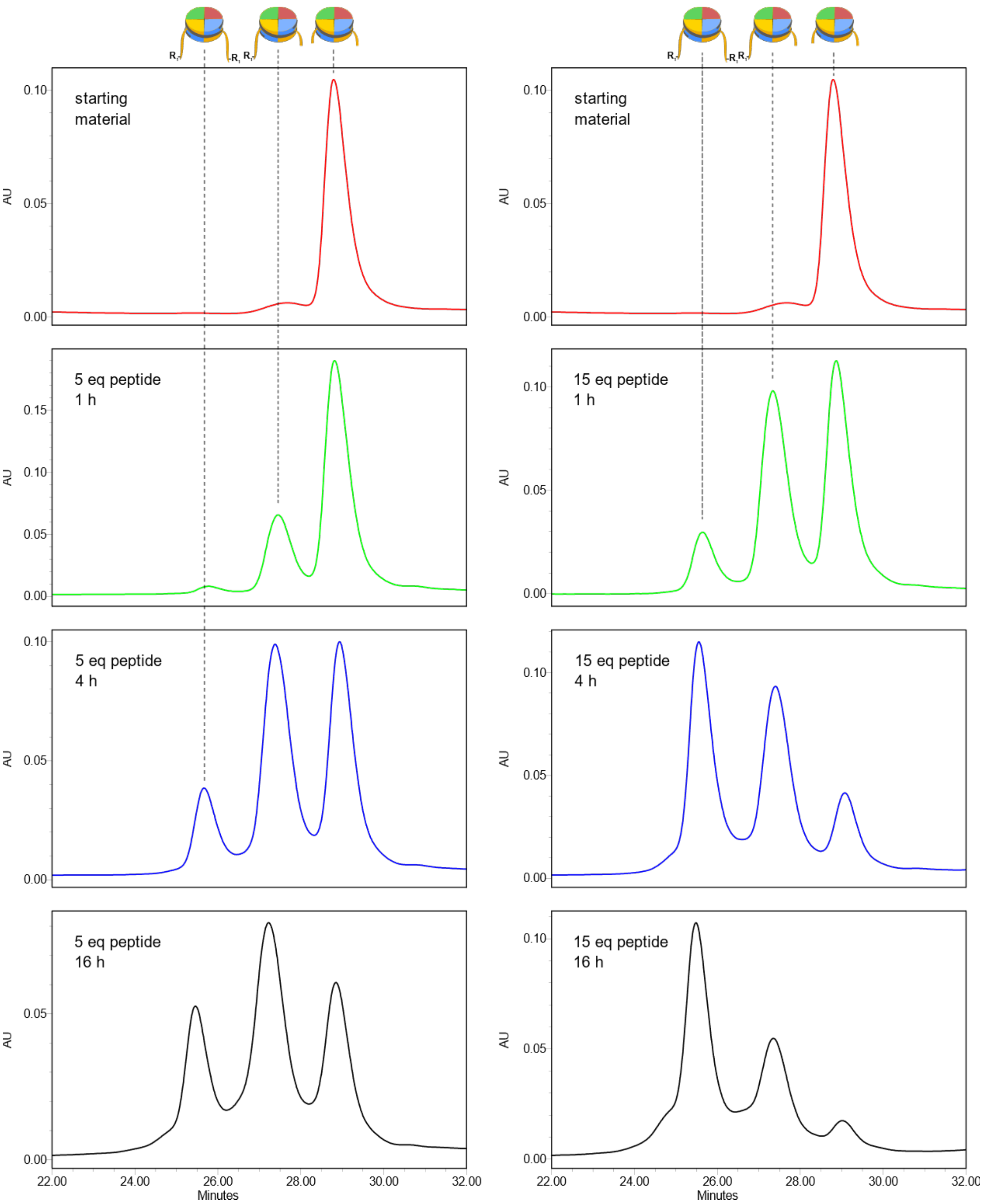

Figure S8. cW11 nucleosome ligation time course chromatograms. Change in product distribution over the course of the cW11 sortase ligation using either 5 equivalents of H3 tail peptide (**left**) or 15 equivalents (**right**). Peaks correspond to nucleosome with two copies of full length H3 (**left**), one copy of full length H3 and one copy of N-terminally truncated H3 (aa33-135) (**middle**), and starting material nucleosome with two copies of truncated H3 (aa33-135) (**right**).

Figure S9. Mass spectrometric characterization of cW11 nucleosome ligation products. Raw spectra deconvoluted with UniDec.[19]

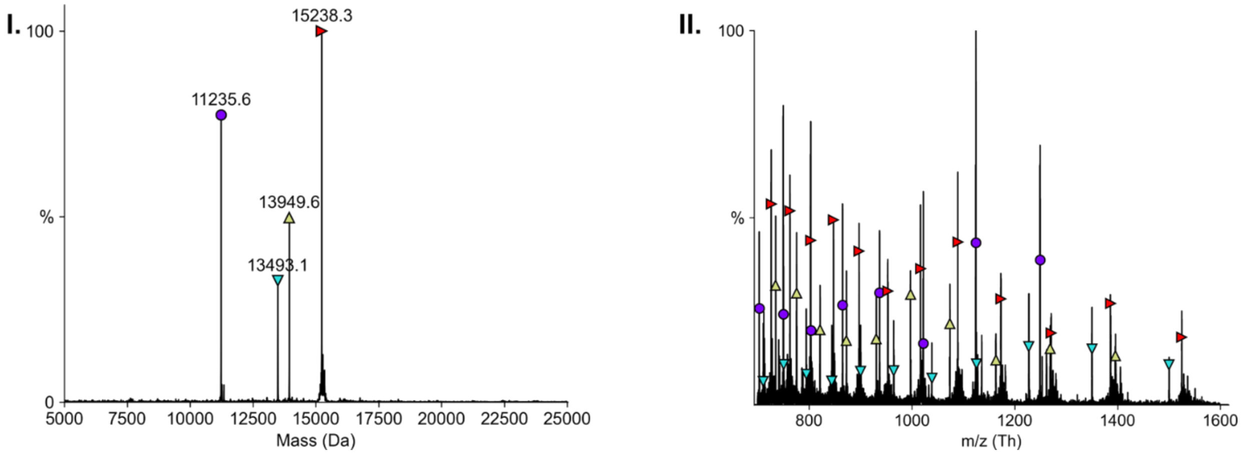

A. 147 bp unmodified nucleosome. (**I**) Deconvoluted mass spectrum: H4 (purple circle) calculated mass 11236.15 Da, found: 11235.6 Da; H2B (teal downward pointed triangle) calculated mass 13493.68 Da, found: 13493.2 Da; H2A (green upward pointed triangle) calculated mass 13950.2 Da, found: 13949.6 Da; unmodified H3 (red rightward pointed triangle) calculated mass 15238.61 Da, found: 15238.2 Da. (**II**) Raw mass spectrum: H4 (purple circle); H2B (teal downward pointed triangle); H2A (green upward pointed triangle; H3 (red rightward pointed triangle).

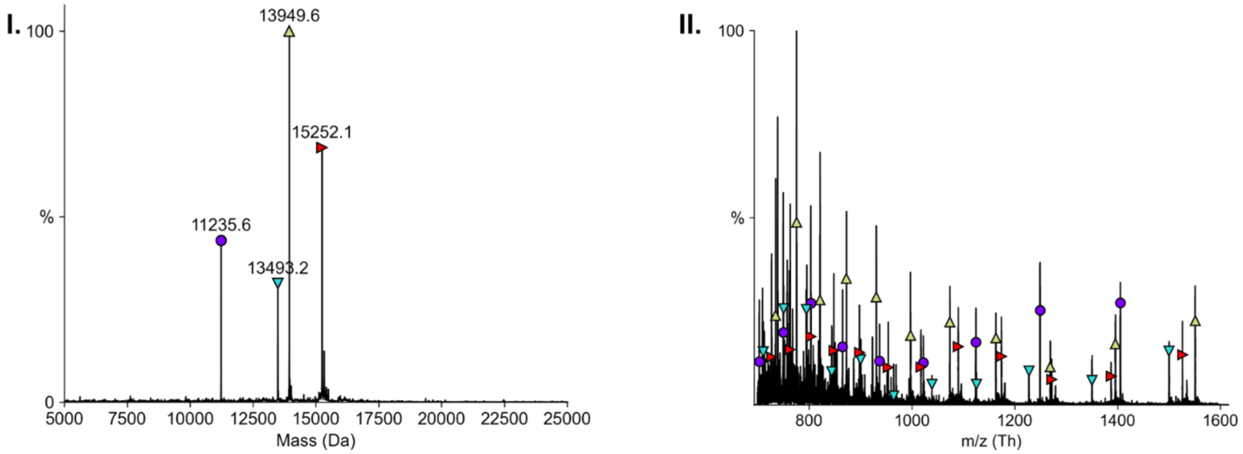

B. 147 bp H3K4-monomethyl nucleosome. (**I**) Deconvoluted mass spectrum: H4 (purple circle) calculated mass 11236.15 Da, found: 11235.6 Da; H2B (teal downward pointed triangle) calculated mass 13493.68 Da, found: 13493.2 Da; H2A (green upward pointed triangle) calculated mass 13950.2 Da, found: 13949.6 Da; H3K4me1 (red rightward pointed triangle) calculated mass 15251.63 Da, found: 15252.1 Da. (**II**) Raw mass spectrum: H4 (purple circle); H2B (teal downward pointed triangle); H2A (green upward pointed triangle; H3K4me1 (red rightward pointed triangle).

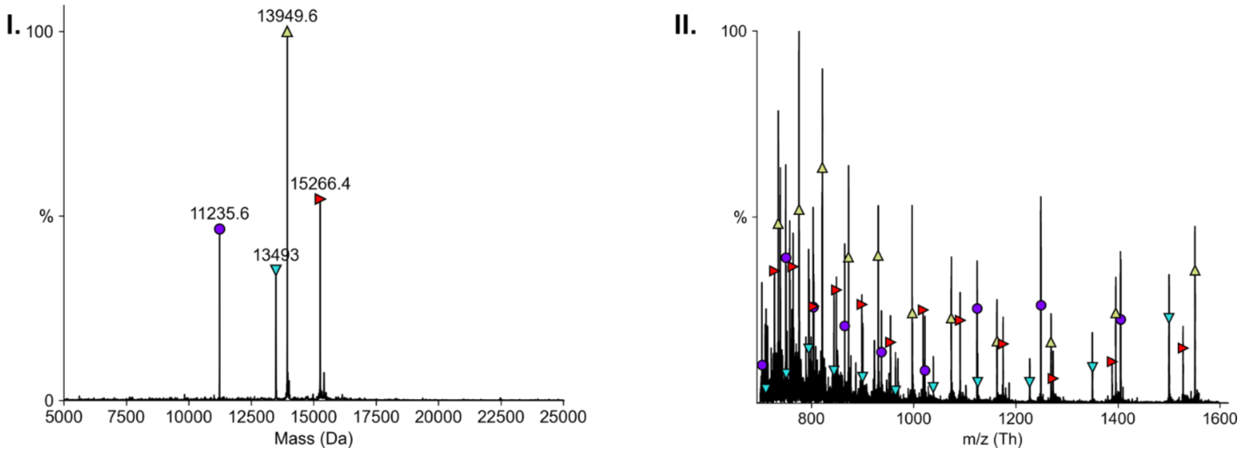

C. 147 bp H3K4-dimethyl I nucleosome. (**I**) Deconvoluted mass spectrum: H4 (purple circle) calculated mass 11236.15 Da, found: 11235.4 Da; H2B (teal downward pointed triangle) calculated mass 13493.68 Da, found: 13493.0 Da; H2A (green upward pointed triangle) calculated mass 13950.2 Da, found: 13949.5 Da; H3K4me2 (red rightward pointed triangle) calculated mass 15266.66 Da, found: 15266.4 Da. (**II**) Raw mass spectrum: H4 (purple circle); H2B (teal downward pointed triangle); H2A (green upward pointed triangle; H3K4me2 (red rightward pointed triangle).

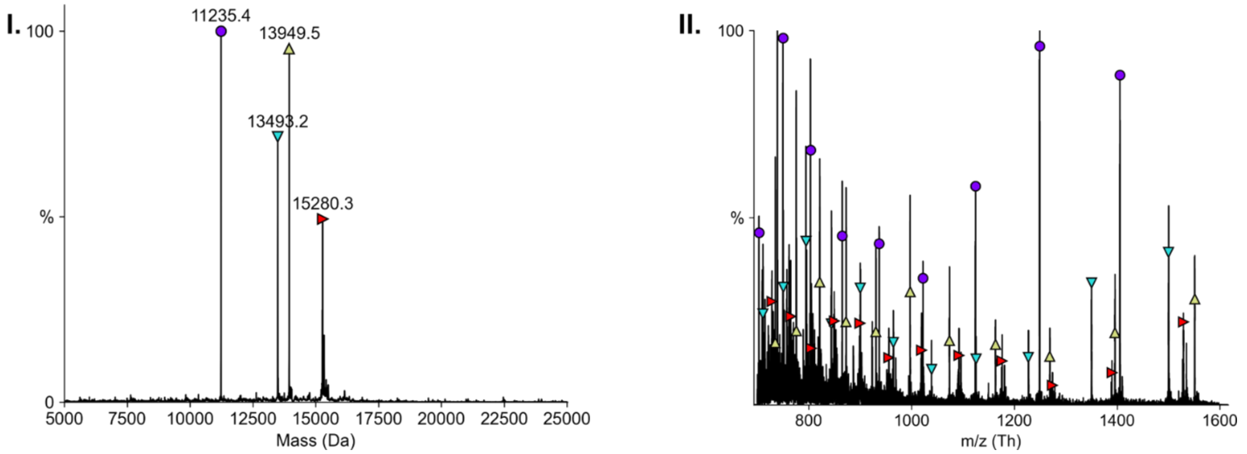

D. 147 bp H3K4-trimethyl nucleosome. (**I**) Deconvoluted mass spectrum: H4 (purple circle) calculated mass 11236.15 Da, found: 11235.4 Da; H2B (teal downward pointed triangle) calculated mass 13493.68 Da, found: 13493.0 Da; H2A (green upward pointed triangle) calculated mass 13950.2 Da, found: 13949.5 Da; H3K4me3 (red rightward pointed triangle) calculated mass 15308.70 Da, found: 15308.2 Da. (**II**) Raw mass spectrum: H4 (purple circle); H2B (teal downward pointed triangle); H2A (green upward pointed triangle; H3K4me3 (red rightward pointed triangle).

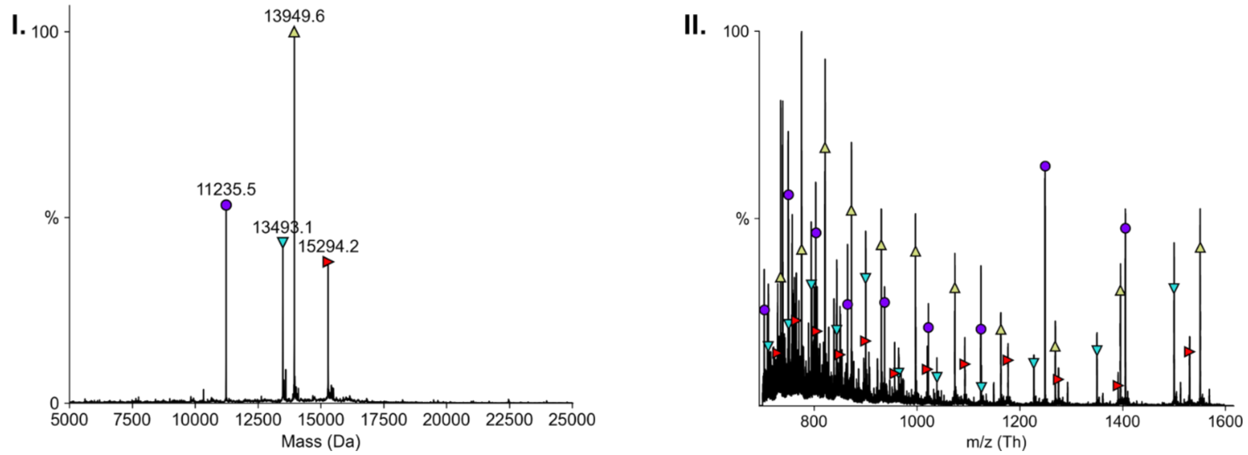

E. 147 bp H3K9-propionyl nucleosome. (**I**) Deconvoluted mass spectrum: H4 (purple circle) calculated mass 11236.15 Da, found: 11235.5 Da; H2B (teal downward pointed triangle) calculated mass 13493.68 Da, found: 13493.1 Da; H2A (green upward pointed triangle) calculated mass 13950.2 Da, found: 13949.6 Da; H3K9pr (red rightward pointed triangle) calculated mass 15294.67 Da, found: 15294.2 Da. (**II**) Raw mass spectrum: H4 (purple circle); H2B (teal downward pointed triangle); H2A (green upward pointed triangle; H3K9pr (red rightward pointed triangle).

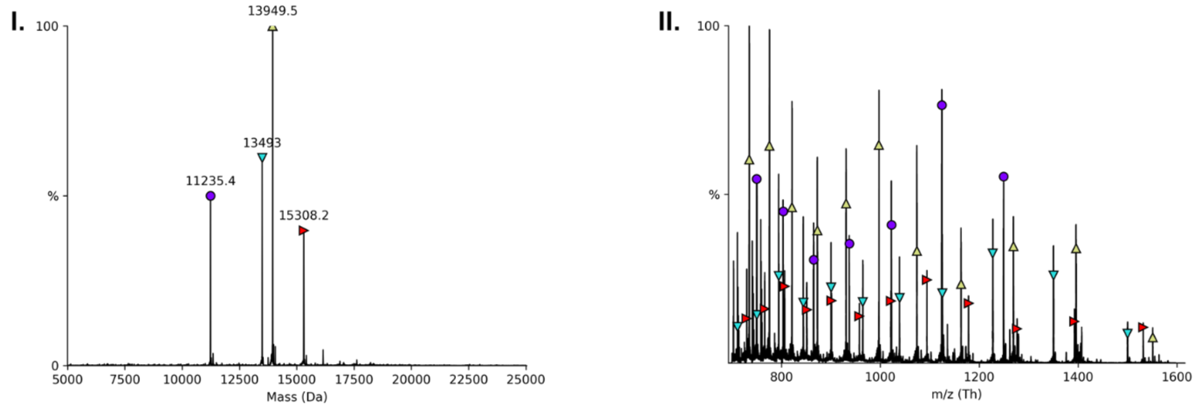

F. 147 bp H3K9-butyryl nucleosome. (**I**) Deconvoluted mass spectrum: H4 (purple circle) calculated mass 11236.15 Da, found: 11235.4 Da; H2B (teal downward pointed triangle) calculated mass 13493.68 Da, found: 13493.0 Da; H2A (green upward pointed triangle) calculated mass 13950.2 Da, found: 13949.5 Da; H3K9bu (red rightward pointed triangle) calculated mass 15308.70 Da, found: 15308.2 Da. (**II**) Raw mass spectrum: H4 (purple circle); H2B (teal downward pointed triangle); H2A (green upward pointed triangle; H3K9bu (red rightward pointed triangle).

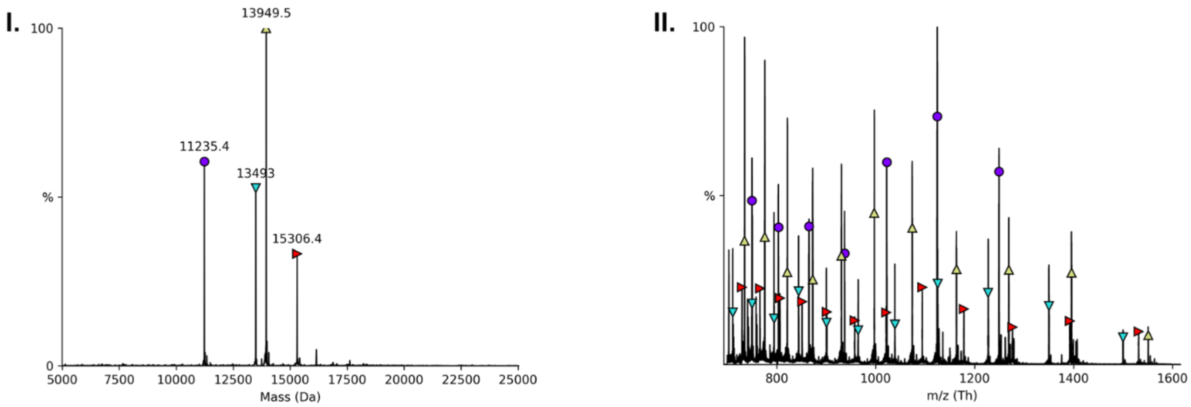

G. 147 bp H3K9-crotonyl nucleosome. (**I**) Deconvoluted mass spectrum: H4 (purple circle) calculated mass 11236.15 Da, found: 11235.4 Da; H2B (teal downward pointed triangle) calculated mass 13493.68 Da, found: 13493.0 Da; H2A (green upward pointed triangle) calculated mass 13950.2 Da, found: 13949.5 Da; H3K9cro (red rightward pointed triangle) calculated mass 15306.68, found: 15306.4. (**II**) Raw mass spectrum: H4 (purple circle); H2B (teal downward pointed triangle); H2A (green upward pointed triangle); H3K9cro (red rightward pointed triangle).

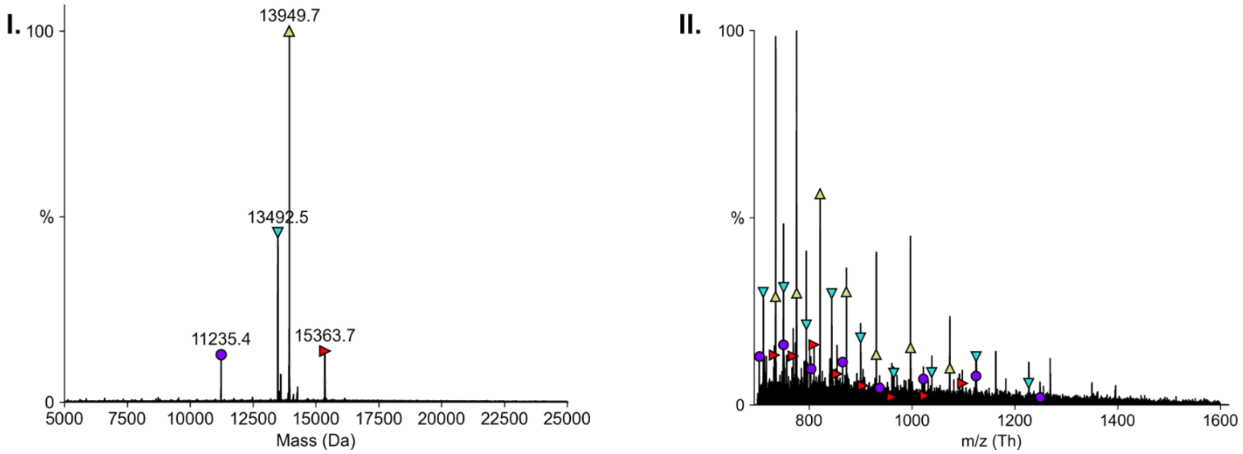

H. 147 bp H3K9-octanoyl nucleosome. (**I**) Deconvoluted mass spectrum: H4 (purple circle) calculated mass 11236.15 Da, found: 11235.2 Da; H2B (teal downward pointed triangle) calculated mass 13493.68 Da, found: 13493.3 Da; H2A (green upward pointed triangle) calculated mass 13950.2 Da, found: 13948.7 Da; H3K9oct (red rightward pointed triangle) calculated mass 15363.80, found: 15364.8. (**II**) Raw mass spectrum: H4 (purple circle); H2B (teal downward pointed triangle); H2A (green upward pointed triangle); H3K9oct (red rightward pointed triangle).

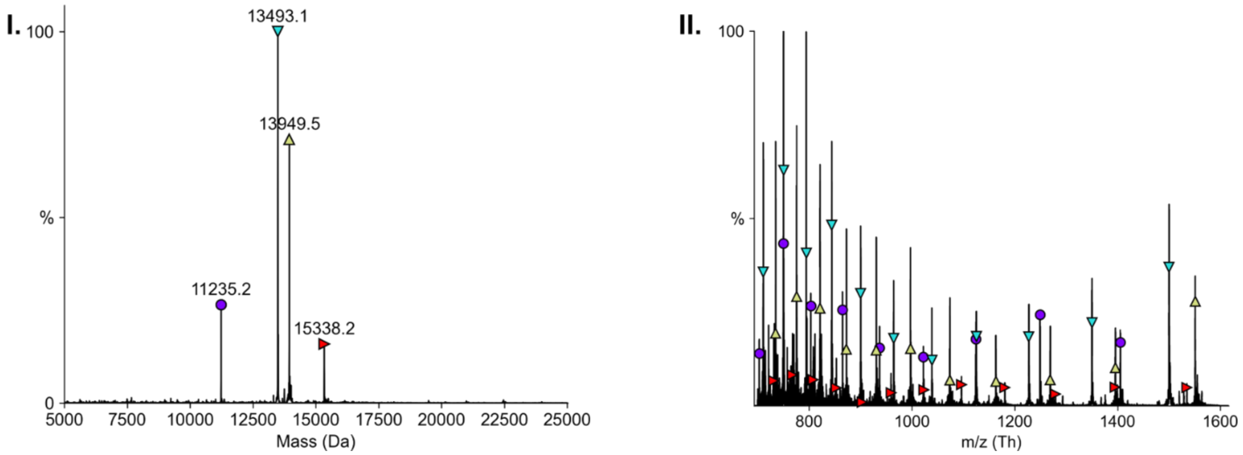

I. 147 bp H3K9-succinyl nucleosome. (**I**) Deconvoluted mass spectrum: H4 (purple circle) calculated mass 11236.15 Da, found: 11234.9 Da; H2B (teal downward pointed triangle) calculated mass 13493.68 Da, found: 13942.6 Da; H2A (green upward pointed triangle) calculated mass 13950.2 Da, found: 13949.7 Da; H3K9suc (red rightward pointed triangle) calculated mass 15337.67, found: 15338.6. (**II**) Raw mass spectrum: H4 (purple circle); H2B (teal downward pointed triangle); H2A (green upward pointed triangle); H3K9suc (red rightward pointed triangle).

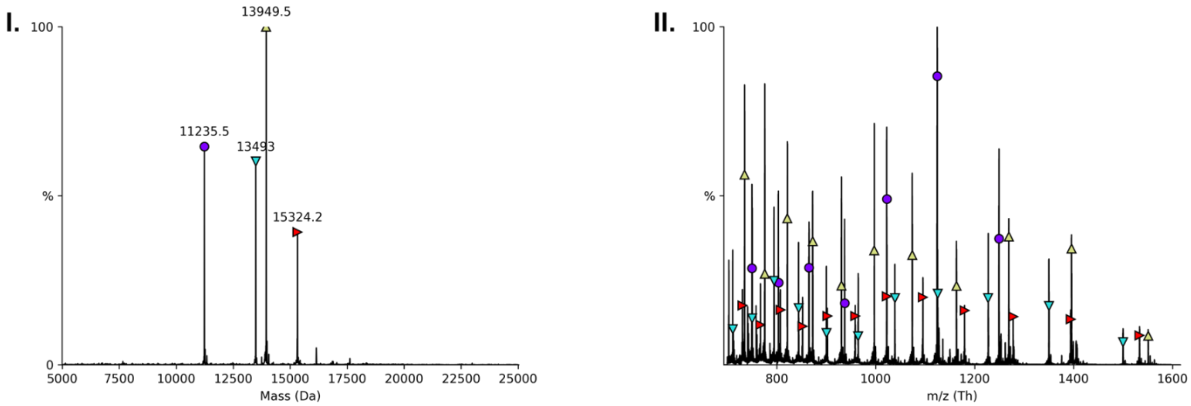

J. 147 bp H3K9-anti-hydroxyisobutyryl nucleosome. (**I**) Deconvoluted mass spectrum: H4 (purple circle) calculated mass 11236.15 Da, found: 11235.5 Da; H2B (teal downward pointed triangle) calculated mass 13493.68 Da, found: 13493.0 Da; H2A (green upward pointed triangle) calculated mass 13950.2 Da, found: 13949.5 Da; H3K9hib (red rightward pointed triangle) calculated mass 15324.70, found: 15324.2. (**II**) Raw mass spectrum: H4 (purple circle); H2B (teal downward pointed triangle); H2A (green upward pointed triangle); H3K9hib (red rightward pointed triangle).

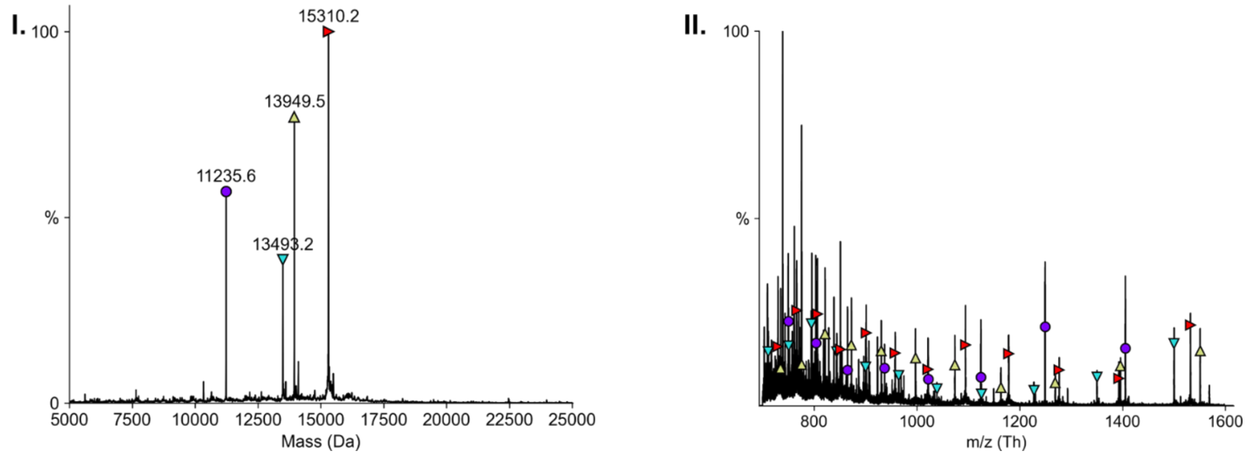

K. 147 bp H3K9-lactyl nucleosome. (**I**) Deconvoluted mass spectrum: H4 (purple circle) calculated mass 11236.15 Da, found: 11235.5 Da; H2B (teal downward pointed triangle) calculated mass 13493.68 Da, found: 13493.0 Da; H2A (green upward pointed triangle) calculated mass 13950.2 Da, found: 13949.5 Da; H3K9lac (red rightward pointed triangle) calculated mass 15310.67, found: 15310.2. (**II**) Raw mass spectrum: H4 (purple circle); H2B (teal downward pointed triangle); H2A (green upward pointed triangle); H3K9lac (red rightward pointed triangle).

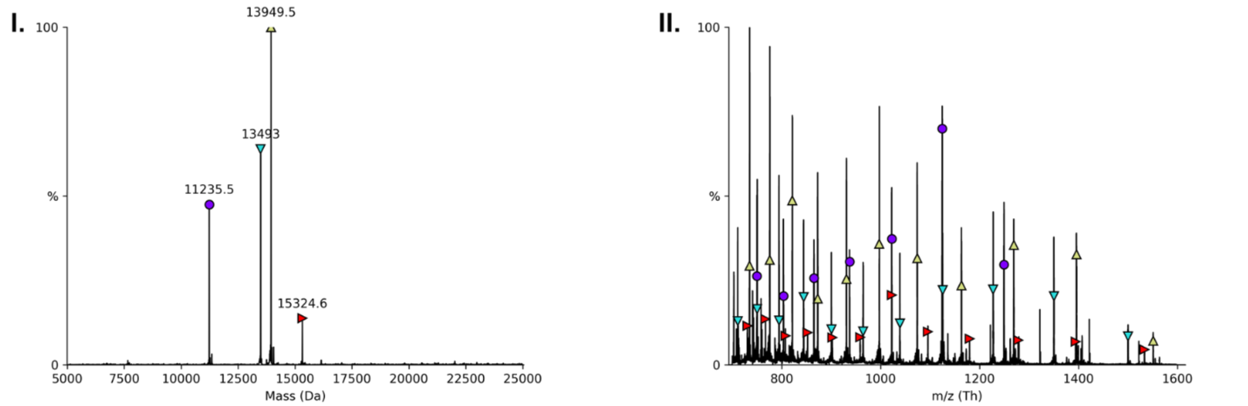

L. 147 bp H3K9-β-hydroxybutyryl nucleosome. (**I**) Deconvoluted mass spectrum: H4 (purple circle) calculated mass 11236.15 Da, found: 11235.5 Da; H2B (teal downward pointed triangle) calculated mass 13493.68 Da, found: 13493.0 Da; H2A (green upward pointed triangle) calculated mass 13950.2 Da, found: 13949.5 Da; H3K9bhb (red rightward pointed triangle) calculated mass 15324.70, found: 15324.6. (**II**) Raw mass spectrum: H4 (purple circle); H2B (teal downward pointed triangle); H2A (green upward pointed triangle); H3K9bhb (red rightward pointed triangle).

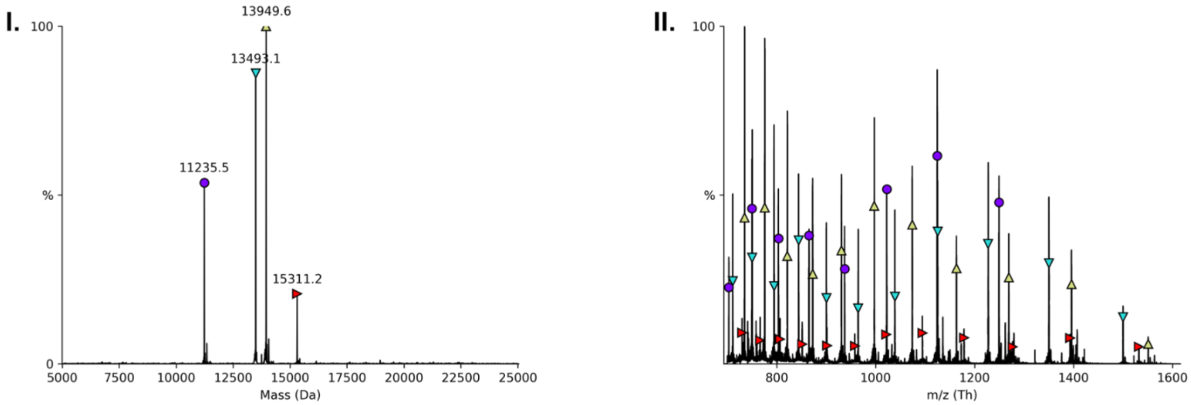

M. 147 bp H3K9-N-methyl thiourea nucleosome. (**I**) Deconvoluted mass spectrum: H4 (purple circle) calculated mass 11236.15 Da, found: 11235.5 Da; H2B (teal downward pointed triangle) calculated mass 13493.68 Da, found: 13493.1 Da; H2A (green upward pointed triangle) calculated mass 13950.2 Da, found: 13949.6 Da; H3K9mtu (red rightward pointed triangle) calculated mass 15311.72, found: 15311.2 Da. (**II**) Raw mass spectrum: H4 (purple circle); H2B (teal downward pointed triangle); H2A (green upward pointed triangle); H3K9mtu (red rightward pointed triangle).

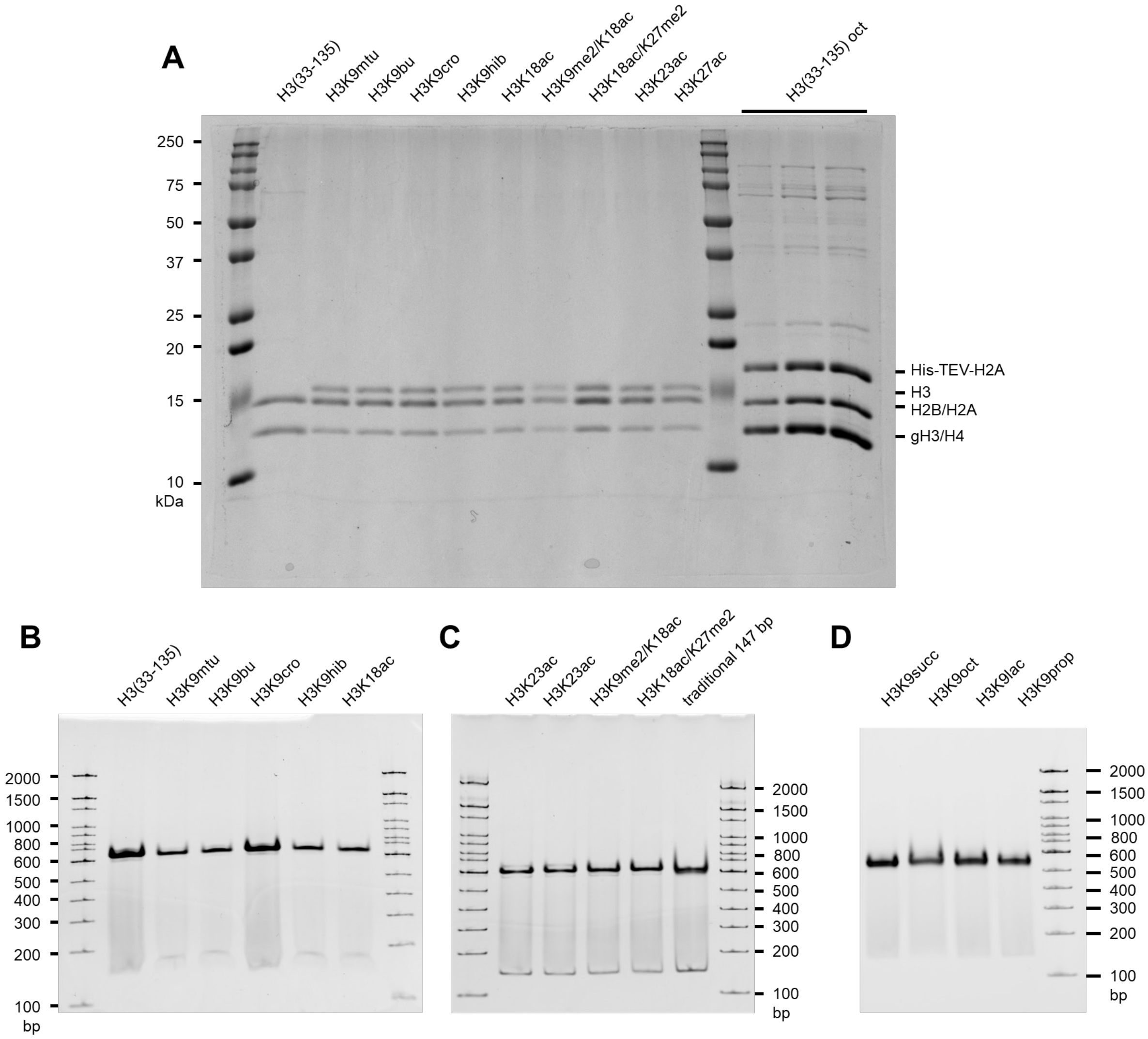

Figure S10. SDS-PAGE and TBE native gel characterization of cW11 nucleosome ligation products. (**A**) Representative SDS-PAGE of 147 bp H3(aa33-135) nucleosome starting material, products of the cW11 sortase ligation, and purified H3(aa33-135) octamer prior to TEV cleavage. (**B**) Representative TBE native gel of 147 bp H3(aa33-135) nucleosome starting material, products of the cW11 sortase ligation. (**C**) Representative TBE native gel of 147 bp products of the cW11 sortase ligation, and an unmodified 147 bp nucleosome prepared by traditional nucleosome reconstitution. (**D**) Representative TBE native gel of 147 bp products of the cW11 sortase ligation.

Figure S11. Mass spectrometric characterization of peptide substrates used in cW11 nucleosome ligation. Raw spectra deconvoluted with UniDec.[19]

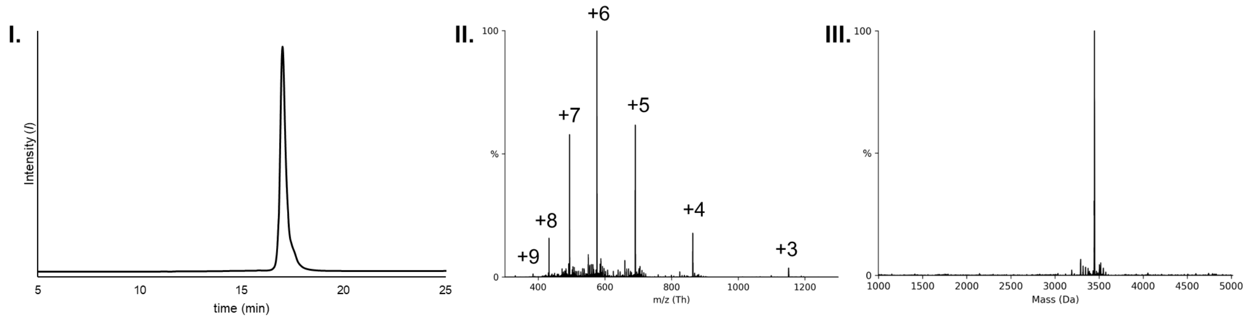

A. H3(1-34) unmodified T32-G33 amide linkage. I. Analytical RP-HPLC chromatogram (C18, 0-30% B, 23 min). **II.** Intact peptide ESI-MS. **III.** Deconvoluted peptide ESI-MS (Calculated exact mass for $C_{144}H_{261}N_{55}O_{43}$ $[M]^+$: 3448.99 Da; Observed: 3448.8 Da).

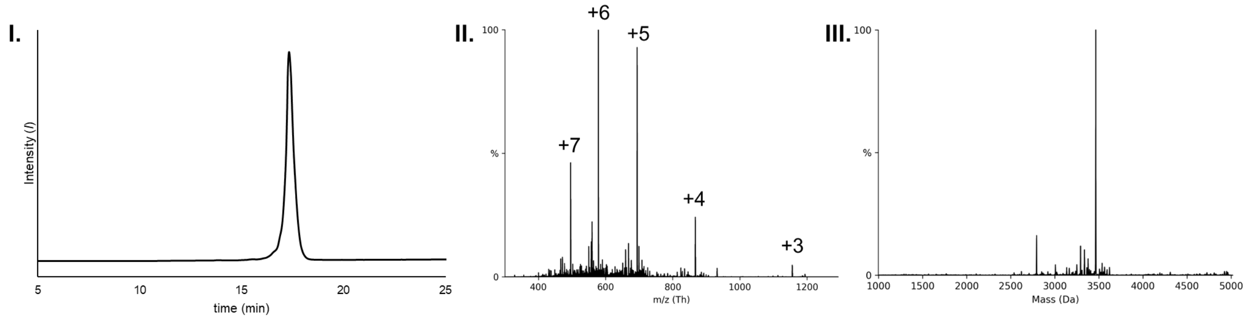

B. H3(1-34) K4me1 T32-G33 amide linkage. I. Analytical RP-HPLC chromatogram (C18, 0-30% B, 23 min). **II.** Intact peptide ESI-MS. **III.** Deconvoluted peptide ESI-MS (Calculated exact mass for $C_{145}H_{263}N_{55}O_{43}$ $[M]^+$: 3463.01 Da; Observed: 3463.8 Da).

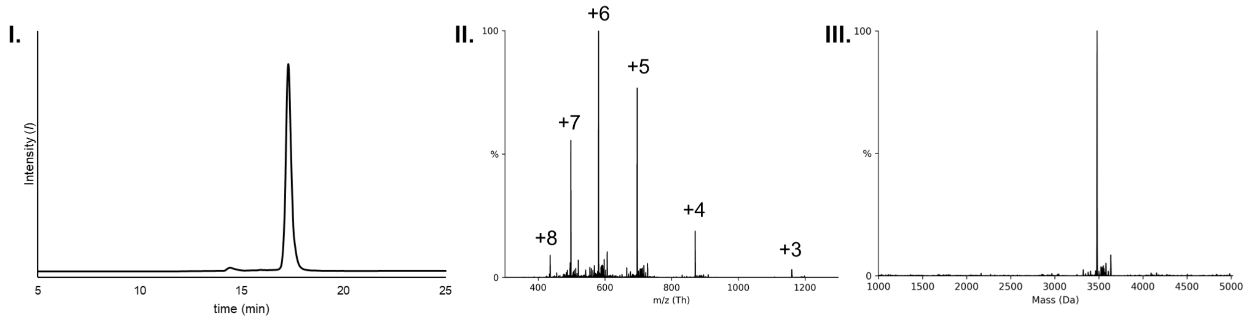

C. H3(1-34) K4me2 T32-G33 amide linkage. I. Analytical RP-HPLC chromatogram (C18, 0-30% B, 23 min). **II.** Intact peptide ESI-MS. **III.** Deconvoluted peptide ESI-MS (Calculated exact mass for $C_{146}H_{265}N_{55}O_{43}$ $[M]^+$: 3477.02 Da; Observed: 3476.9 Da).

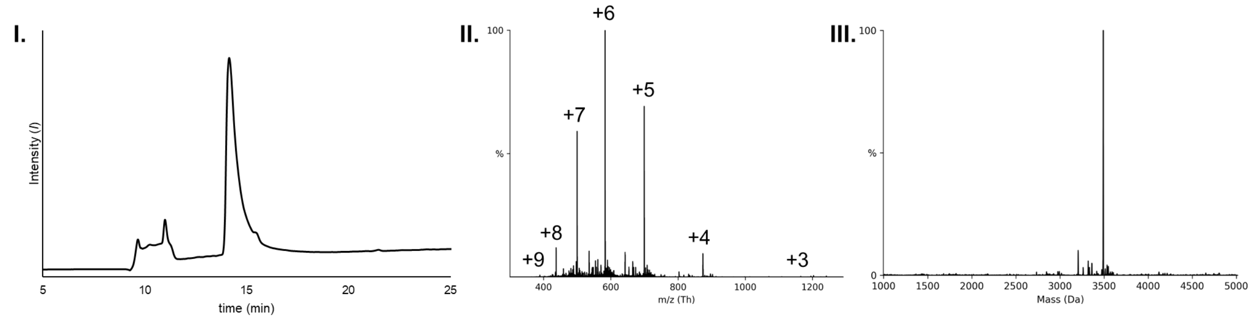

D. H3(1-34) K4me3 T32-G33 amide linkage. I. Analytical RP-HPLC chromatogram (C18, 7-30% B, 30 min). **II.** Intact peptide ESI-MS. **III.** Deconvoluted peptide ESI-MS (Calculated exact mass for $C_{148}H_{266}N_{54}O_{46}$ $[M]^+$: 3492.05 Da; Observed: 3491.1 Da).

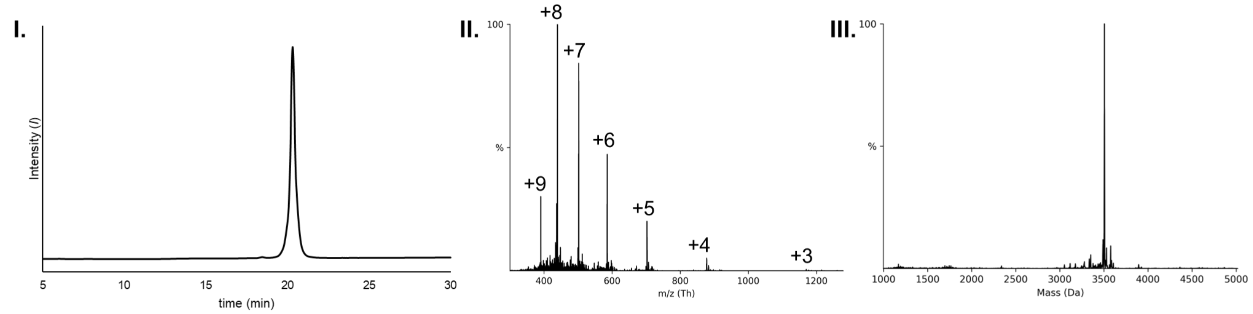

E. H3(1-34) K9-propionyl T32-G33 depsipeptide. I. Analytical RP-HPLC chromatogram (C18, 7-30% B, 30 min). **II.** Intact peptide ESI-MS. **III.** Deconvoluted peptide ESI-MS (Calculated exact mass for $C_{148}H_{266}N_{54}O_{45}$ $[M]^+$: 3520.02 Da; Observed: 3519.7 Da).

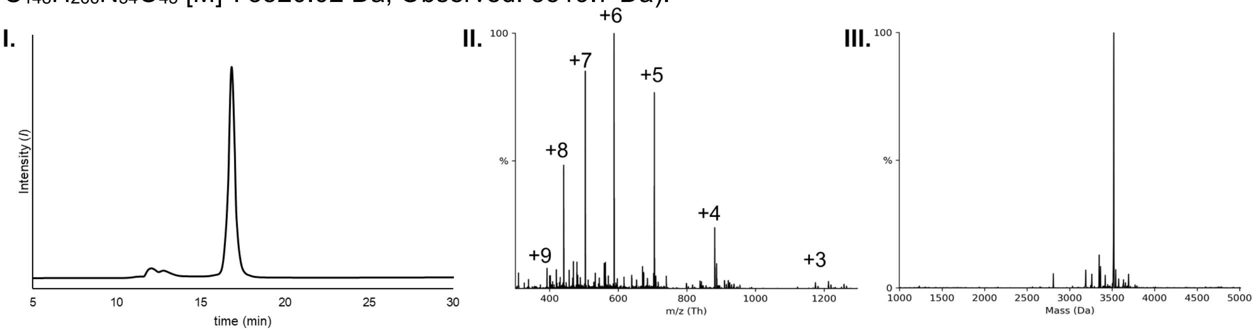

F. H3(1-34) K9-butyryl T32-G33 depsipeptide. I. Analytical RP-HPLC chromatogram (C18, 7-30% B, 30 min). **II.** Intact peptide ESI-MS. **III.** Deconvoluted peptide ESI-MS (Calculated exact mass for $C_{148}H_{266}N_{54}O_{45}$ $[M]^+$: 3520.02 Da; Observed: 3519.7 Da).

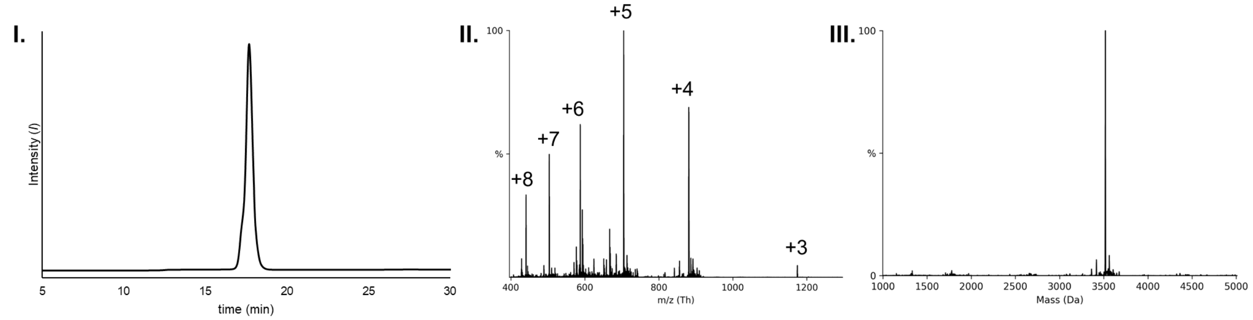

G. H3(1-34) K9-crotonyl T32-G33 depsipeptide. I. Analytical RP-HPLC chromatogram (C18, 7-30% B, 30 min). **II.** Intact peptide ESI-MS. **III.** Deconvoluted peptide ESI-MS (Calculated exact mass for $C_{148}H_{264}N_{54}O_{45}$ $[M]^+$: 3518.00 Da; Observed: 3518.7 Da).

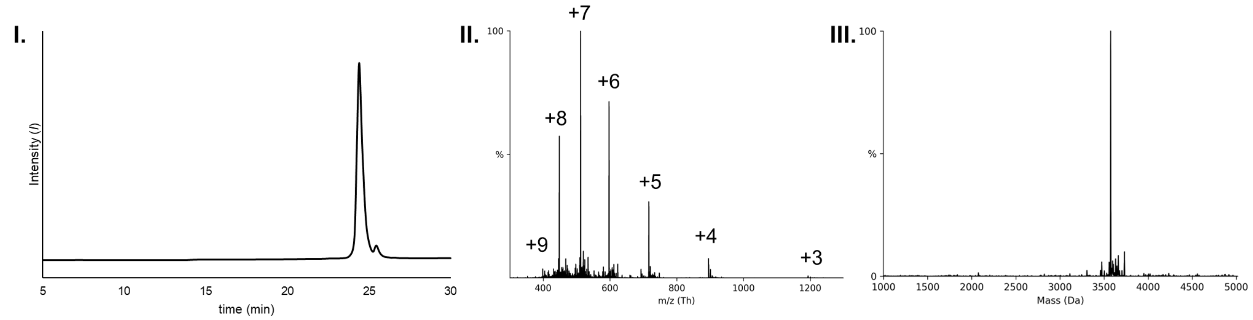

H. H3(1-34) K9-octanoyl T32-G33 depsipeptide. I. Analytical RP-HPLC chromatogram (C18, 0-30% B, 23 min). **II.** Intact peptide ESI-MS. **III.** Deconvoluted peptide ESI-MS (Calculated exact mass for $C_{152}H_{274}N_{54}O_{45}$ $[M]^{+}$: 3576.08 Da; Observed: 3575.7 Da).

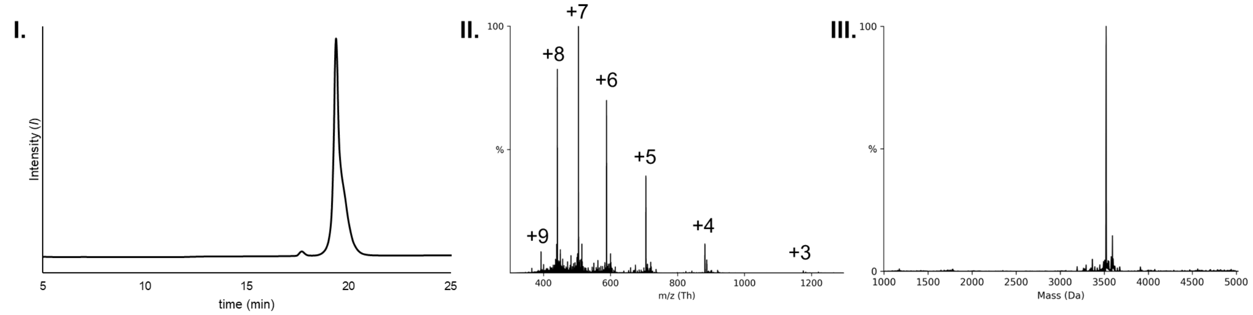

I. H3(1-34) K9-lactyl T32-G33 depsipeptide. I. Analytical RP-HPLC chromatogram (C18, 0-30% B, 23 min). **II.** Intact peptide ESI-MS. **III.** Deconvoluted peptide ESI-MS (Calculated exact mass for $C_{147}H_{264}N_{54}O_{46}$ $[M]^{+}$: 3522.00 Da; Observed: 3521.5 Da).

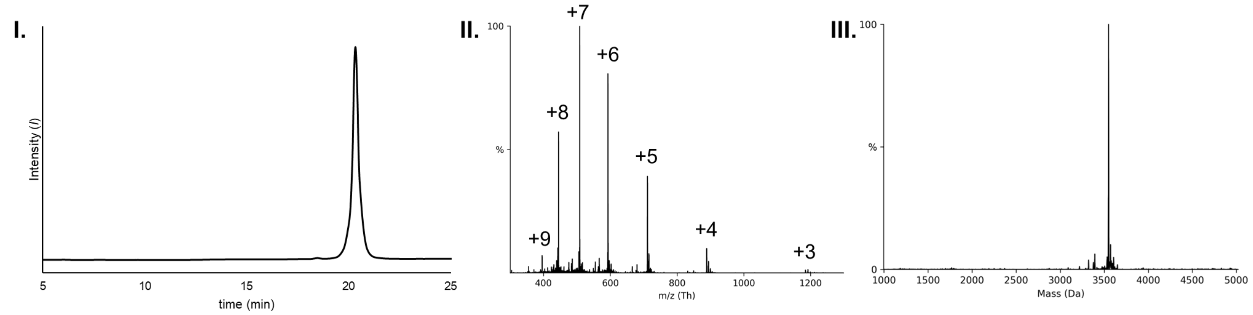

J. H3(1-34) K9-succinyl T32-G33 depsipeptide. I. Analytical RP-HPLC chromatogram (C18, 0-30% B, 23 min). **II.** Intact peptide ESI-MS. **III.** Deconvoluted peptide ESI-MS (Calculated exact mass for $C_{148}H_{264}N_{54}O_{47}$ $[M]^{+}$: 3549.99 Da; Observed: 3549.4 Da).

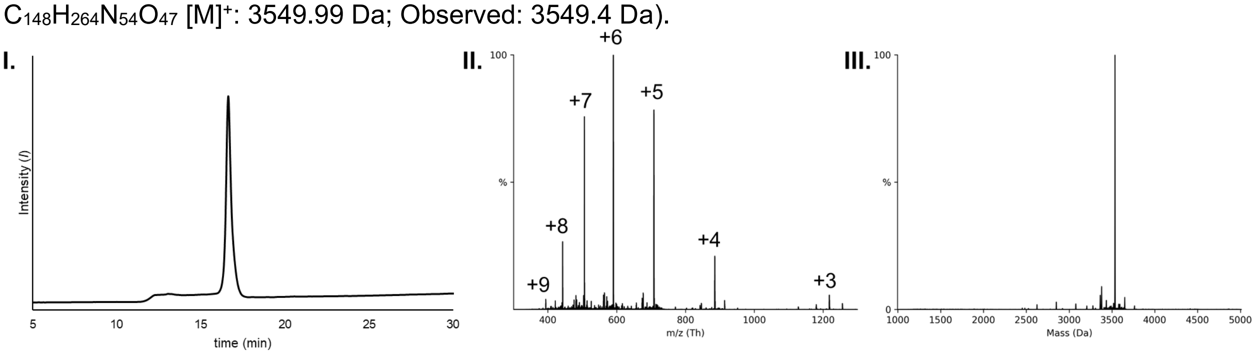

K. H3(1-34) K9-anti-hydroxyisbutyryl T32-G33 depsipeptide. I. Analytical RP-HPLC chromatogram (C18, 7-30% B, 30 min). **II.** Intact peptide ESI-MS. **III.** Deconvoluted peptide ESI-MS (Calculated exact mass for $C_{148}H_{266}N_{54}O_{46}$ $[M]^{+}$: 3536.01 Da; Observed: 3535.8 Da).

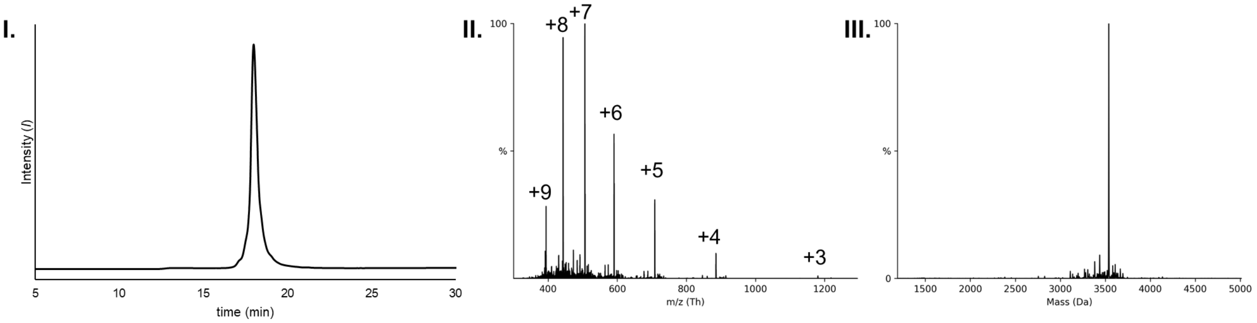

L. H3(1-34) K9-β-hydroxybutyryl T32-G33 depsipeptide. I. Analytical RP-HPLC chromatogram (C18, 7-30% B, 30 min). **II.** Intact peptide ESI-MS. **III.** Deconvoluted peptide ESI-MS (Calculated exact mass for $C_{148}H_{266}N_{54}O_{46}$ $[M]^{+}$: 3536.01 Da; Observed: 3536.3 Da).

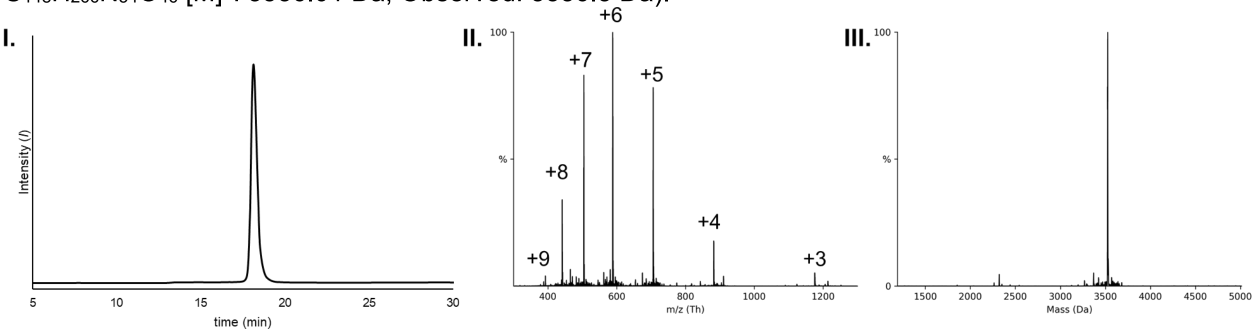

M. H3(1-34) K9-N-methylthiourea T32-G33 depsipeptide. I. Analytical RP-HPLC chromatogram (C18, 7-30% B, 30 min). **II.** Intact peptide ESI-MS. **III.** Deconvoluted peptide ESI-MS (Calculated exact mass for $C_{146}H_{263}N_{55}O_{44}S$ $[M]^{+}$: 3522.98 Da; Observed: 3523.7 Da).

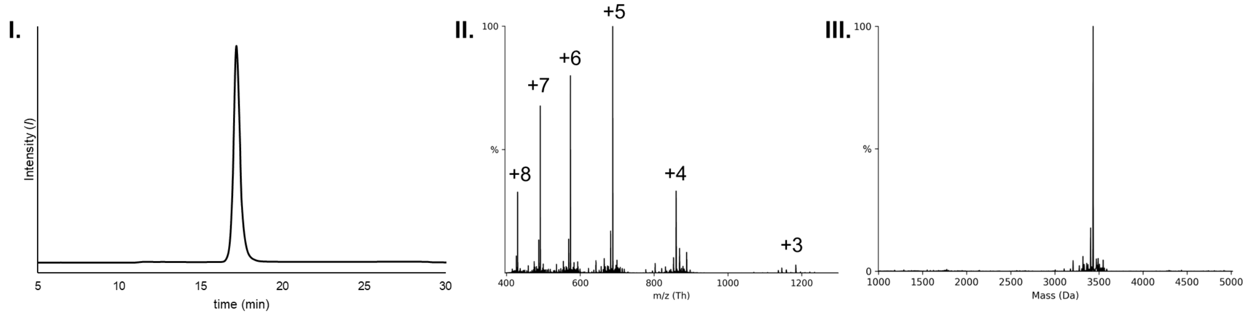

N. H3(1-34) K9norleucine T32-G33 depsipeptide. I. Analytical RP-HPLC chromatogram (C18, 7-30% B, 30 min). **II.** Intact peptide ESI-MS. **III.** Deconvoluted peptide ESI-MS (Calculated exact mass for $C_{144}H_{259}N_{53}O_{44}$ $[M]^{+}$: 3433.98 Da; Observed: 3433.9 Da).

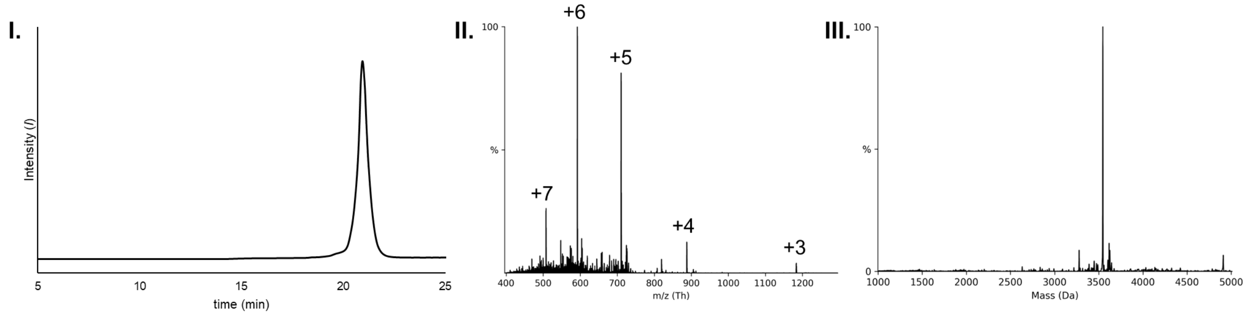

O. H3(1-34) K9-myristoyl T32-G33 depsipeptide. I. Analytical RP-HPLC chromatogram (C18, 7-30% B, 30 min). **II.** Intact peptide ESI-MS. **III.** Deconvoluted peptide ESI-MS (Calculated exact mass for $C_{158}H_{286}N_{54}O_{45}$ $[M]^{+}$: 3660.18 Da; Observed: 3659.8 Da).

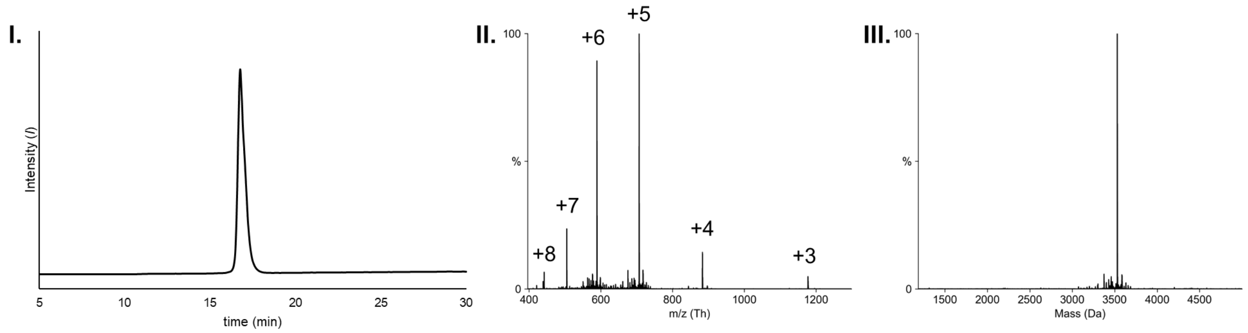

P. H3(1-34) S28-phosphoryl T32-G33 depsipeptide. I. Analytical RP-HPLC chromatogram (C18, 0-65% B, 30 min). **II.** Intact peptide ESI-MS. **III.** Deconvoluted peptide ESI-MS (Calculated exact mass for $C_{144}H_{260}N_{54}O_{47}P$ $[M]^+$: 3528.94 Da; Observed: 3529.0 Da).

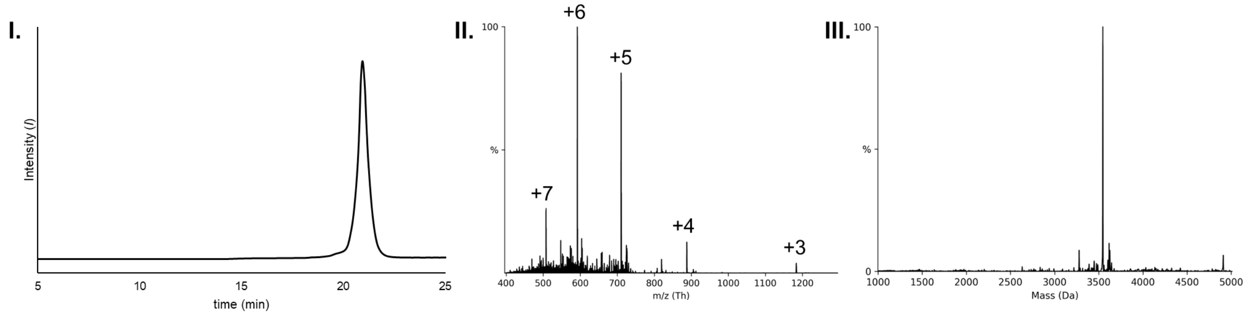

Q. H3(1-34) S31-phosphoryl T32-G33 depsipeptide. I. Analytical RP-HPLC chromatogram (C18, 7-30% B, 30 min). **II.** Intact peptide ESI-MS. **III.** Deconvoluted peptide ESI-MS (Calculated exact mass for $C_{144}H_{260}N_{54}O_{48}P$ $[M]^+$: 3544.93 Da; Observed: 3546.1 Da).

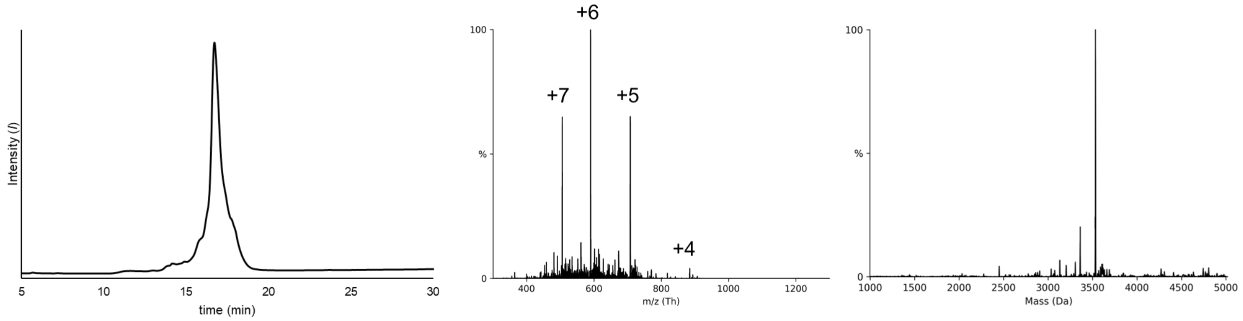

R. H3(1-34) K4-trimethyl K9-acetyl T32-G33 depsipeptide. I. Analytical RP-HPLC chromatogram (C18, 7-30% B, 30 min). **II.** Intact peptide ESI-MS. **III.** Deconvoluted peptide ESI-MS (Calculated exact mass for $C_{144}H_{260}N_{54}O_{48}P$ $[M]^+$: 3544.93 Da; Observed: 3546.1 Da).

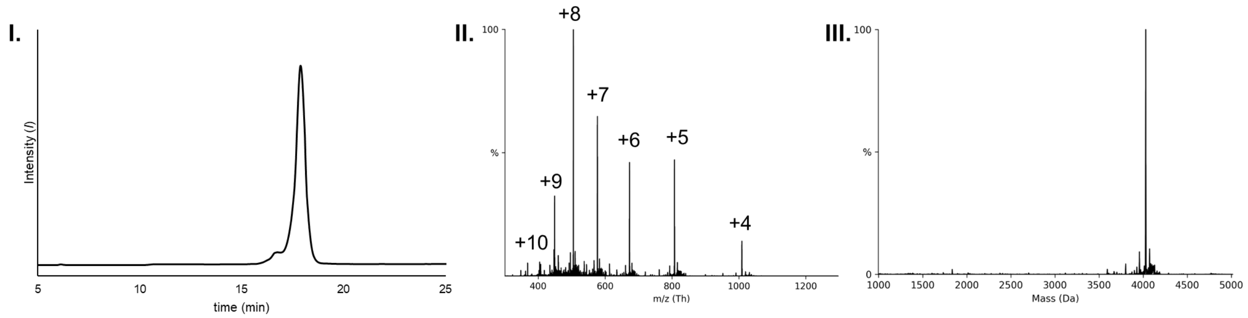

S. H3(1-34) K9-acetyl K14-acetyl K18-acetyl K27-acetyl T32-G33 depsipeptide (aa35-37) KRK. I. Analytical RP-HPLC chromatogram (C18, 7-30% B, 30 min). **II.** Intact peptide ESI-MS. **III.** Deconvoluted peptide ESI-MS (Calculated exact mass for $C_{170}H_{305}N_{62}O_{51}$ $[M]^+$: 4030.31 Da; Observed: 4029.1 Da).

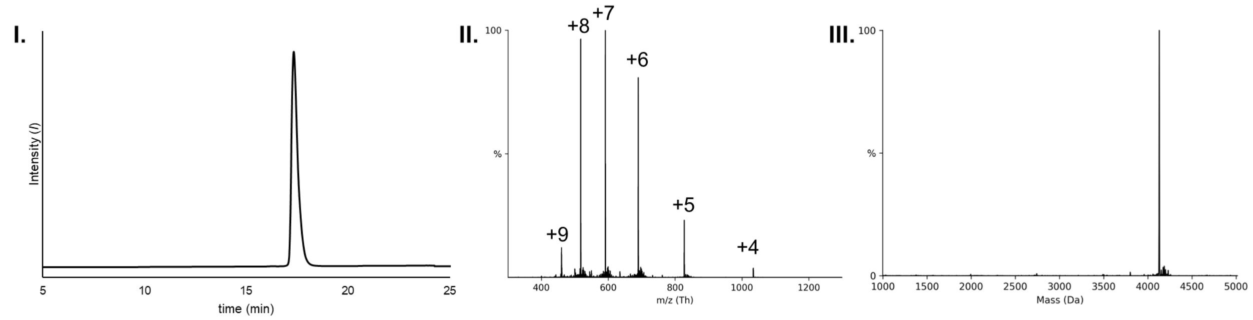

T. H3(1-34) K9-acetyl K14-acetyl K18-acetyl K23-acetyl T32-G33 depsipeptide (aa35-38) KKKK. I. Analytical RP-HPLC chromatogram (C18, 7-30% B, 30 min). **II.** Intact peptide ESI-MS. **III.** Deconvoluted peptide ESI-MS (Calculated exact mass $C_{176}H_{316}N_{62}O_{52}$ $[M]^{+}$: 4130.40 Da; Observed: 4130.3 Da).

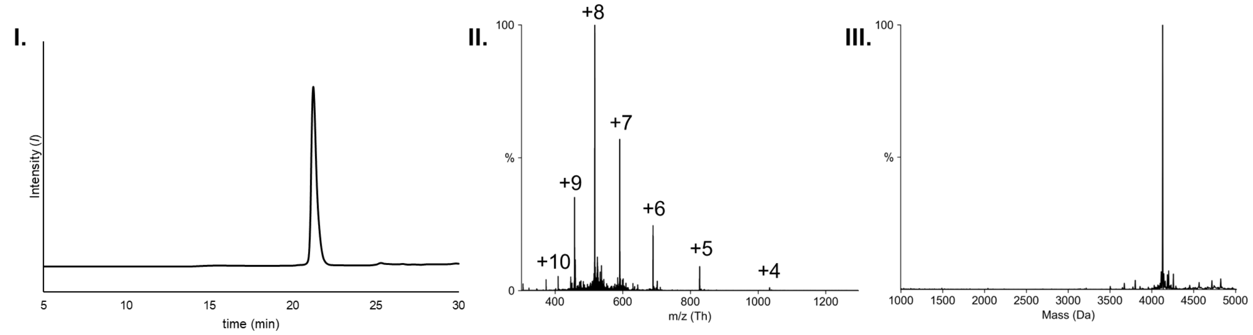

U. H3(1-34) K9-acetyl K14-acetyl K23-acetyl K27-acetyl T32-G33 depsipeptide (aa35-38) KKKK. I. Analytical RP-HPLC chromatogram (C18, 7-30% B, 30 min). **II.** Intact peptide ESI-MS. **III.** Deconvoluted peptide ESI-MS (Calculated exact mass $C_{176}H_{316}N_{62}O_{52}$ $[M]^{+}$: 4130.40 Da; Observed: 4130.0 Da).

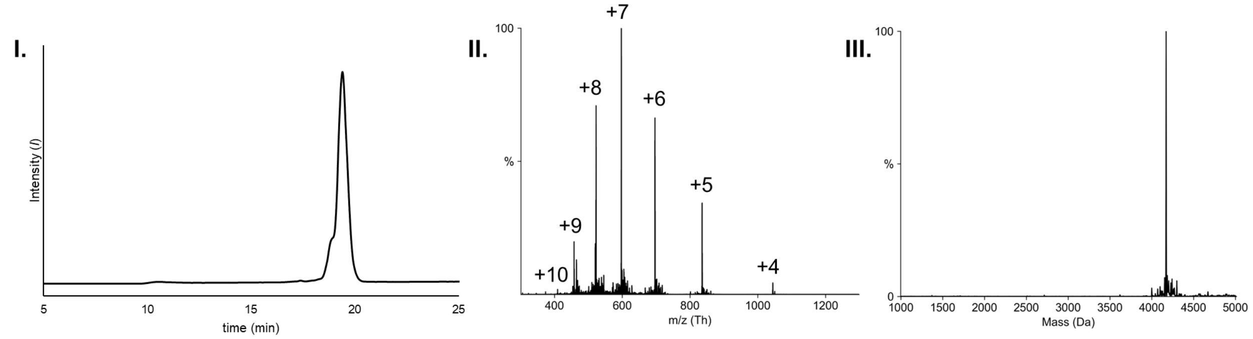

V. H3(1-34) K9-acetyl K14-acetyl K18-acetyl K23-acetyl K27-acetyl T32-G33 depsipeptide (aa35-38) KKKK. I. Analytical RP-HPLC chromatogram (C18, 7-30% B, 30 min). **II.** Intact peptide ESI-MS. **III.** Deconvoluted peptide ESI-MS (Calculated exact mass $C_{178}H_{318}N_{62}O_{53}$ $[M]^{+}$: 4172.41 Da; Observed: 4172.0 Da)

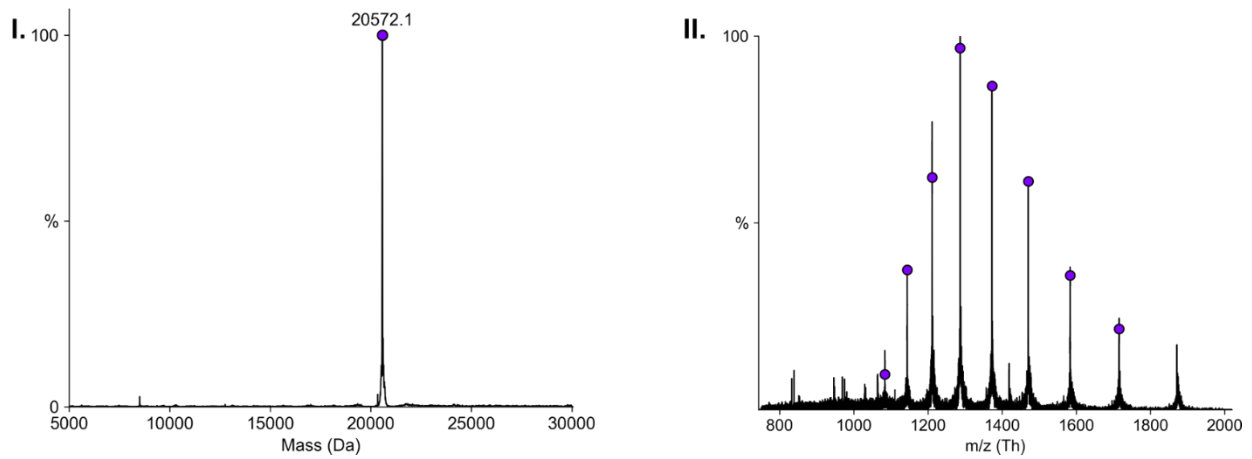

W. H3(1-34) K18-Ubiquitin(G76A) K23-Ubiquitin(G76A) T32-G33 amide. I. Deconvoluted peptide ESI-MS (Calculated exact mass $C_{902}H_{1519}N_{265}O_{277}S_2$ $[M]^+$: 20572.45 Da; Observed: 20572.1 Da). **II.** Intact peptide ESI-MS.

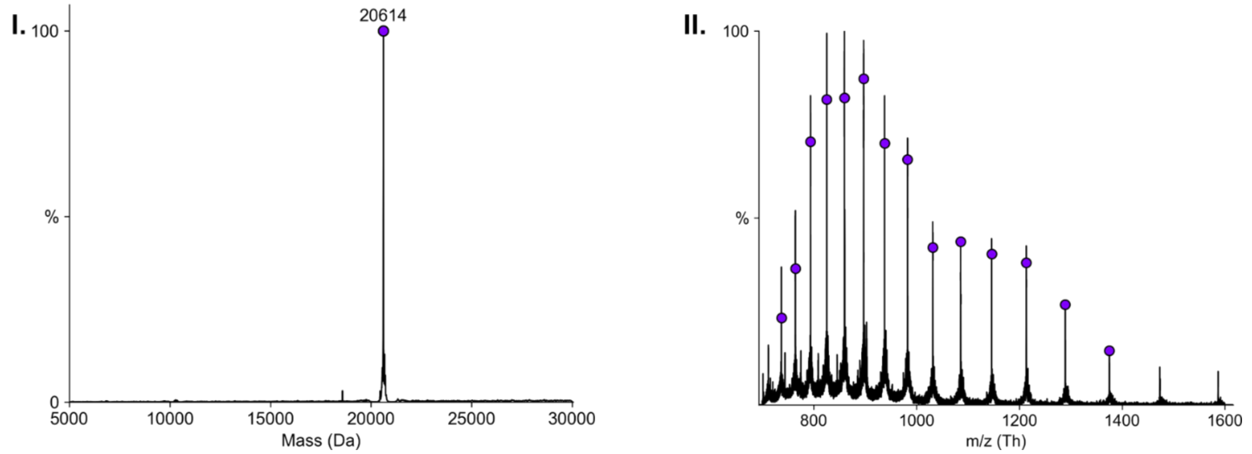

X. H3(1-34) K9-trimethyl K18-Ubiquitin(G76A) K23-Ubiquitin(G76A) T32-G33 amide. I. Deconvoluted peptide ESI-MS (Calculated exact mass for $C_{905}H_{1525}N_{265}O_{277}S_2$ $[M]^+$: 20614.53 Da; Observed: 20614.0 Da). **II.** Intact peptide ESI-MS.

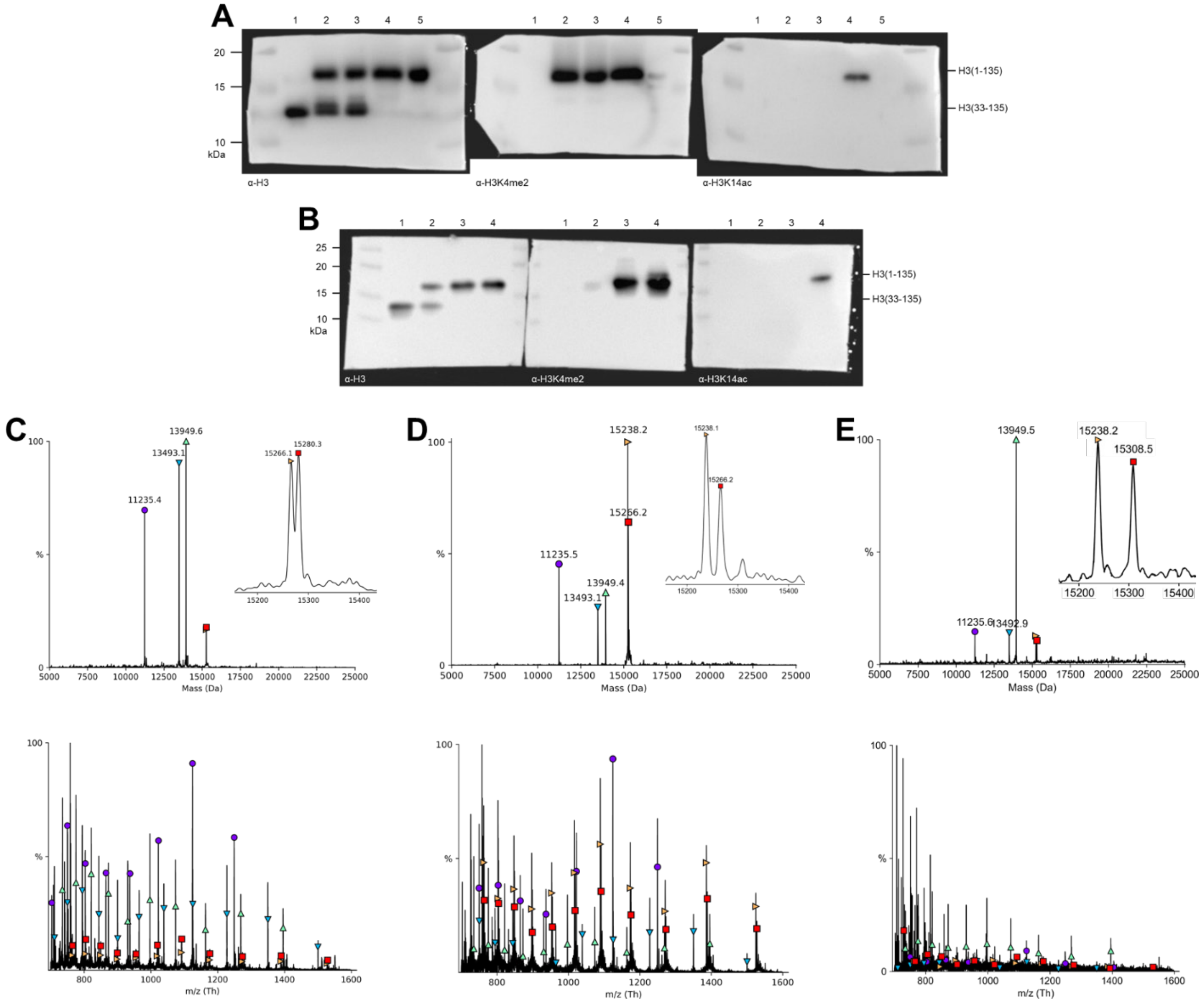

Figure S12. Characterization of asymmetric H3K4me2 H3K14ac ligation intermediate and final products. (**A**) Anti-H3 (left), anti-H3K4me2 (middle), and anti-H3K14ac (right) western blot visualization of asymmetric K4me2 nucleosome synthesis starting material, intermediates, and final products: H3 (aa33-135) starting material; (2) asymmetric H3K4me2 & H3 (aa33-135) intermediate; (3) asymmetric H3K4me2 & H3 (aa33-135) intermediate; (4) asymmetric H3K4me2 & H3K14ac product; (5) asymmetric H3K4me2 & unmodified H3 product. (**B**) Anti-H3 (left), anti-H3K4me2 (middle), and anti-H3K14ac (right) western blot visualization of asymmetric K4me2 nucleosome synthesis starting material, intermediates, and final products: H3 (aa33-135) starting material; (2) asymmetric H3K4me2 & H3 (aa33-135) intermediate; (3) asymmetric H3K4me2 & unmodified H3 product (4) asymmetric H3K4me2 & H3K14ac product. (**C**) Deconvoluted mass spectrum (top) and raw mass spectrum (bottom) of 185 bp asymmetric H3K4me2 & H3K14ac product: H4 (purple circle) calculated mass 11236.15 Da, found: 11235.4 Da; H2B (teal downward pointed triangle) calculated mass 13493.68 Da, found: 13493.1 Da; H2A (green upward pointed triangle) calculated mass 13950.2 Da, found: 13949.6 Da; H3K4me2 (orange rightward pointed triangle) calculated mass 15267.62 Da, found: 15266.1 Da; H3K14ac (red square) calculated mass 15281.61 Da, found: 15280.3 Da. (**D**) Deconvoluted mass spectrum (top) and raw mass spectrum (bottom) of 185 bp asymmetric unmodified H3 & H3K4me2 product: H4 (purple circle) calculated mass 11236.15 Da, found: 11235.5 Da; H2B (teal downward pointed triangle) calculated mass 13493.68 Da, found: 13493.1 Da; H2A (green upward pointed triangle) calculated mass 13950.2 Da, found: 13949.4 Da; H3 (orange rightward pointed triangle) calculated mass 15238.61 Da, found: 15238.1 Da; H3K4me2 (red square) calculated mass 15266.66 Da, found: 15266.2 Da. (**E**) Deconvoluted mass spectrum (top) and raw mass spectrum (bottom) of 185 bp asymmetric unmodified H3 & H3K4me2K14ac product: H4

(purple circle) calculated mass 11236.15 Da, found: 11235.6 Da; H2B (teal downward pointed triangle) calculated mass 13493.68 Da, found: 13492.9 Da; H2A (green upward pointed triangle) calculated mass 13950.2 Da, found: 13949.5 Da; H3 (orange rightward pointed triangle) calculated mass 15238.61 Da, found: 15238.2 Da; H3K4me2K14ac (red square) calculated mass 15309.71 Da, found: 15308.5 Da. Raw spectra deconvoluted with UniDec.[19]

A

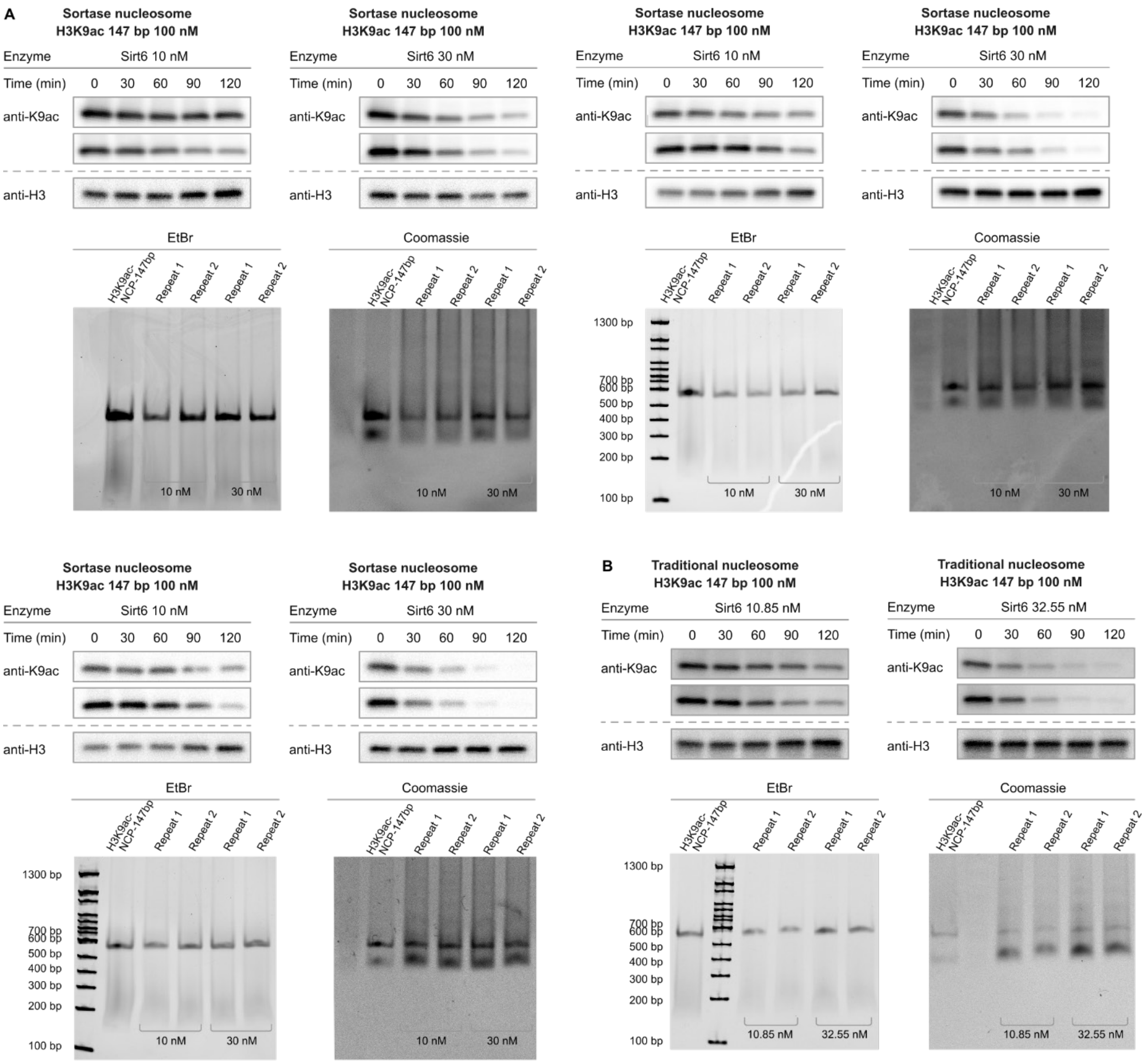

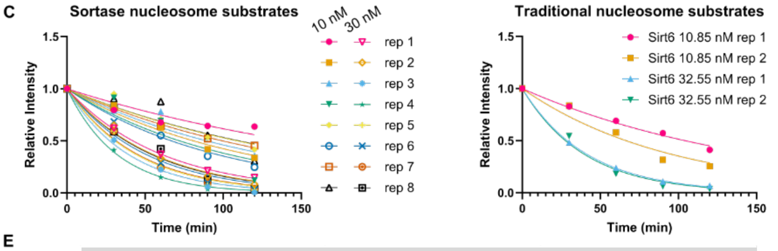

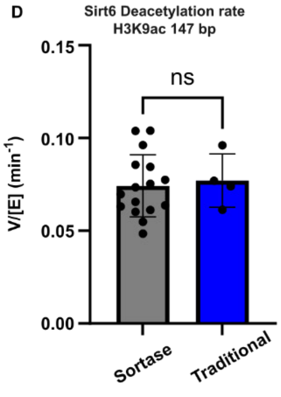

E

Sirt6 Nucleosome	Sortase nucleosome	Traditional nucleosome
H3K9ac	0.075±0.0044	0.076±0.0030

Figure S13. Comparison of Sirt6 activity toward nucleosomes prepared by conventional reconstitution and cW11 nucleosome ligation. (**A**) Representative western blots (top; n=12 of n=16) and native TBE gels (bottom) of Sirt6 deacetylation assays with 147 bp H3K9ac nucleosome prepared by

sortase ligation. (**B**) Western blots (top; n=4) and native TBE gels (bottom) of Sirt6 deacetylation assays with 147 bp H3K9ac nucleosome prepared by traditional nucleosome reconstitution. (**C**) Deacylation fitting for sortase nucleosomes (left; GraphPad Prism 10.2.3; one-phase decay) and nucleosomes prepared by literature protocols (left; GraphPad Prism 10.2.3; one-phase decay) from **A** and **B** respectively. (**D**) Sirt6 deacetylation rates (V/[E]) for H3K9ac nucleosomes prepared by the cW11 sortase ligation (Sortase) and nucleosomes prepared by literature protocols (Traditional); error bars indicate standard deviation. (**E**) Average V/[E] ± SEM values for Sirt6 deacetylation of H3K9ac nucleosomes prepared by either cW11 sortase ligation (n=16) or literature protocols (n=4).

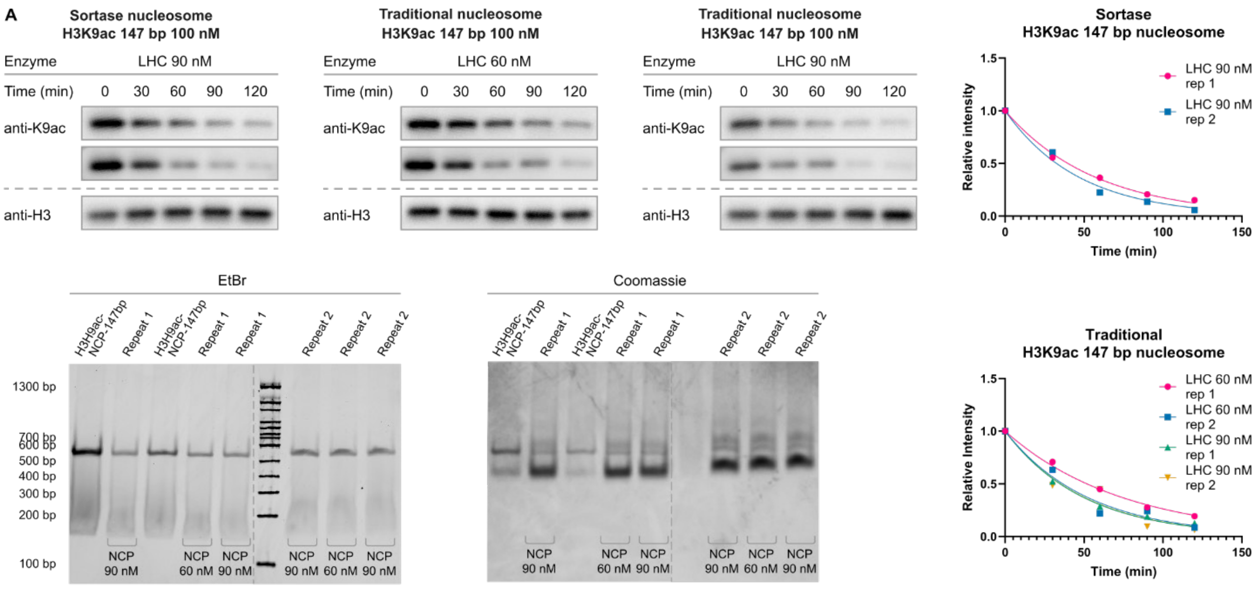

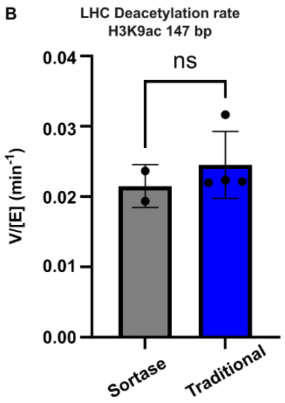

C

LHC Nucleosome	Sortase nucleosome	Traditional nucleosome
H3K9ac	0.021±0.0011	0.026±0.0021

Figure S14. Comparison of LSD1/HDAC1/CoREST activity toward nucleosomes prepared by conventional reconstitution and cW11 nucleosome ligation. (**A**) Western blots (top), deacylation fitting (right; GraphPad Prism 10.2.3; one-phase decay), and native TBE gels (bottom) of LSD1/HDAC1/CoREST (LHC) deacetylation assays with 147 bp H3K9ac nucleosome prepared by the sortase ligation (top left; n=2) or traditional nucleosome reconstitution (top right; n=4). (**B**) LHC complex deacetylation rates (V/[E]) for H3K9ac nucleosomes prepared by the cW11 sortase ligation (Sortase) and nucleosomes prepared by literature protocols (Traditional); error bars indicate standard deviation. (**C**) Average V/[E] ± SEM values for LHC deacetylation of H3K9ac nucleosomes prepared by either cW11 sortase ligation or literature protocols.

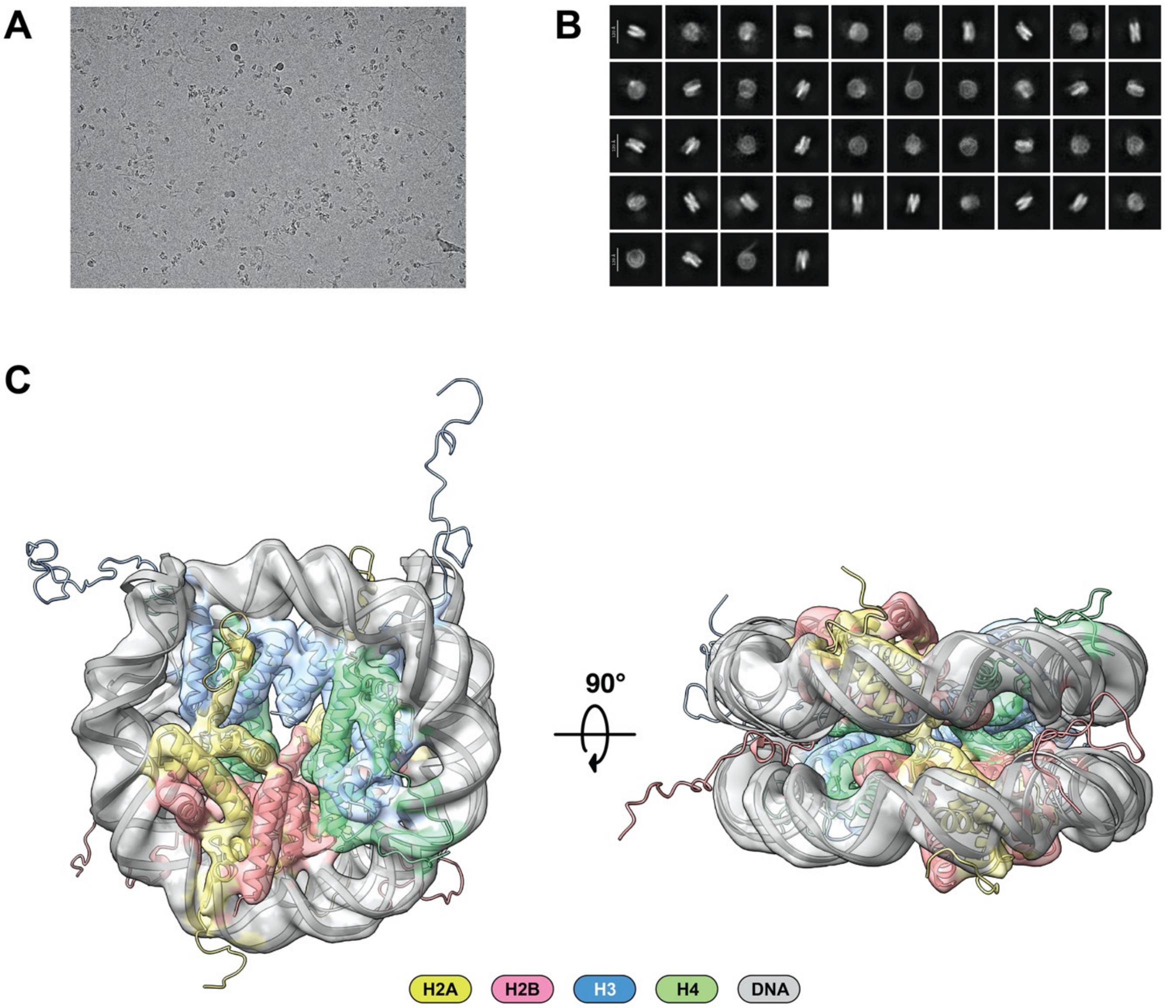

Figure S15. Cryo electron microscopy characterization of nucleosomes prepared by cW11 nucleosome ligation. (**A**) Raw cryo-EM images of cW11 nucleosome. (**B**) Representative 2D Class averages from 14300 particles. (**C**) 4.7-Å cryo-EM reconstruction map of cW11 nucleosome displayed in two separate views related by 90°. A structural model of canonical *X. laevis* nucleosome (PDBID: 1KX5) is docked in to the EM density. The EM reconstruction and the model were colored the following way: DNA in gray, histone H2A in pale yellow, histone H2B in red salmon, histone H3 in marine blue, and histone H4 in lime green.

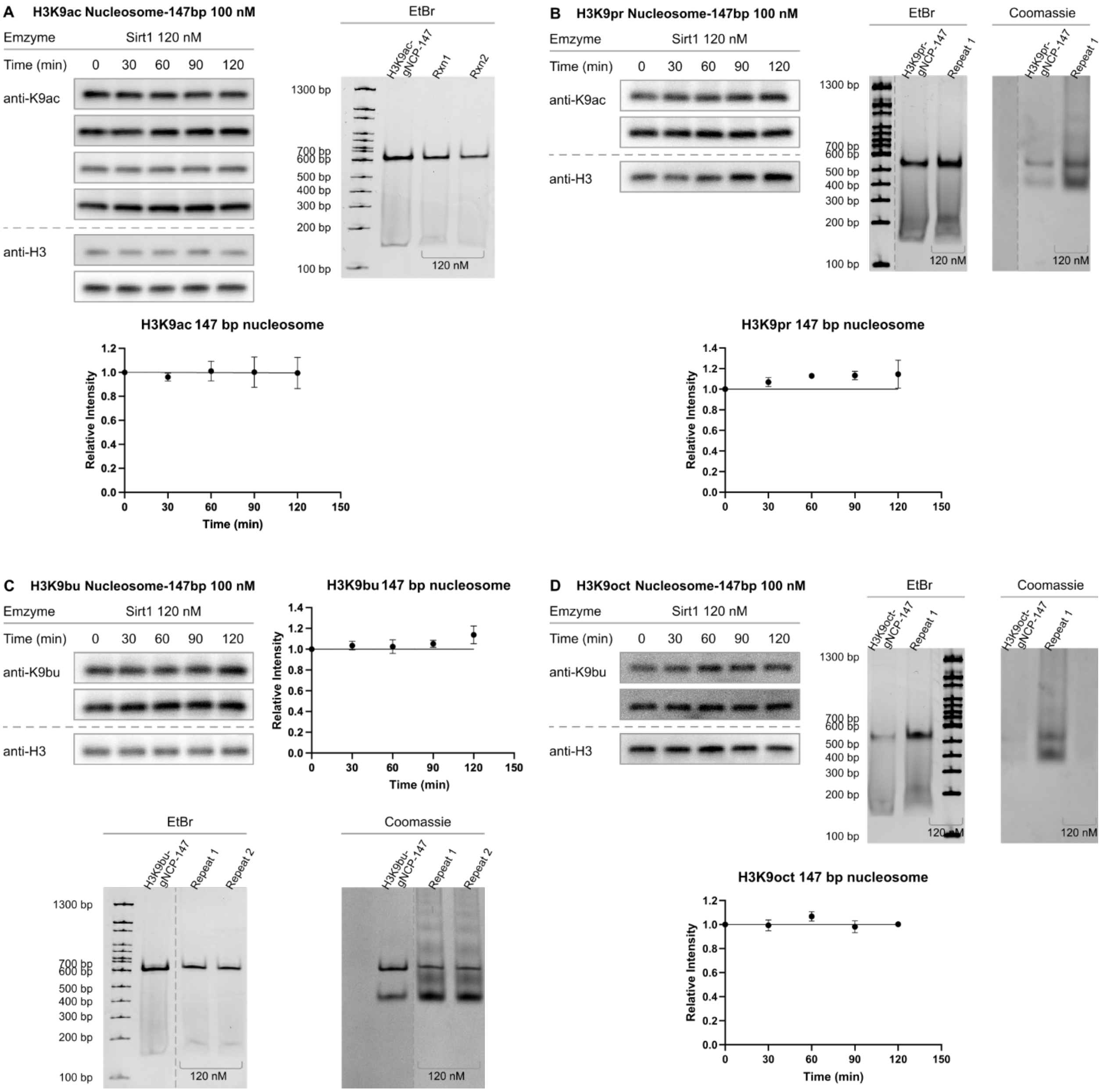

Figure S16. Western blot measurement of Sirt1 activity by length of H3K9 acylation carbon chain. (**A**) Western blots (top left; anti-H3K9ac; n=4), deacylation fitting (bottom; GraphPad Prism 10.2.3; one-phase decay), and native TBE gels (top right) of Sirt1 deacetylation assays with 147bp H3K9acetyl (H3K9ac) nucleosomes. (**B**) Western blots (top left; anti-H3K9ac antibody; n=2), deacylation fitting (bottom; GraphPad Prism 10.2.3; one-phase decay), and native TBE gels (top right) of Sirt1 deacetylation assays with 147 bp H3K9propionyl (H3K9pr) nucleosomes. (**C**) Western blots (top left; anti-H3K9bu; n=2), deacylation fitting (top right; GraphPad Prism 10.2.3; one-phase decay), and native TBE gels (bottom) of Sirt1 deacetylation assays with 147 bp H3K9butyryl (H3K9bu) nucleosomes. (**D**) Western blots (top left; anti-H3K9but; n=2), deacylation fitting (bottom; GraphPad Prism 10.2.3; one-phase decay), and native TBE gels (top right) of Sirt1 deacetylation assays with 147 bp H3K9octanoyl (H3K9oct) nucleosomes.

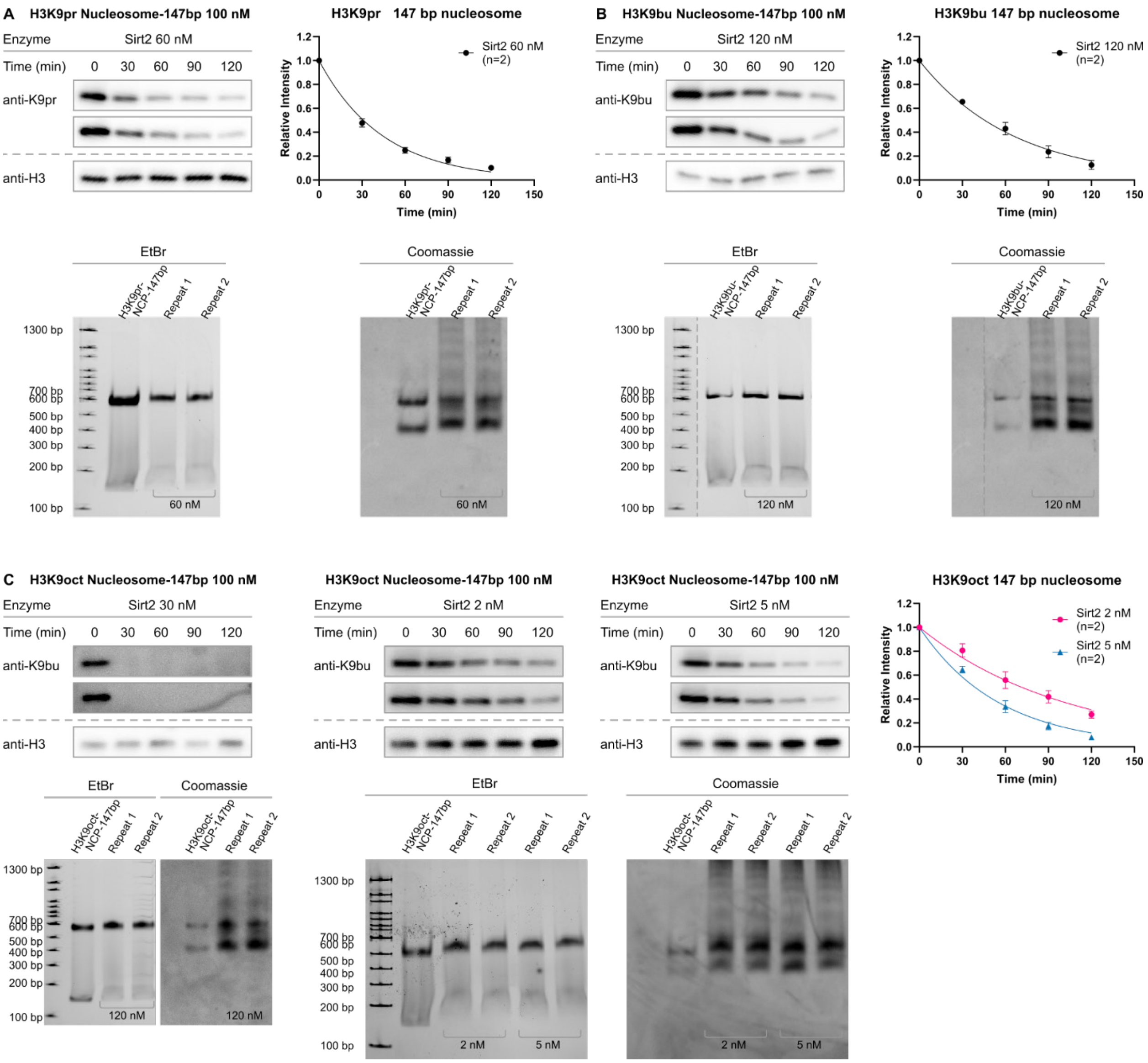

Figure S17. Western blot measurement of Sirt2 activity by length of H3K9 acylation carbon chain. (**A**) Western blots (top; anti-H3K9ac antibody; n=2), deacylation fitting (right; GraphPad Prism 10.2.3; one-phase decay), and native TBE gels (bottom) of Sirt2 deacetylation assays with H3K9propionyl (H3K9pr) nucleosomes. (**B**) Western blots (top; anti-H3K9bu; n=2), deacylation fitting (right; GraphPad Prism 10.2.3; one-phase decay), and native TBE gels (bottom) of Sirt2 deacetylation assays with H3K9butyryl (H3K9bu) nucleosomes. (**C**) Western blots (top; anti-H3K9bu; n=4), deacylation fitting (right; GraphPad Prism 10.2.3; one-phase decay), and native TBE gels (bottom) of Sirt2 deacetylation assays with H3K9octanoyl (H3K9oct) nucleosomes. Deacylation with 30 nM enzyme (leftmost) was too fast to fit, and is not included in subsequent V/[E] calculation.

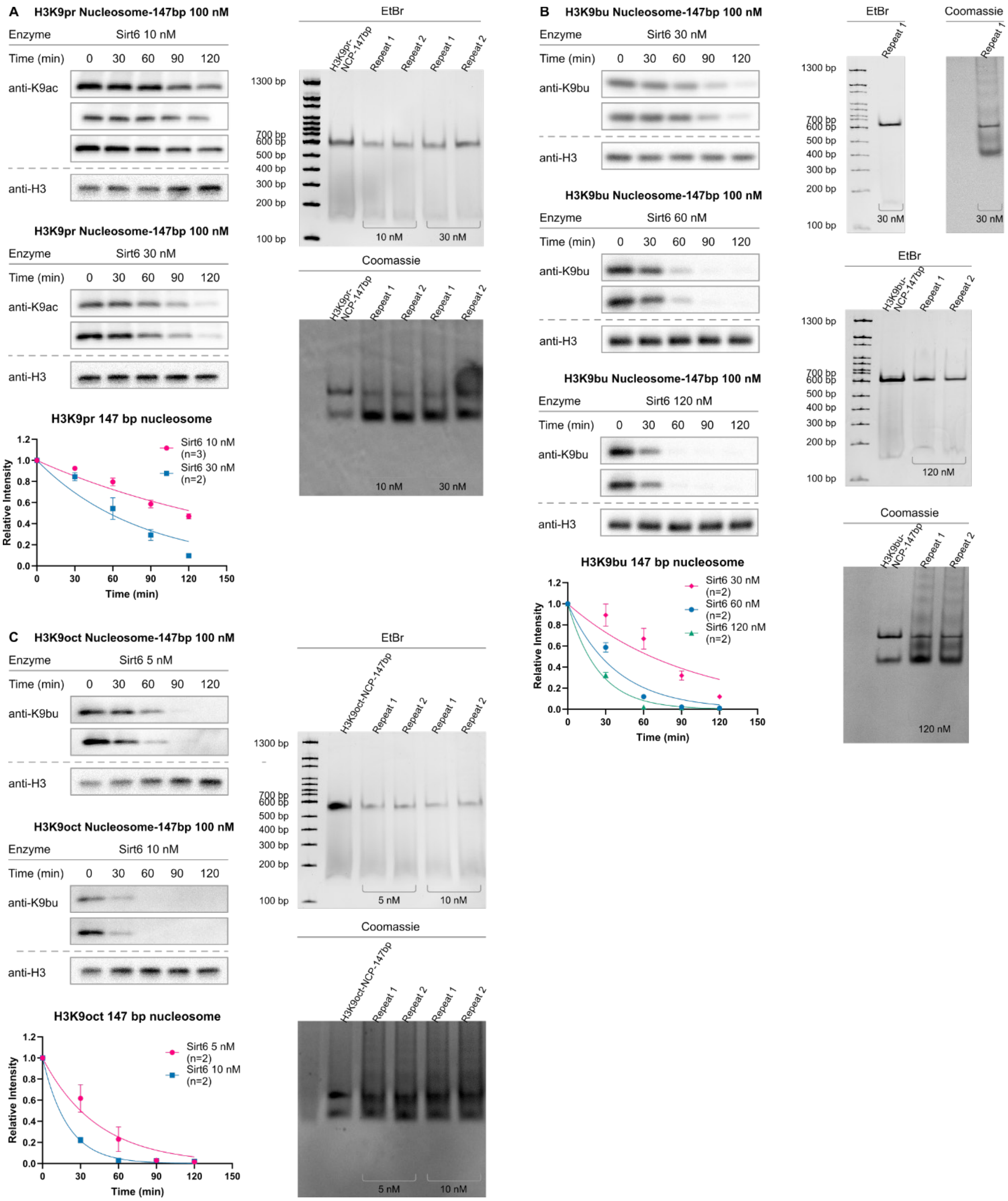

Figure S18. Western blot measurement of Sirt6 activity by length of H3K9 acylation carbon chain. (**A**) Western blots (top left; anti-H3K9ac antibody; n=5), deacylation fitting (bottom left; GraphPad Prism 10.2.3; one-phase decay), and native TBE gels (right) of Sirt6 deacetylation assays with 147 bp H3K9propionyl (H3K9pr) nucleosomes. (**B**) Western blots (top left, anti-H3K9bu; n=6), deacylation fitting (bottom left; GraphPad Prism 10.2.3; one-phase decay), and native TBE gels (right) of Sirt6 deacetylation assays with 147 bp H3K9butyryl (H3K9bu) nucleosomes. (**C**) Western blots (top left; anti-H3K9bu; n=4),

deacylation fitting (bottom left; GraphPad Prism 10.2.3; one-phase decay), and native TBE gels (right) of Sirt6 deacetylation assays with 147 bp H3K9octanoyl (H3K9oct) nucleosomes.

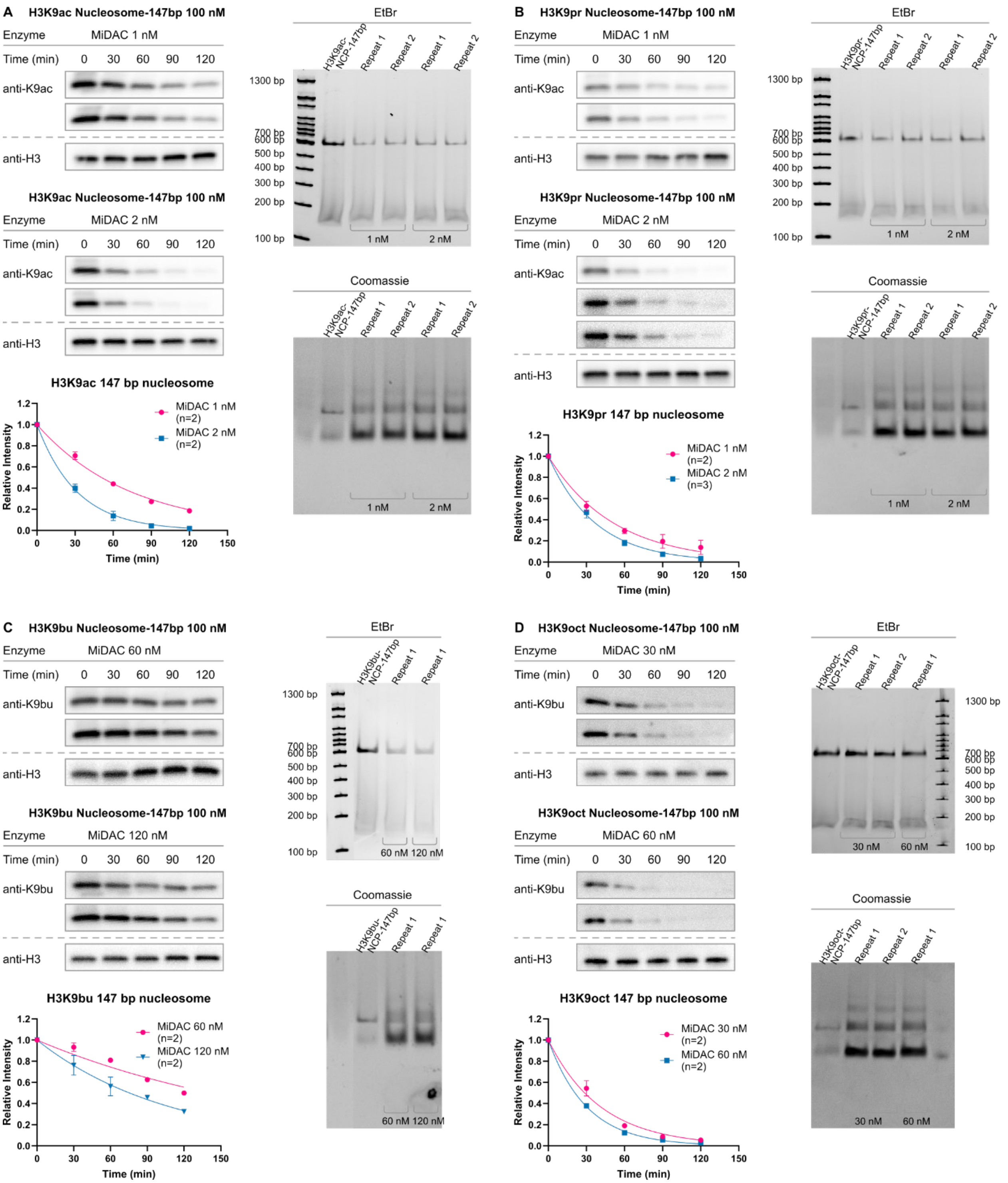

Figure S19. Western blot measurement of MiDAC activity by length of H3K9 acylation carbon chain. (**A**) Western blots (top left; anti-H3K9ac; n=4), deacylation fitting (bottom left; GraphPad Prism 10.2.3; one-phase decay), and native TBE gels (right) of MiDAC deacetylation assays with 147 bp H3K9acetyl (H3K9ac) nucleosomes. (**B**) Western (top left; anti-H3K9ac antibody; n=5), deacylation fitting (bottom left; GraphPad Prism 10.2.3; one-phase decay), and native TBE gels (right) of MiDAC deacetylation assays with 147 bp H3K9propionyl (H3K9pr) nucleosomes. (**C**) Western blots (top left; anti-H3K9bu; n=4), deacylation fitting (bottom left; GraphPad Prism 10.2.3; one-phase decay), and native TBE

gels (right) of MiDAC deacetylation assays with 147 bp H3K9butyryl (H3K9bu) nucleosomes. (**D**) Western blots ((top left; anti-H3K9bu antibody; n=4), deacylation fitting (bottom left; GraphPad Prism 10.2.3; one-phase decay), and native TBE gels (right) of MiDAC deacetylation assays with 147 bp H3K9octanoyl (H3K9oct) nucleosomes.

Acylated Nucleosome	Sirt1	Sirt2	Sirt6	MiDAC
H3K9ac	<0.002	0.026±0.0015*	0.075±0.0044	1.4±0.046
H3K9pr	<0.002	0.036±0.0022	0.041±0.0051	1.9±0.13
H3K9bu	<0.002	0.013±0.00050	0.044±0.0049	0.0077±0.00053
H3K9oct	<0.002	0.49±0.026	0.46±0.063	0.081±0.0051

Table S6. Calculated V/[E] values for HDAC activity by length of H3K9 acylation carbon chain. Average V/[E] ± SEM values for Sirt1, Sirt2, Sirt6 and MiDAC deacylation of deacylation of nucleosomes with linear acylations of increasing length. Values marked with asterisks are re-printed from prior reports.[20]

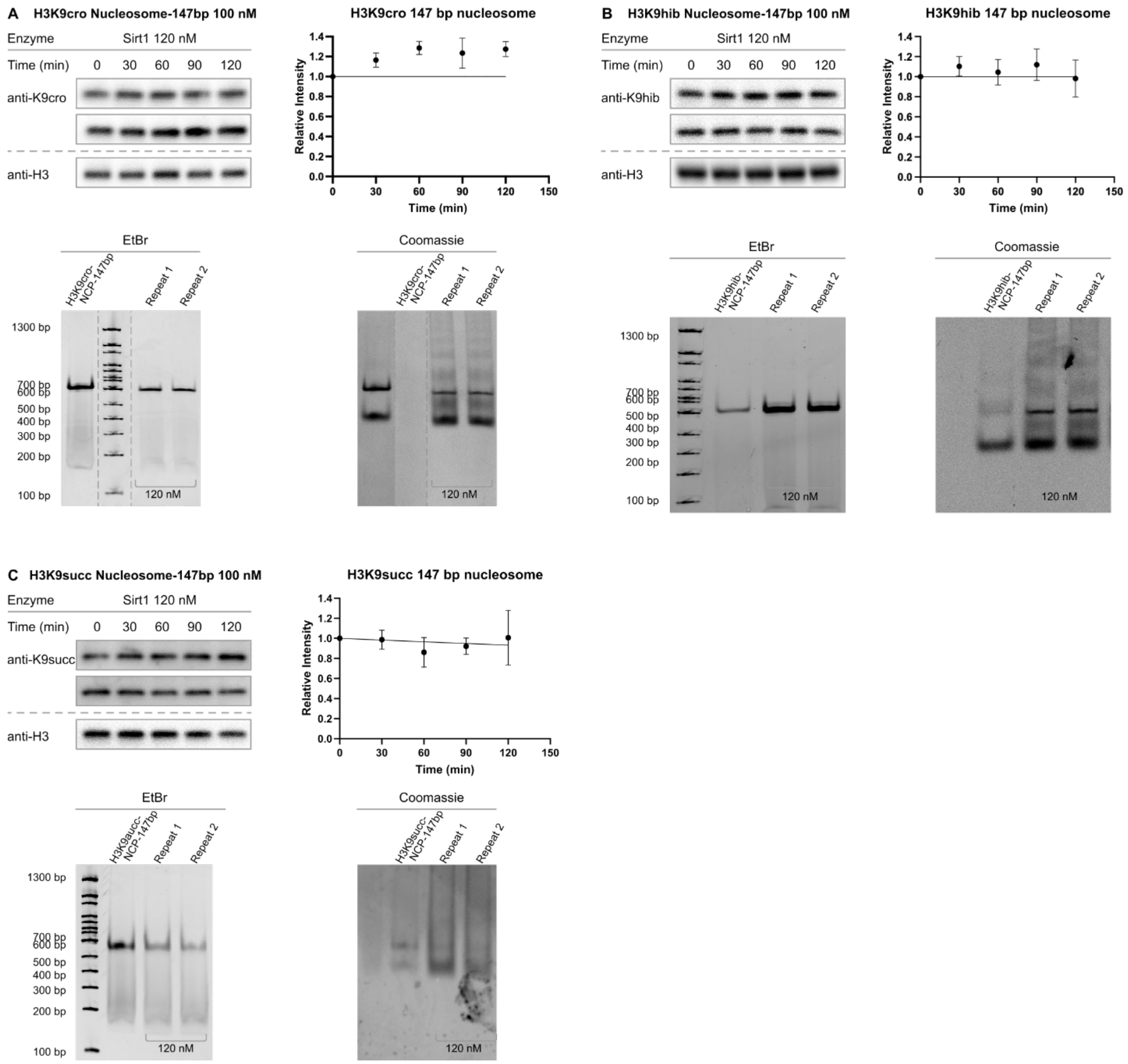

Figure S20. Western blot measurement of Sirt1 activity toward four carbon acylations of H3K9. (**A**) Western blots (top left; anti-H3K9cro; n=2), deacylation fitting (top right; GraphPad Prism 10.2.3; one-phase decay), and native TBE gels (bottom) of Sirt1 deacetylation assays with 147 bp H3K9crotonyl (H3K9cro) nucleosomes. (**B**) Western blots (top left; anti-H3K9hib; n=2), deacylation fitting (top right; GraphPad Prism 10.2.3; one-phase decay), and native TBE gels (bottom) of Sirt1 deacetylation assays with 147 bp H3K9anti-hydroxyisobutyryl nucleosomes. (**C**) Western blots (top left; anti-H3K9succ; n=2), deacylation fitting (top right; GraphPad Prism 10.2.3; one-phase decay), and native TBE gels (bottom) of Sirt1 deacetylation assays with 147 bp H3K9succinyl nucleosomes.

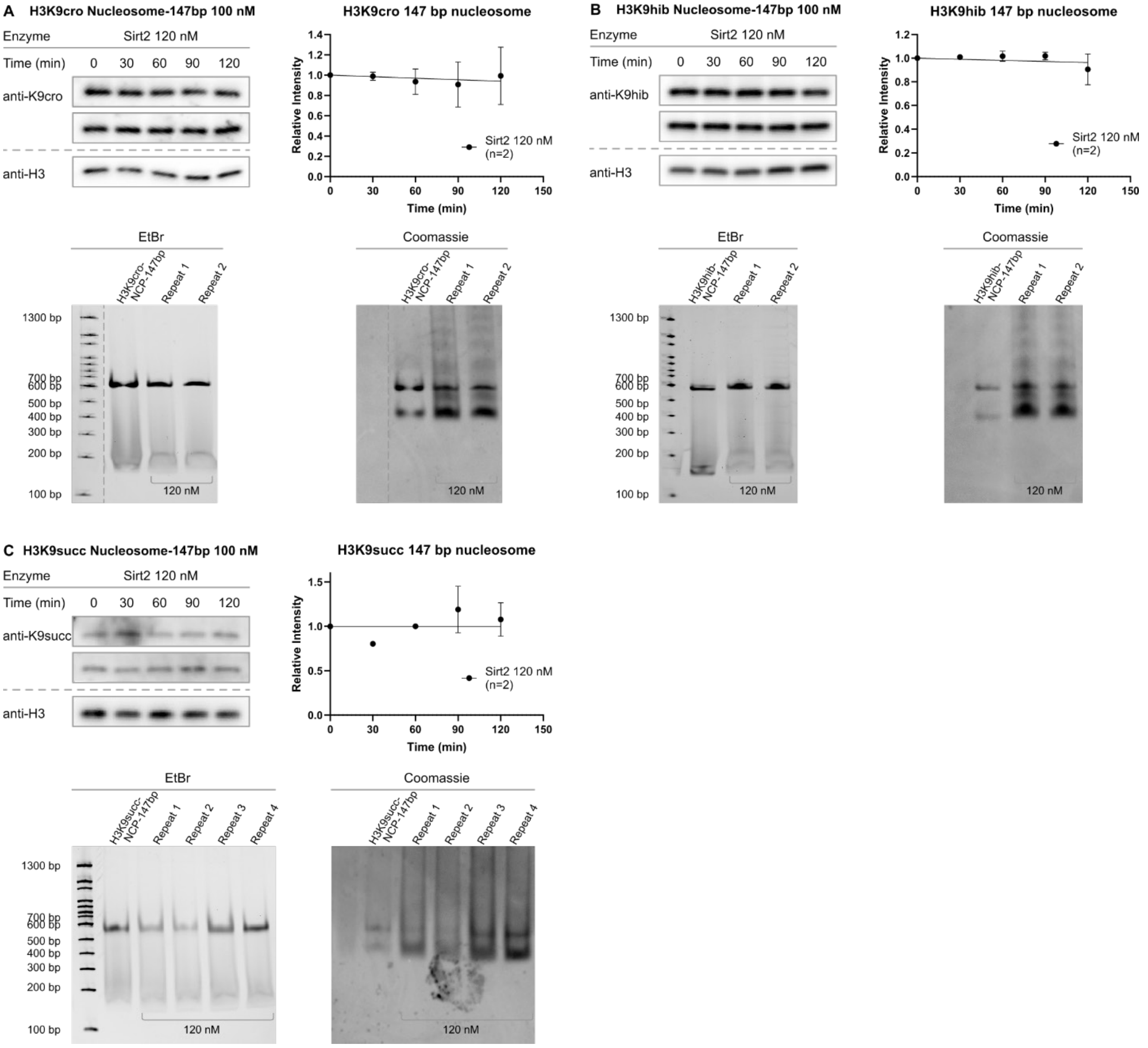

Figure S21. Western blot measurement of Sirt2 activity toward four carbon acylations of H3K9. (**A**) Western blots (top; anti-H3K9cro; n=2), deacylation fitting (right; GraphPad Prism 10.2.3; one-phase decay), and native TBE gels (bottom) of Sirt2 deacetylation assays with 147 bp H3K9crotonyl (H3K9cro) nucleosomes. (**B**) Western blots (top; anti-H3K9hib; n=2), deacylation fitting (right; GraphPad Prism 10.2.3; one-phase decay), and native TBE gels (bottom) of Sirt2 deacetylation assays with 147 bp H3K9anti-hydroxyisobutyryl nucleosomes. (**C**) Western blots (top; anti-H3K9succ; n=2), deacylation fitting (right; GraphPad Prism 10.2.3; one-phase decay), and native TBE gels (bottom) of Sirt6 deacetylation assays with 147 bp H3K9succinyl nucleosomes.

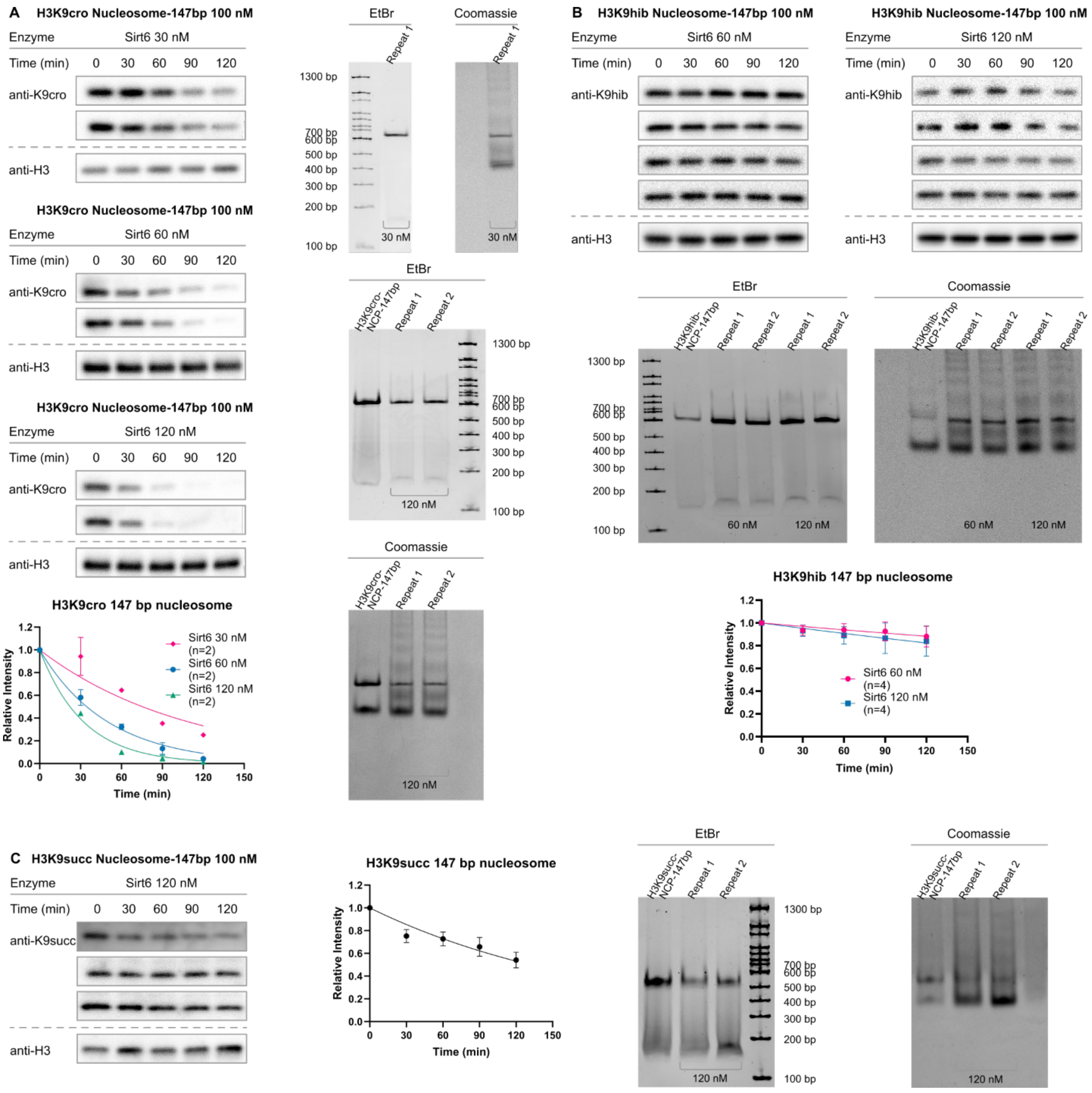

Figure S22. Western blot measurement of Sirt6 activity toward four carbon acylations of H3K9. (**A**) Western blots (top left; anti-H3K9cro; n=6), deacylation fitting (bottom left; GraphPad Prism 10.2.3; one-phase decay), and native TBE gels (right) of Sirt6 deacetylation assays with 147 bp H3K9crotonyl (H3K9cro) nucleosomes. (**B**) Western blots (top; anti-H3K9hib; n=8), deacylation fitting (bottom; GraphPad Prism 10.2.3; one-phase decay), and native TBE gels (middle) of Sirt6 deacetylation assays with 147 bp H3K9anti-hydroxyisobutyryl nucleosomes. (**C**) Western blots (left; anti-H3K9succ; n=3), deacylation fitting (middle; GraphPad Prism 10.2.3; one-phase decay), and native TBE gels (right) of Sirt6 deacetylation assays with 147 bp H3K9succinyl nucleosomes.

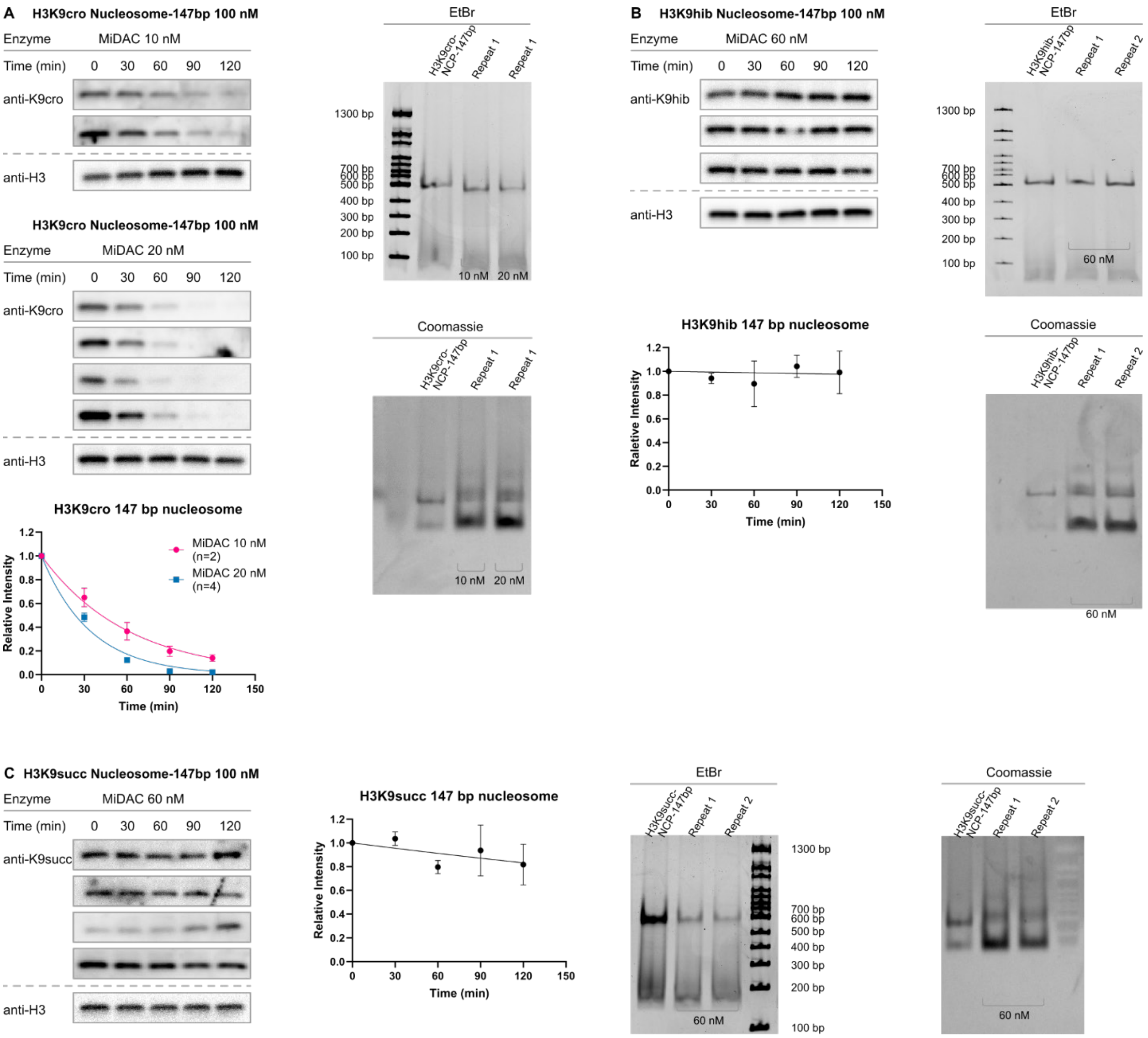

Figure S23. Western blot measurement of MiDAC activity toward four carbon acylations of H3K9. (**A**) Western blots (top left; anti-H3K9cro; n=6), deacylation fitting (bottom left; GraphPad Prism 10.2.3; one-phase decay), and native TBE gels (right) of MiDAC deacetylation assays with 147 bp H3K9crotonyl (H3K9cro) nucleosomes. (**B**) Western blots (top left; anti-H3K9hib; n=3), deacylation fitting (bottom left; GraphPad Prism 10.2.3; one-phase decay), and native TBE gels (bottom) of MiDAC deacetylation assays with 147 bp H3K9anti-hydroxyisobutyryl (H3K9hib) nucleosomes. (**C**) Western blots (left; anti-H3K9succ; n=4), deacylation fitting (middle; GraphPad Prism 10.2.3; one-phase decay), and native TBE gels (right) of MiDAC deacetylation assays with 147 bp H3K9succinyl (H3K9succ) nucleosomes.

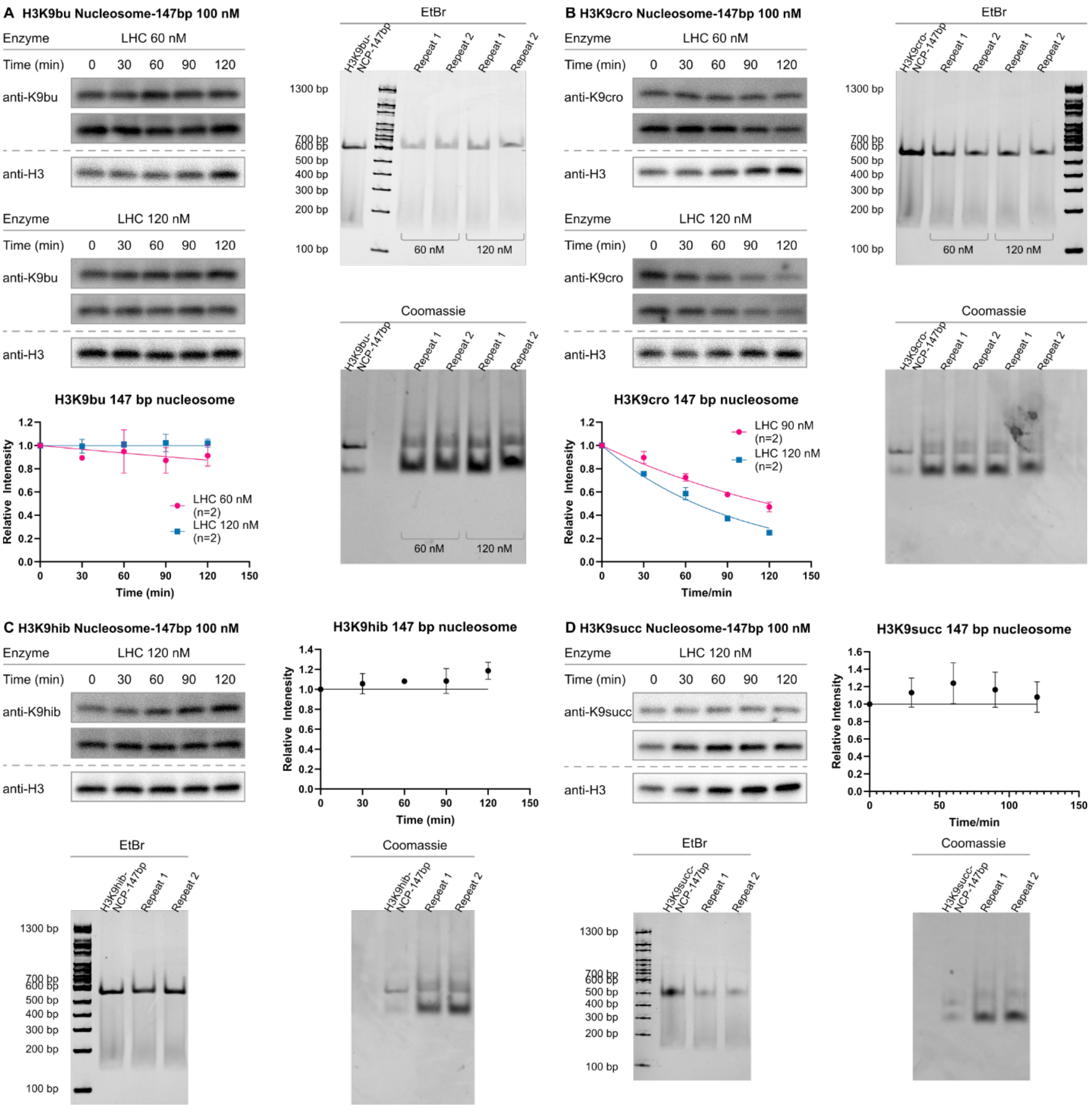

Figure S24. Western blot measurement of LHC activity toward four carbon acylations of H3K9. (**A**) Western blots (top left; anti-H3K9bu; n=4), deacylation fitting (bottom left; GraphPad Prism 10.2.3; one-phase decay), and native TBE gels (right) of LHC deacetylation assays with 147 bp H3K9butyryl (H3K9bu) nucleosomes. (**B**) Western blots (top left; anti-H3K9cro; n=4), deacylation fitting (bottom left; GraphPad Prism 10.2.3; one-phase decay), and native TBE gels (right) of LHC deacetylation assays with 147 bp H3K9crotonyl (H3K9cro) nucleosomes. (**C**) Western blots (top left; anti-H3K9hib; n=2), deacylation fitting (top right; GraphPad Prism 10.2.3; one-phase decay), and native TBE gels (bottom) of LHC deacetylation assays with 147 bp H3K9anti-hydroxyisobutyryl (H3K9hib) nucleosomes. (**D**) Western blots (top left; anti-H3K9succ; n=2), deacylation fitting (top right; GraphPad Prism 10.2.3; one-phase decay), and native TBE gels (bottom) of LHC deacetylation assays with 147 bp H3K9succinyl (H3K9succ) nucleosomes.

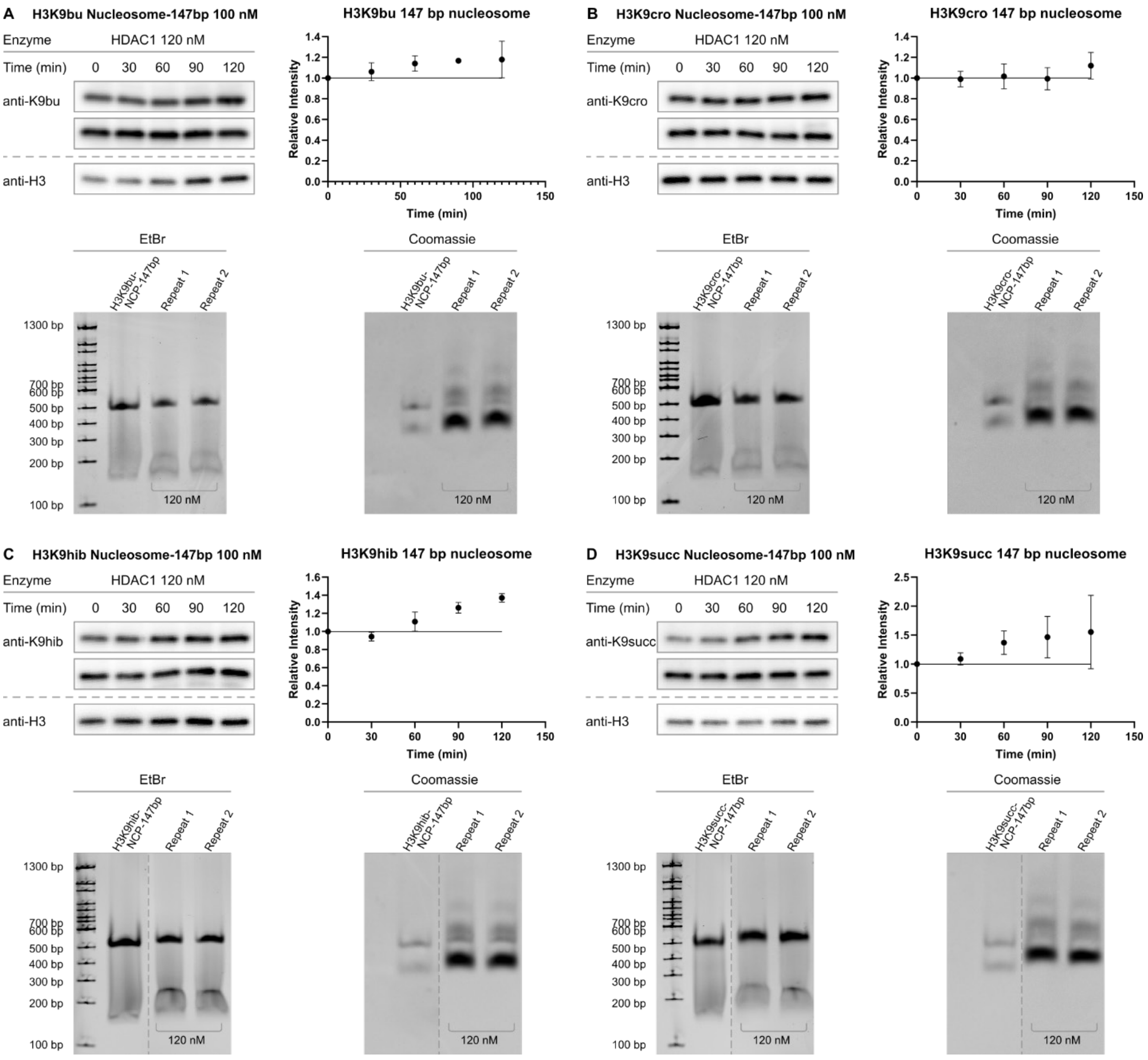

Figure S25. Western blot measurement of free HDAC1 activity toward four carbon acylations of H3K9. (**A**) Western blots (top left; anti-H3K9bu; n=2), deacylation fitting (top right; GraphPad Prism 10.2.3; one-phase decay), and native TBE gels (bottom) of HDAC deacetylation assays with 147 bp H3K9butyryl (H3K9bu) nucleosomes. (**B**) Western blots (top left; anti-H3K9cro; n=2), deacylation fitting (top right; GraphPad Prism 10.2.3; one-phase decay), and native TBE gels (bottom) of HDAC deacetylation assays with 147 bp H3K9crotonyl (H3K9cro) nucleosomes. (**C**) Western blots (top left; anti-H3K9hib; n=2), deacylation fitting (top right; GraphPad Prism 10.2.3; one-phase decay), and native TBE gels (bottom) of HDAC deacetylation assays with 147 bp H3K9anti-hydroxyisobutyryl (H3K9hib) nucleosomes. (**D**) Western blots (top left; anti-H3K9hib; n=2), deacylation fitting (top right; GraphPad Prism 10.2.3; one-phase decay), and native TBE gels (bottom) of HDAC deacetylation assays with 147 bp H3K9succinyl (H3K9succ) nucleosomes.

Acylated Nucleosome	Sirt 1	Sirt2	Sirt6	MiDAC	LHC	HDAC1
H3K9ac	<0.002	0.026±0.0015*	0.075±0.0044	1.4±0.046	0.021±0.0011	<0.002*
H3K9bu	<0.002	0.013±0.00050	0.044±0.0049	0.0077±0.00053	<0.002	<0.002
H3K9cro	<0.002	<0.002	0.033±0.0021	0.16±0.011	0.0086±0.00039	<0.002
H3K9hib	<0.002	<0.002	0.0013±0.00049	<0.002	<0.002	<0.002
H3K9succ	<0.002	<0.002	0.0044±0.00050	<0.002	<0.002	<0.002

Table S7. Calculated V/[E] values for HDAC activity toward four carbon acylations of H3K9. Average V/[E] ± SEM values for Sirt1, Sirt2, Sirt6, MiDAC, LHC and free HDAC1 deacylation of 147 bp nucleosomes with different 4 carbon acylations. Western blot bands were quantified by ImageJ (imagej.nih.gov/ij/). Intensity values were normalized to intensity at t=0 and then fit to a single-phase exponential decay curve with constrain Y0=1, Plateau=0 (GraphPad Prism 10). Values marked with asterisks are re-printed from prior reports.[20,21]

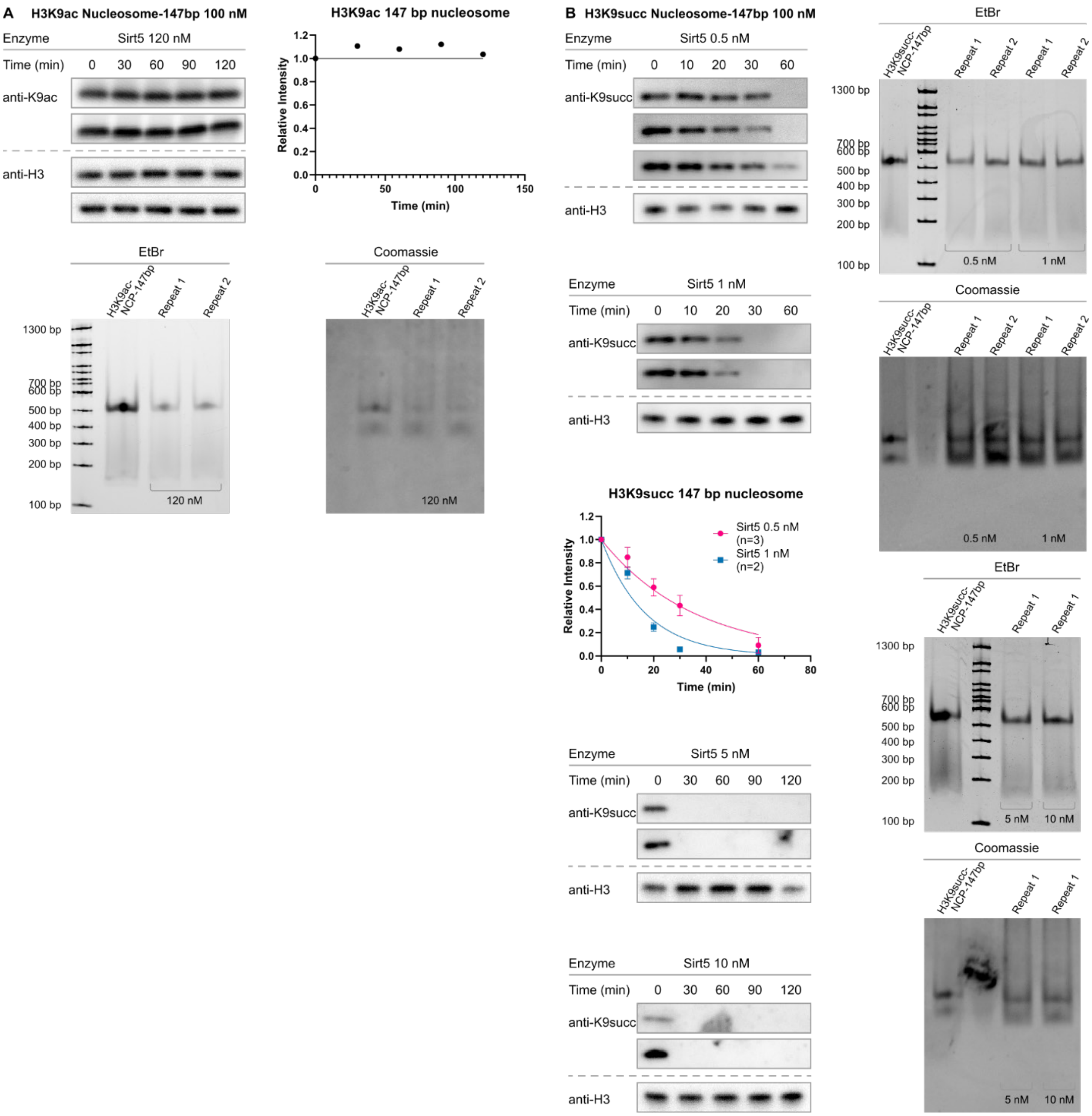

Figure S26. Western blot measurement of Sirtuin5 activity toward acetylated and succinylated H3K9. (**A**) Western blots (top; anti-H3K9ac; n=2), deacylation fitting (right; GraphPad Prism 10.2.3; one-phase decay), and native TBE gels (bottom) of Sirt5 deacetylation assays with 147 bp H3K9acetul (H3K9ac) nucleosomes. (**B**) Western blots (top left; anti-H3K9succ; n=5), deacylation fitting (center left; GraphPad Prism 10.2.3; one-phase decay), and native TBE gels (right) of Sirt5 deacetylation assays with 147 bp H3K9succinyl (H3K9succ) nucleosomes. Deacylation with 5 and 10 nM enzyme (bottom left) was too fast to fit, and is not included in subsequent V/[E] calculation.

Acylated Nucleosome	Sirt 1	Sirt2	Sirt6	MiDAC	LHC	HDAC1	Sirt5
H3K9ac	<0.002	0.026±0.0015*	0.075±0.0044	1.4±0.046	0.021±0.0011	<0.002*	<0.002
H3K9succ	<0.002	<0.002	0.0044±0.00050	<0.002	<0.002	<0.002	5.7±0.64

Table S8. Calculated V/[E] values for HDAC activity toward H3K9 succinylation. Average V/[E] ± SEM values for Sirt1, Sirt2, Sirt6, MiDAC, LHC, free HDAC1 and Sirt5 deacylation of 147 bp nucleosomes with H3K9 succinylation. Western blot bands were quantified by ImageJ (imagej.nih.gov/ij/). Intensity values were normalized to intensity at t=0 and then fit to a single-phase exponential decay curve with constrain Y0=1, Plateau=0 (GraphPad Prism 10). Values marked with asterisks are re-printed from prior reports.[20,21]

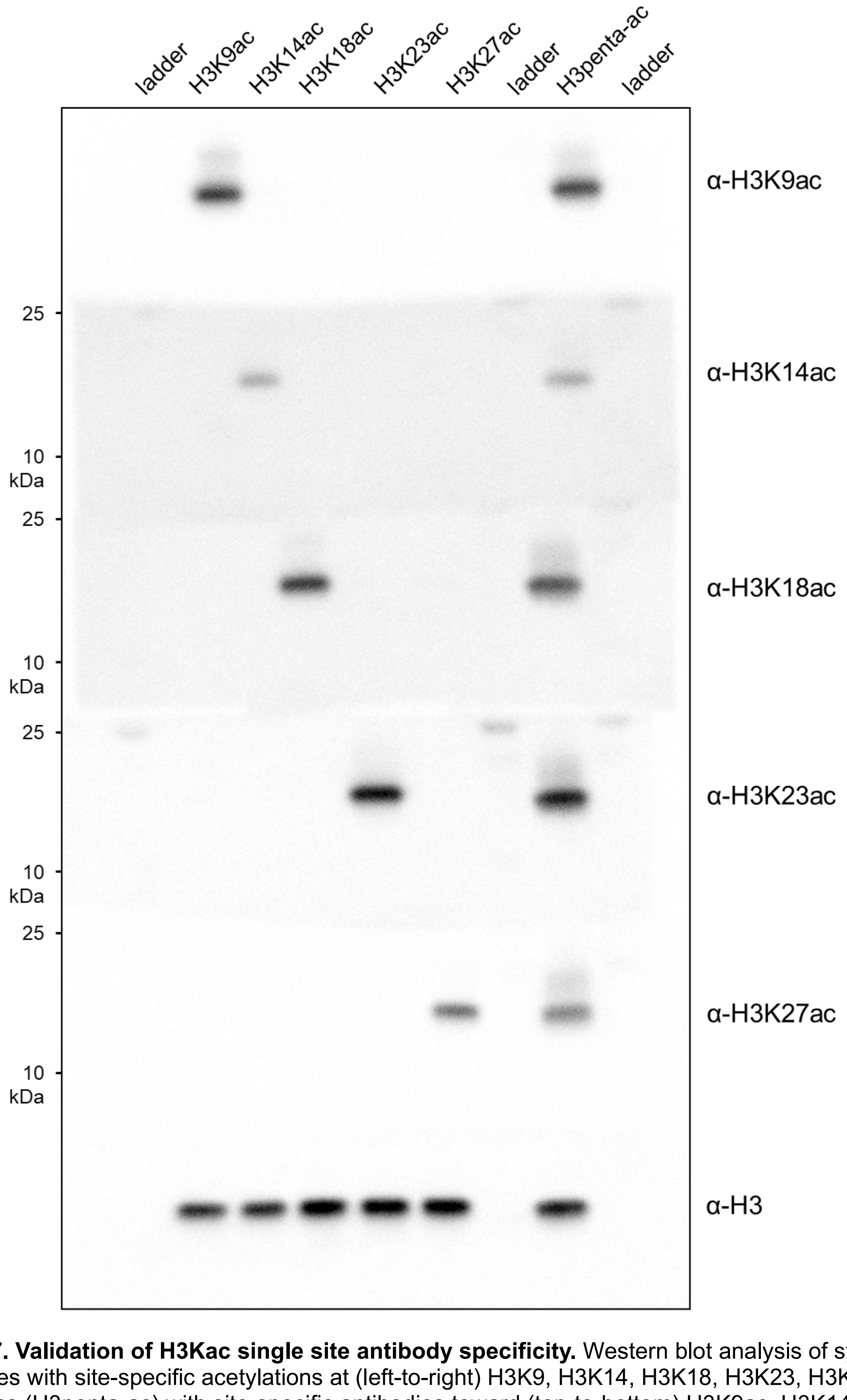

Figure S27. Validation of H3Kac single site antibody specificity. Western blot analysis of synthetic nucleosomes with site-specific acetylations at (left-to-right) H3K9, H3K14, H3K18, H3K23, H3K27, or all five positions (H3penta-ac) with site-specific antibodies toward (top-to-bottom) H3K9ac, H3K14ac, H3K18ac, H3K23ac or H3K27ac. Molecular weight markers are visible in blots for H3K14ac, H3K18ac, H3K23ac and H3K27ac.

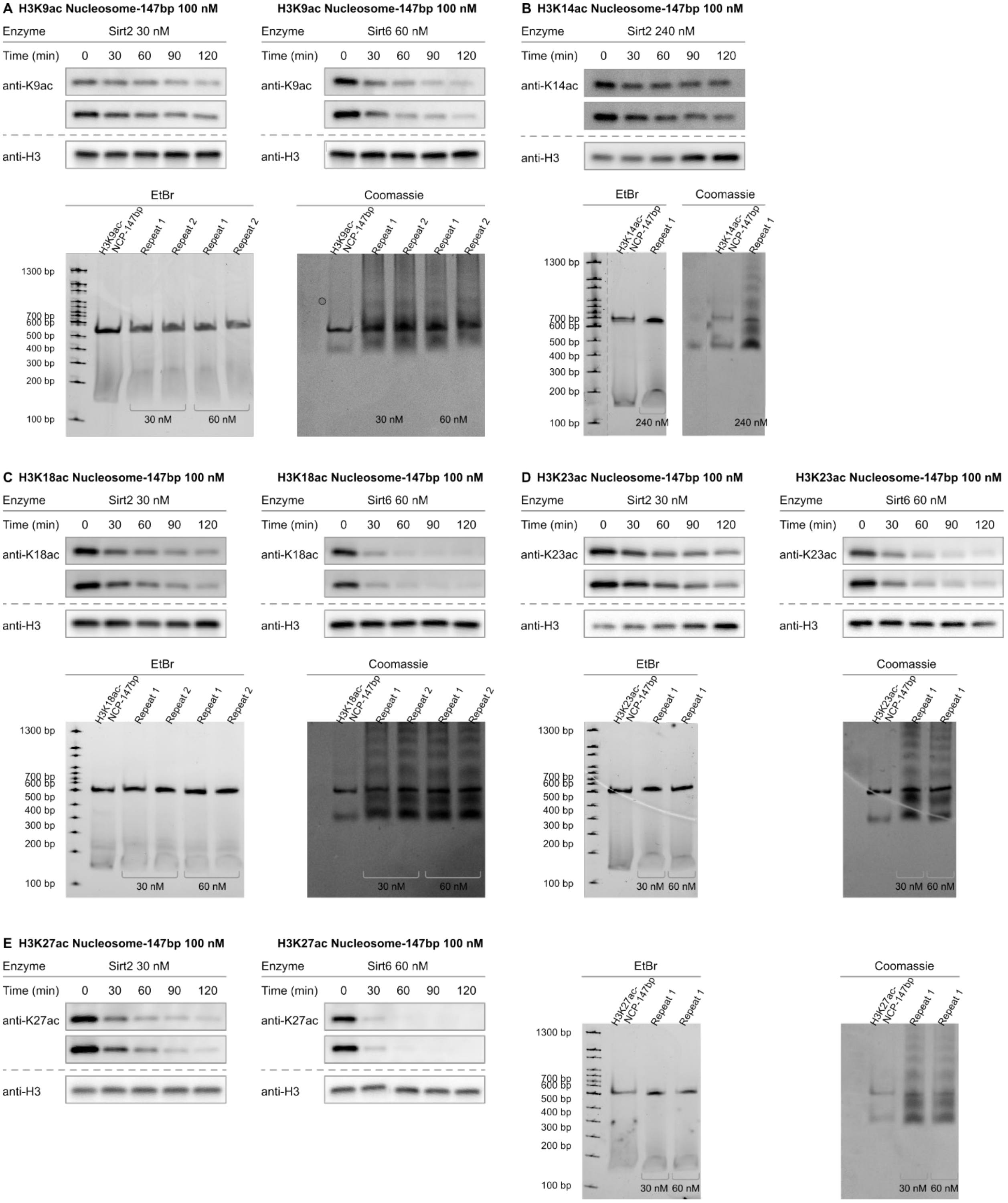

Figure S28. Western blot measurement of Sirt2 activity toward mono-acetylated nucleosomes. (**A**) Western blots (top; n=4) and native TBE gels (bottom) of Sirt2 deacetylation assays with 147 bp H3K9acetyl (H3K9ac) nucleosomes. (**B**) Western blots (top; n=2) and native TBE gels (bottom) of Sirt2 deacetylation assays with 147 bp H3K14acetyl (H3K14ac) nucleosomes. (**C**) Western blots (top; n=4) and native TBE gels (bottom) of Sirt2 deacetylation assays with 147 bp H3K18acetyl (H3K8ac) nucleosomes. (**D**) Western blots (top; n=4) and native TBE gels (bottom) of Sirt2 deacetylation assays with 147 bp

H3K13acetyl (H3K13ac) nucleosomes. (**A**) Western blots (left; n=4) and native TBE gels (right) of Sirt2 deacetylation assays with 147 bp H3K27acetyl (H3K27ac) nucleosomes.

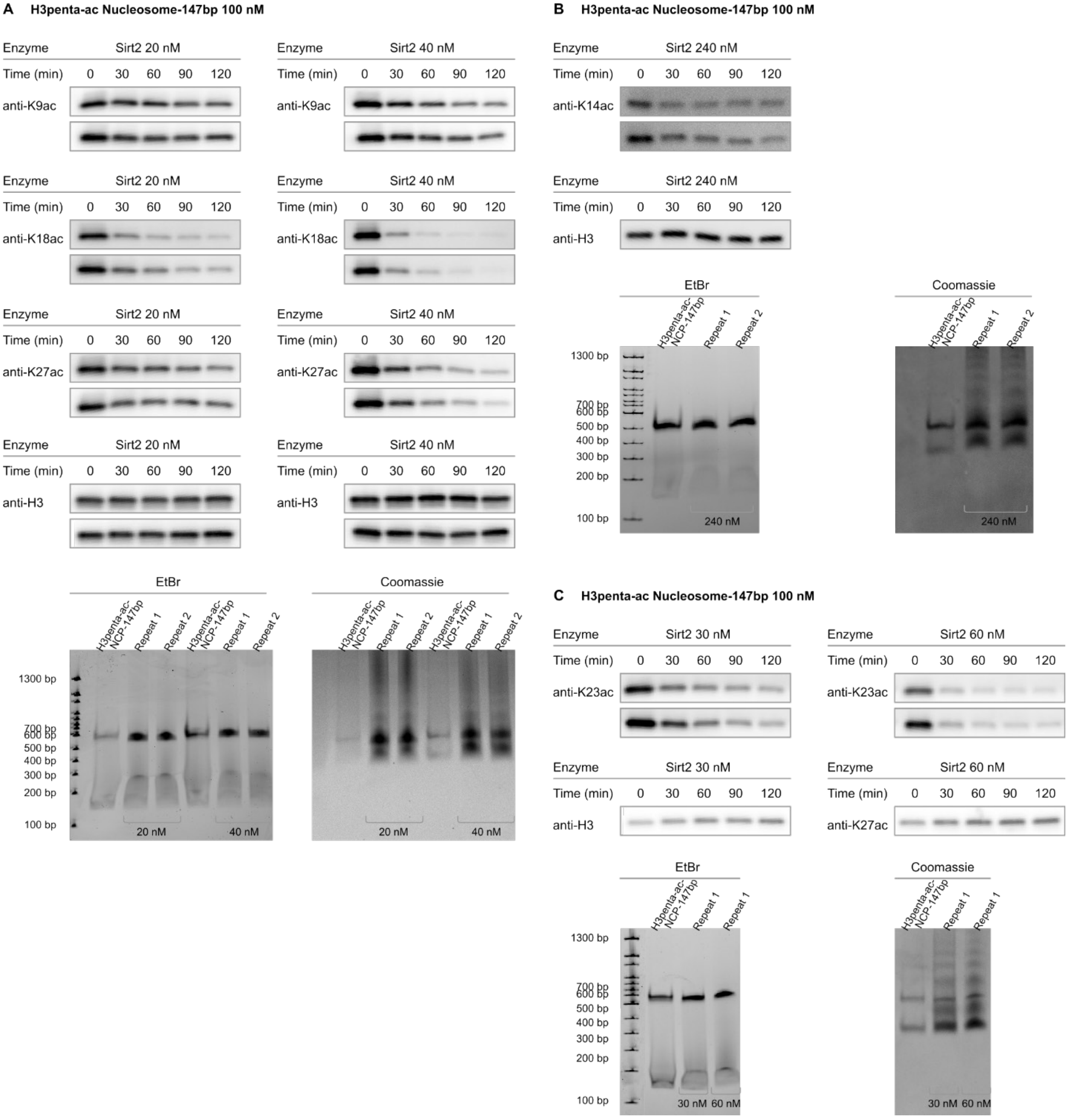

Figure S29. Western blot measurement of Sirt2 activity toward penta-acetylated nucleosomes. Western blots (top; anti-H3K9ac, anti-H3K18ac, anti-H3K27ac; n=4) and native TBE gels (bottom) of Sirt2 deacetylation assays with 147 bp H3K9ac/K14ac/K18ac/K23ac/K27ac (H3Kpenta-ac) nucleosomes. (**B**) Western blots (top; anti-H3K14ac; n=2) and native TBE gels (bottom) of Sirt2 deacetylation assays with 147 bp H3K9ac/K14ac/K18ac/K23ac/K27ac (H3Kpenta-ac) nucleosomes. (**C**) Western blots (top; anti-H3K23ac, anti-H3K23ac; n=4) and native TBE gels (bottom) of Sirt2 deacetylation assays with 147 bp H3K9ac/K14ac/K18ac/K23ac/K27ac (H3Kpenta-ac) nucleosomes.

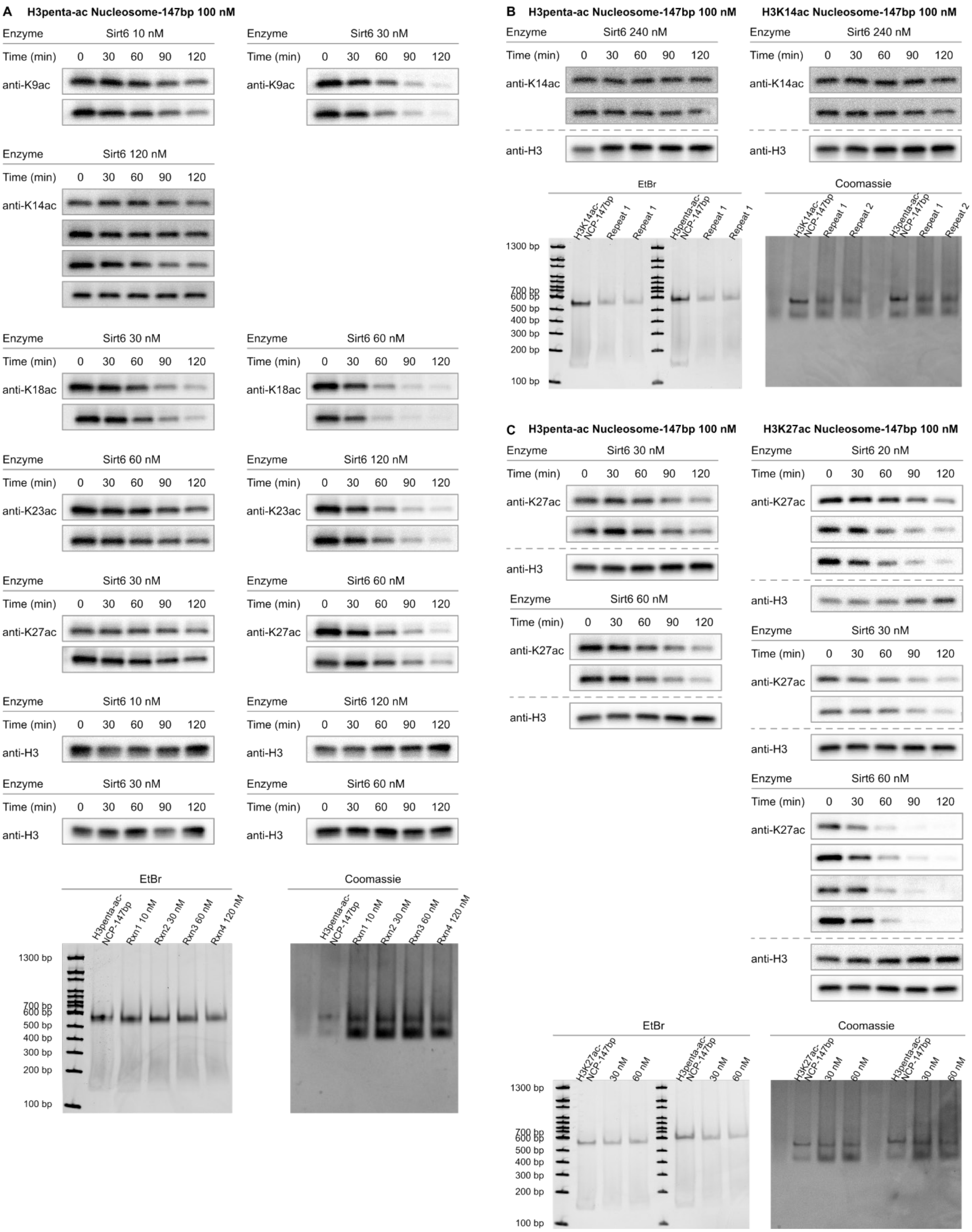

Figure S30. Western blot measurement of Sirt6 activity toward mono- and penta-acetylated nucleosomes. (**A**) Western blots (top; anti-H3K9ac, anti-H3K14ac, anti-H3K18ac, anti-H3K23ac, anti-H3K27ac; n=4) and native TBE gels (bottom) of Sirt6 deacetylation assays with 147 bp

H3K9ac/K14ac/K18ac/K23ac/K27ac (H3Kpenta-ac) nucleosomes. (**B**) Western blots (top; anti-H3K14ac) and native TBE gels (bottom) of parallel Sirt6 deacetylation assays with 147 bp H3K9ac/K14ac/K18ac/K23ac/K27ac (H3Kpenta-ac) nucleosomes (top left; n=2) and 147 bp H3K14acetyl (H3K14ac) nucleosomes (top right; n=2). (**C**) Western blots (top; anti-H3K27ac) and native TBE gels (bottom) of parallel Sirt6 deacetylation assays with 147 bp H3K9ac/K14ac/K18ac/K23ac/K27ac (H3Kpenta-ac) nucleosomes (top left; n=4) and 147 bp H3K27acetyl (H3K27ac) nucleosomes (top right; n=9).

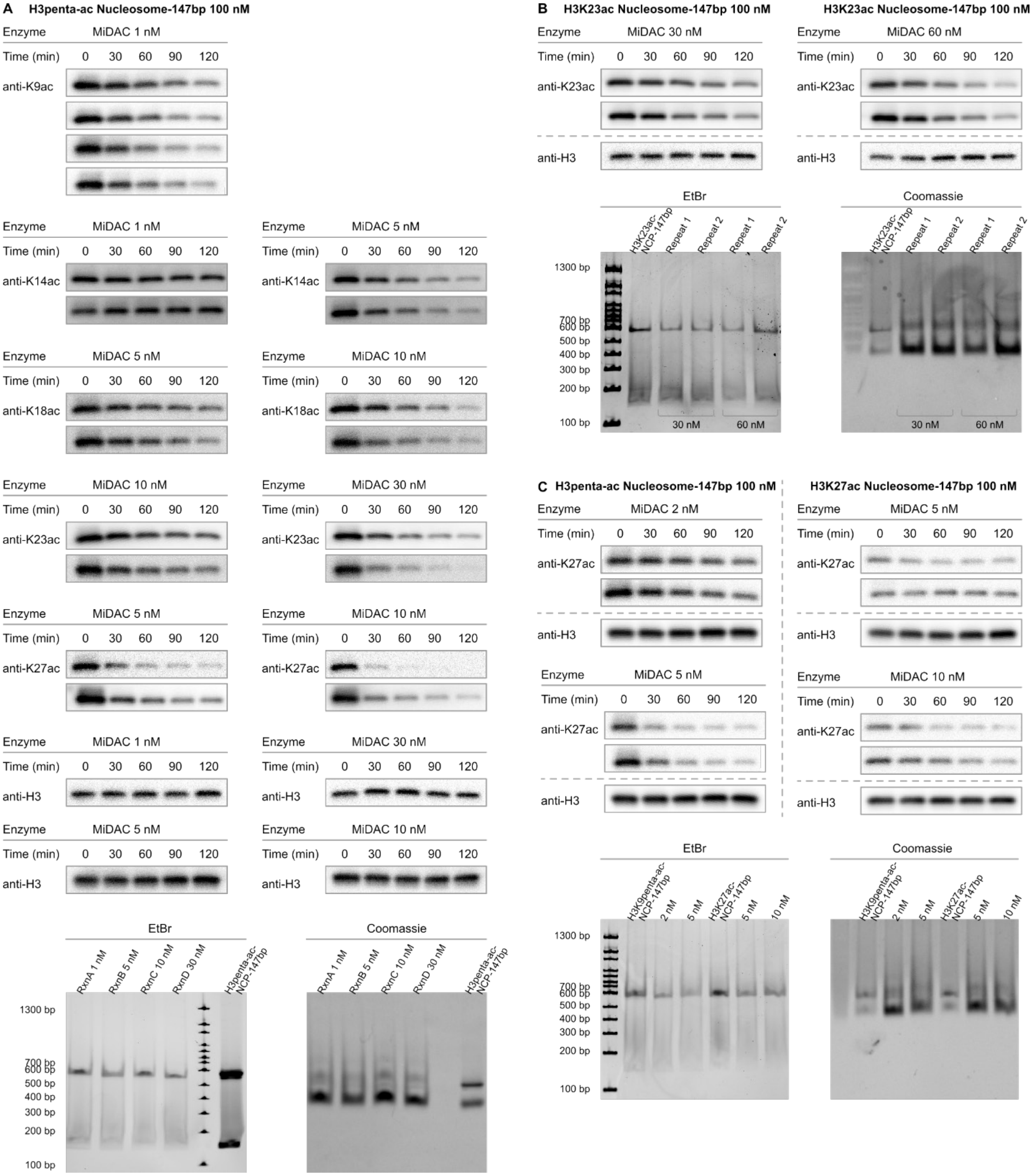

Figure S31. Western blot measurement of MiDAC activity toward mono- and penta-acetylated nucleosomes. (**A**) Western blots (top; anti-H3K9ac, anti-H3K14ac, anti-H3K18ac, anti-H3K23ac, anti-H3K27ac) and native TBE gels (bottom) of MiDAC deacetylation assays with 147 bp H3K9ac/K14ac/K18ac/K23ac/K27ac (H3Kpenta-ac) nucleosomes (n=4). (**B**) Western blots (top; anti-H3K23ac) and native TBE gels (bottom) of MiDAC deacetylation assays with 147 bp H3K23acetyl (H3K23ac) nucleosomes (n=4). (**C**) Western blots (top; anti-H3K23ac) and native TBE gels (bottom) of MiDAC deacetylation assays with 147 bp H3K9ac/K14ac/K18ac/K23ac/K27ac (H3Kpenta-ac) nucleosomes (top left; n=4) and 147 bp H3K27acetyl (H3K27ac) nucleosomes (top right; n=4).

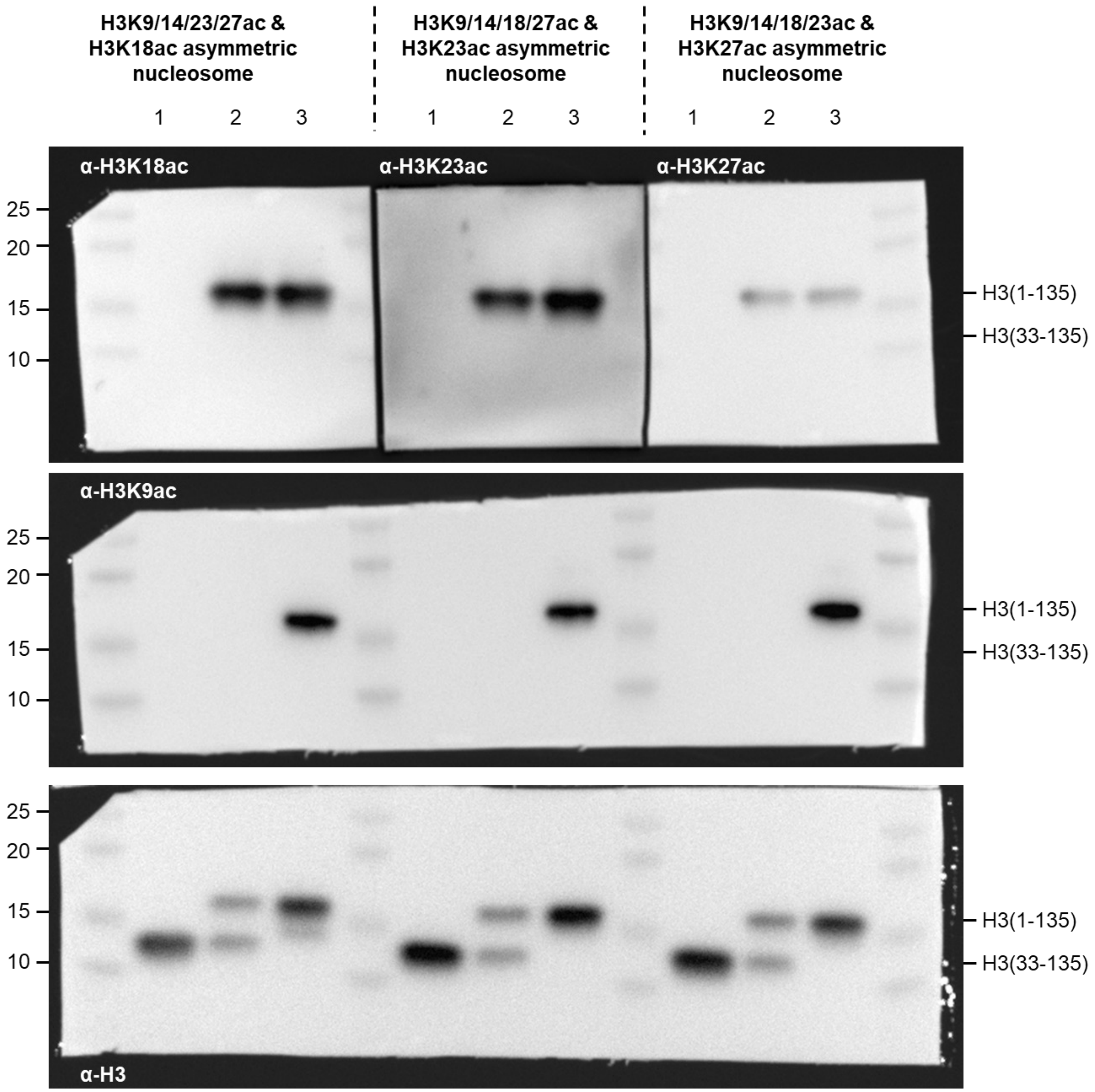

Figure S32. Western blot characterization of asymmetric mono/tetra-acetylated nucleosomes. Anti-H3 (bottom), anti-H3K9ac (middle) and either anti-H3K18ac (top left), anti-H3K23ac (top center), or anti-H3K27ac (top right). Lanes: 1 – tailless starting material; 2 – intermediate single H3 tail product; 3 – final asymmetric mono-acetylated / tetra-acetylated nucleosomes.

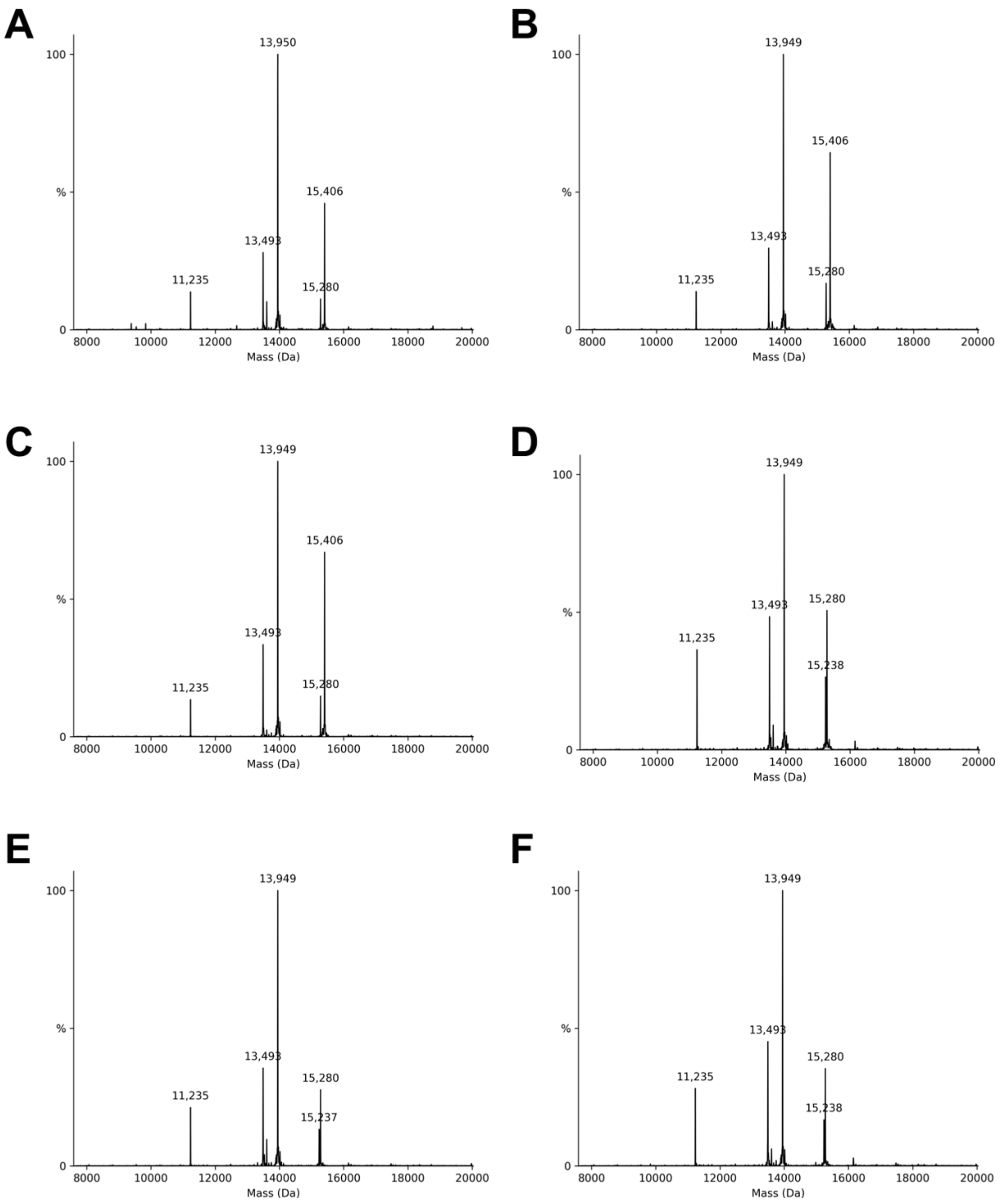

Figure S33. Mass spectrometric characterization of asymmetric mono/tetra-acetylated nucleosomes and asymmetric mono-acetylated/unmodified nucleosomes. Deconvoluted mass spectra of (**A**) asymmetric H3K18ac & H3K9/14/23/27ac nucleosome, (**B**) asymmetric H3K23ac & H3K9/14/18/27ac nucleosome, and (**C**) asymmetric H3K27ac & H3K9/14/18/23ac nucleosome: Mono-acetyl H3 calc'd for $C_{672}H_{1133}N_{215}O_{187}S_2$ $[M]^+$: 15280.64 Da, Found: 15280 Da; Tetra-acetyl H3 calc'd for $C_{678}H_{1139}N_{215}O_{190}S_2$ $[M]^+$: 15406.75 Da, Found: 15406 Da. Deconvoluted mass spectra of (**D**) asymmetric H3K18ac & unmodified H3 nucleosome, (**E**) asymmetric H3K27ac & unmodified H3 nucleosome, and (**F**) asymmetric H3K23ac & unmodified H3 nucleosome: Mono-acetyl H3 calc'd for $C_{672}H_{1133}N_{215}O_{187}S_2$ [M]+: 15280.64 Da, Found: 15280 Da; unmodified H3 Calc'd for $C_{670}H_{1131}N_{215}O_{186}S_2$ $[M]^+$: 15238.61 Da, Found: 15238 Da. Raw spectra deconvoluted with UniDec.[19]

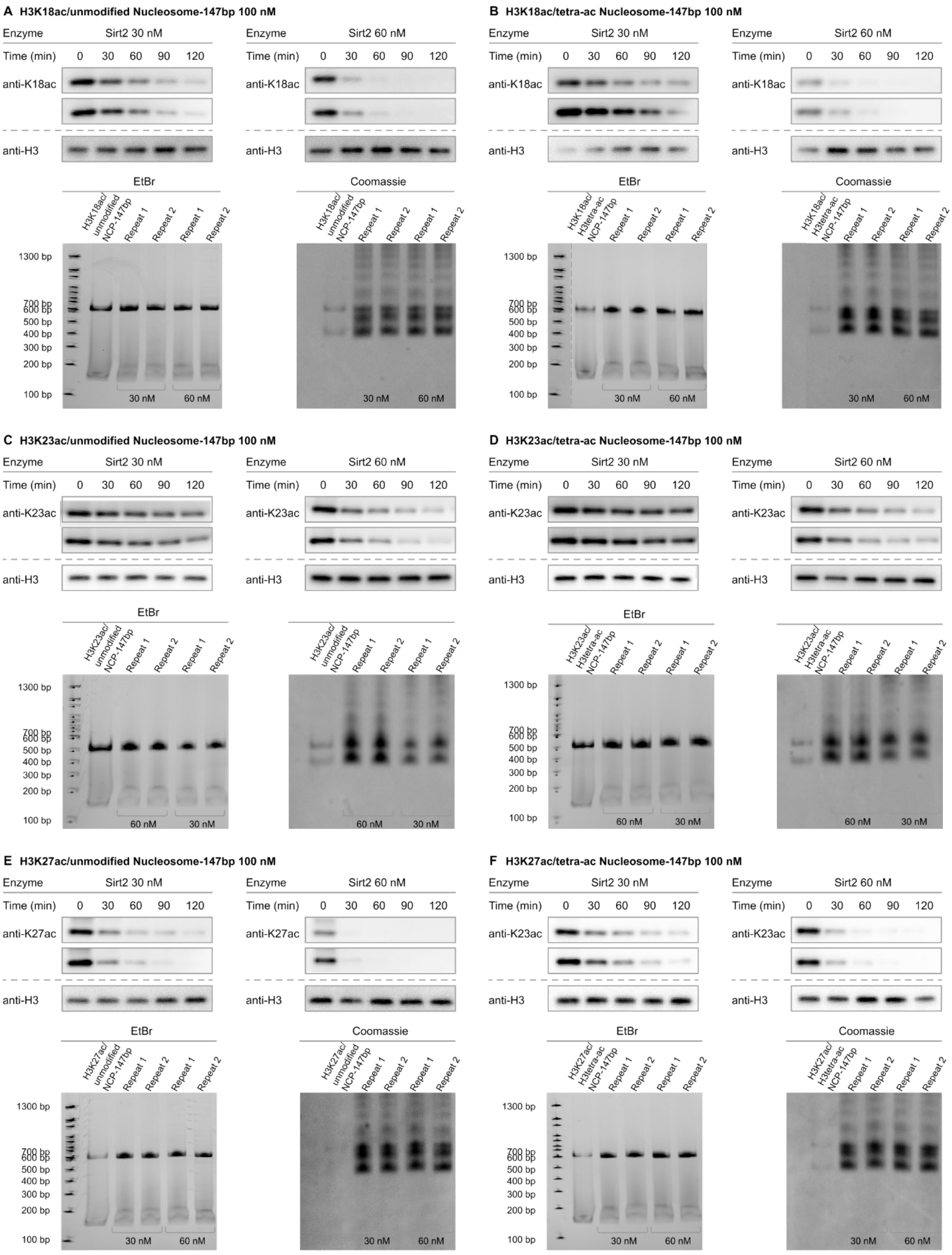
A H3K18ac/unmodified Nucleosome-147bp 100 nM
Enzyme Sirt2 30 nM
Time (min) 0 30 60 90 120
anti-K18ac
anti-H3
Enzyme Sirt2 60 nM
Time (min) 0 30 60 90 120
anti-K18ac
anti-H3
EtBr
H3K18ac/ unmodified NCP-147bp
Repeat 1
Repeat 2
Repeat 1
Repeat 2
1300 bp
700 bp
600 bp
500 bp
400 bp
300 bp
200 bp
100 bp
30 nM
60 nM
Coomassie
B H3K18ac/tetra-ac Nucleosome-147bp 100 nM
anti-K18ac
anti-H3
H3K18ac/ H3tetra-ac NCP-147bp
C H3K23ac/unmodified Nucleosome-147bp 100 nM
anti-K23ac
anti-H3
H3K23ac/ unmodified NCP-147bp
60 nM
30 nM
D H3K23ac/tetra-ac Nucleosome-147bp 100 nM
anti-K23ac
anti-H3
H3K23ac/ H3tetra-ac NCP-147bp
E H3K27ac/unmodified Nucleosome-147bp 100 nM
anti-K27ac
anti-H3
H3K27ac/ unmodified NCP-147bp
F H3K27ac/tetra-ac Nucleosome-147bp 100 nM
anti-K23ac
anti-H3
H3K27ac/ H3tetra-ac NCP-147bp

Figure S34. Western blot measurement of Sirt2 activity toward asymmetrically acetylated nucleosomes. (**A**) Western blots (top; anti-H3K18ac; n=4) and native TBE gels (bottom) of Sirt2 deacetylation assays with 147 bp asymmetric unmodified H3 & H3K18ac nucleosomes. (**B**) Western blots (top; anti-H3K18ac; n=4) and native TBE gels (bottom) of Sirt2 deacetylation assays with 147 bp asymmetric H3K9ac/K14ac/K23ac/K27ac (H3tetra-ac) & H3K18ac nucleosomes. (**C**) Western blots (top; anti-H3K23ac; n=4) and native TBE gels (bottom) of Sirt2 deacetylation assays with 147 bp asymmetric unmodified H3 & H3K23ac nucleosomes. (**D**) Western blots (top; anti-H3K23ac; n=4) and native TBE gels (bottom) of Sirt2 deacetylation assays with 147 bp asymmetric H3K9ac/K14ac/K18ac/K27ac (H3tetra-ac) & H3K23ac nucleosomes. (**E**) Western blots (top; anti-H3K27ac; n=4) and native TBE gels (bottom) of Sirt2 deacetylation assays with 147 bp asymmetric unmodified H3 & H3K27ac nucleosomes. (**F**) Western blots (top; anti-H3K27ac; n=4) and native TBE gels (bottom) of Sirt2 deacetylation assays with 147 bp asymmetric H3K9ac/K14ac/K18ac/K23ac (H3tetra-ac) & H3K27ac nucleosomes.

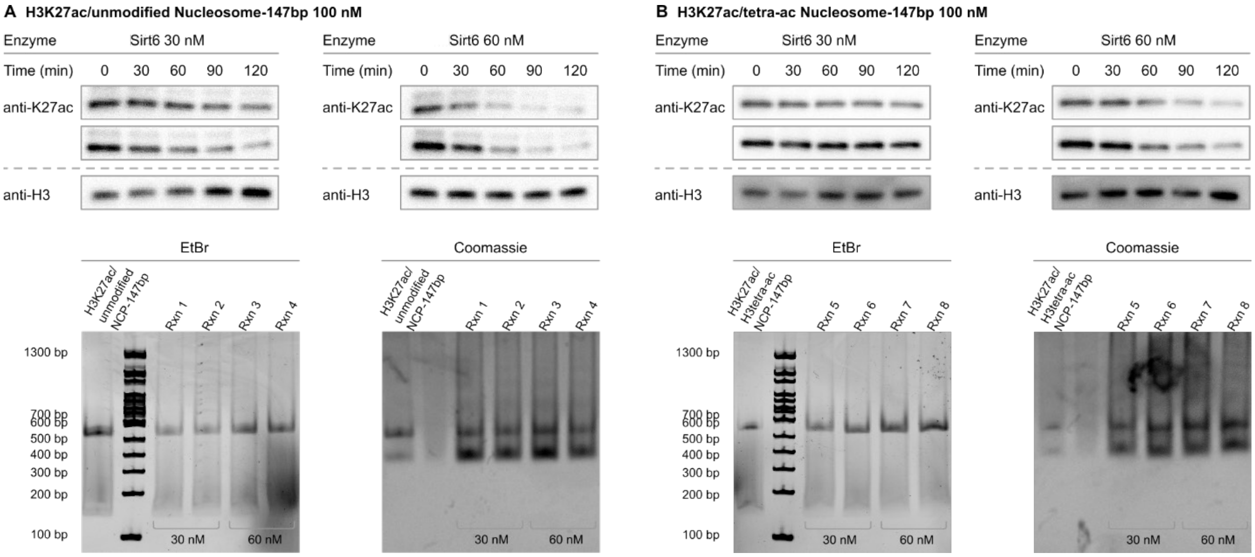

Figure S35. Western blot measurement of Sirt6 activity toward asymmetrically acetylated nucleosomes. (**A**) Western blots (top; anti-H3K27ac; n=4) and native TBE gels (bottom) of Sirt6 deacetylation assays with 147 bp asymmetric unmodified H3 & H3K27ac nucleosomes. (**B**) Western blots (top; anti-H3K27ac; n=4) and native TBE gels (bottom) of Sirt6 deacetylation assays with 147 bp asymmetric H3K9ac/K14ac/K18ac/K23ac (H3tetra-ac) & H3K27ac nucleosomes.

Sirt2			Sirt2		
Symmetric Nucleosome V/[E] (min-1)	Mono-acetylated	Penta-acetylated	Asymmetric Nucleosome V/[E] (min-1)	mono-acetylated / unmodified	mono-acetylated / tetra-acetylated
H3K9ac	0.038±0.0021*	0.031±0.0024	H3K9ac	(-)	(-)
H3K14ac	0.0034±0.0003	0.0051±0.0005	H3K14ac	(-)	(-)
H3K18ac	0.056±0.0028	0.11±0.0096	H3K18ac	0.058±0.0025	0.043±0.0033
H3K23ac	0.031±0.0011	0.056±0.0033	H3K23ac	0.034±0.0016	0.027±0.0014
H3K27ac	0.12±0.0071*	0.051±0.0038	H3K27ac	0.12±0.0064	0.071±0.0021

Table S9. Calculated V/[E] values for Sirt2 activity toward mono-, penta-, and asymmetrically acetylated nucleosomes. Average V/[E] ± SEM values. Values marked with asterisks are re-printed from prior reports.[20]

Sirt6			Sirt6		
Symmetric Nucleosome V/[E] (min-1)	Mono-acetylated	Penta-acetylated	Asymmetric Nucleosome V/[E] (min-1)	mono-acetylated / unmodified	mono-acetylated / tetra-acetylated
H3K9ac	0.075±0.0044	0.072±0.0085	H3K9ac	(-)	(-)
H3K14ac	0.00080±0.00024	0.0013±0.00023	H3K14ac	(-)	(-)
H3K18ac	0.043±0.0031*	0.038±0.0033	H3K18ac	(-)	(-)
H3K23ac	0.011±0.00059*	0.014±0.0011	H3K23ac	(-)	(-)
H3K27ac	0.033±0.0029	0.019±0.0016	H3K27ac	0.036±0.0021	0.020±0.0018

Table S10. Calculated V/[E] values for Sirt6 activity toward mono-, penta-, and asymmetrically acetylated nucleosomes. Average V/[E] ± SEM values. Values marked with asterisks are re-printed from prior reports.[7]

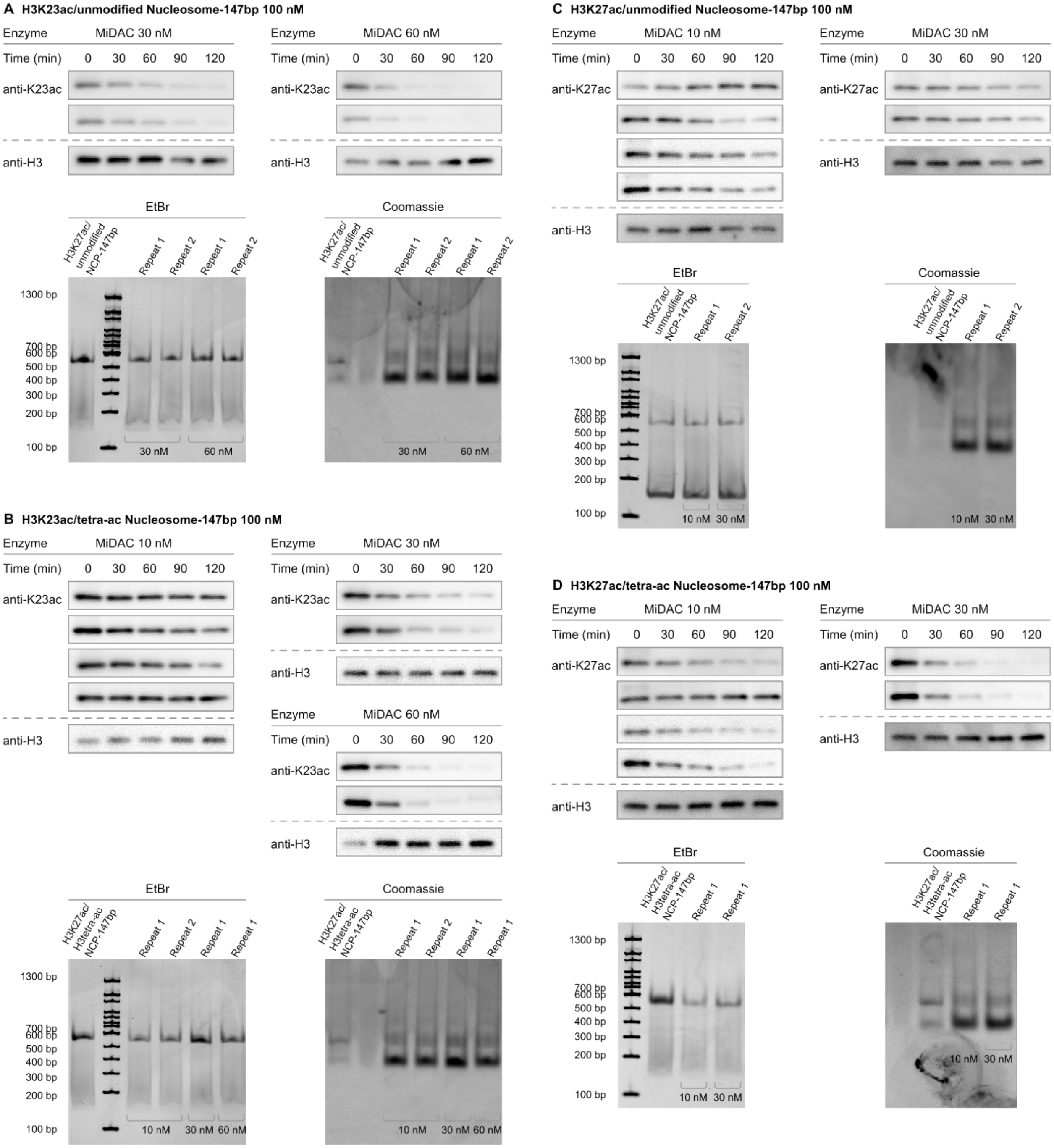

Figure S36. Western blot measurement of MiDAC activity toward asymmetrically acetylated nucleosomes. (**A**) Western blots (top; anti-H3K23ac; n=4) and native TBE gels (bottom) of MiDAC deacetylation assays with 147 bp asymmetric unmodified H3 & H3K23ac nucleosomes. (**B**) Western blots (top; anti-H3K23ac; n=8) and native TBE gels (bottom) of MiDAC deacetylation assays with 147 bp asymmetric H3K9ac/K14ac/K18ac/K27ac (H3tetra-ac) & H3K23ac nucleosomes. (**C**) Western blots (top; anti-H3K27ac; n=6) and native TBE gels (bottom) of MiDAC deacetylation assays with 147 bp asymmetric unmodified H3 & H3K27ac nucleosomes. (**D**) Western blots (top; anti-H3K27ac; n=6) and native TBE gels (bottom) of MiDAC deacetylation assays with 147 bp asymmetric H3K9ac/K14ac/K18ac/K23ac (H3tetra-ac) & H3K27ac nucleosomes.

MiDAC			MiDAC		
Symmetric Nucleosome V/[E] (min-1)	Mono-acetylated	Penta-acetylated	Asymmetric Nucleosome V/[E] (min-1)	mono-acetylated / unmodified	mono-acetylated / tetra-acetylated
H3K9ac	1.4±0.046	1.4±0.058	H3K9ac	(-)	(-)
H3K14ac	0.52 ± 0.034*	0.39±0.020	H3K14ac	(-)	(-)
H3K18ac	0.17 ± 0.0051*	0.18±0.013	H3K18ac	(-)	(-)
H3K23ac	0.021±0.00063	0.11±0.0082	H3K23ac	0.065±0.0022	0.073±0.0025
H3K27ac	0.11±0.0094	0.40±0.034	H3K27ac	0.030±0.00073	0.12±0.0017

Table S11. Calculated V/[E] values for MiDAC activity toward mono-, penta-, and asymmetrically acetylated nucleosomes. Average V/[E] ± SEM values. Values marked with asterisks are re-printed from prior reports.[21,22]

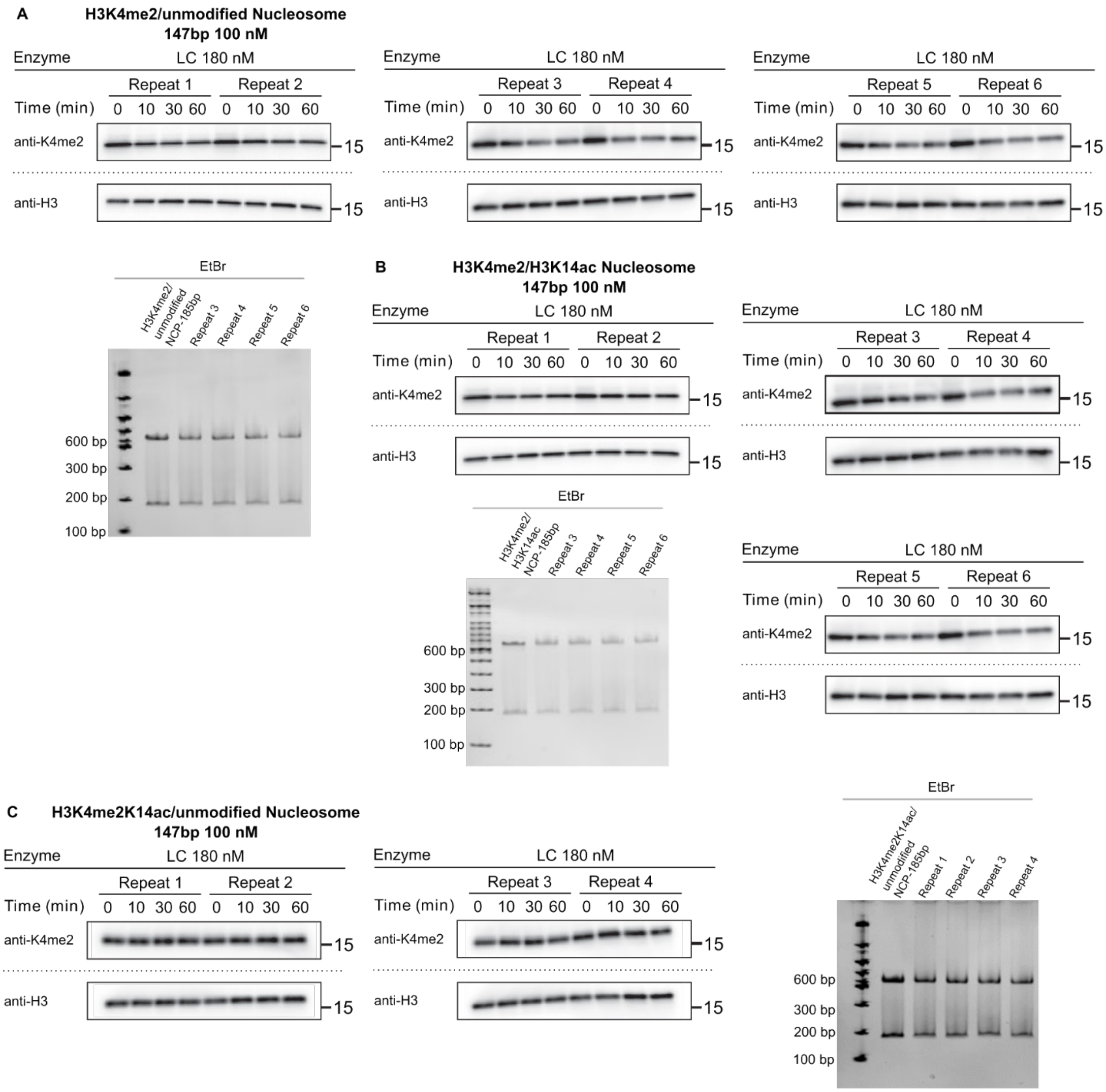

Figure S37. Western blot measurement of LC activity toward nucleosomes with asymmetric methylation and acetylation. (**A**) Western blots (top; anti-H3K4me2; n=6) and representative TBE native gel (left) of LC demethylation assays with 185 bp asymmetric unmodified H3 & H3K4me2 nucleosomes. (**B**) Western blots (top, right; anti-H3K4me2; n=6) and representative TBE native gel (center) of LC demethylation assays with 185 bp asymmetric unmodified H3K14ac & H3K4me2 nucleosomes. (**C**) Western blots (bottom; anti-H3K4me2; n=4) and representative TBE native gel (right) of LC demethylation assays with 185 bp asymmetric unmodified H3 & H3K4me2K14ac nucleosomes.

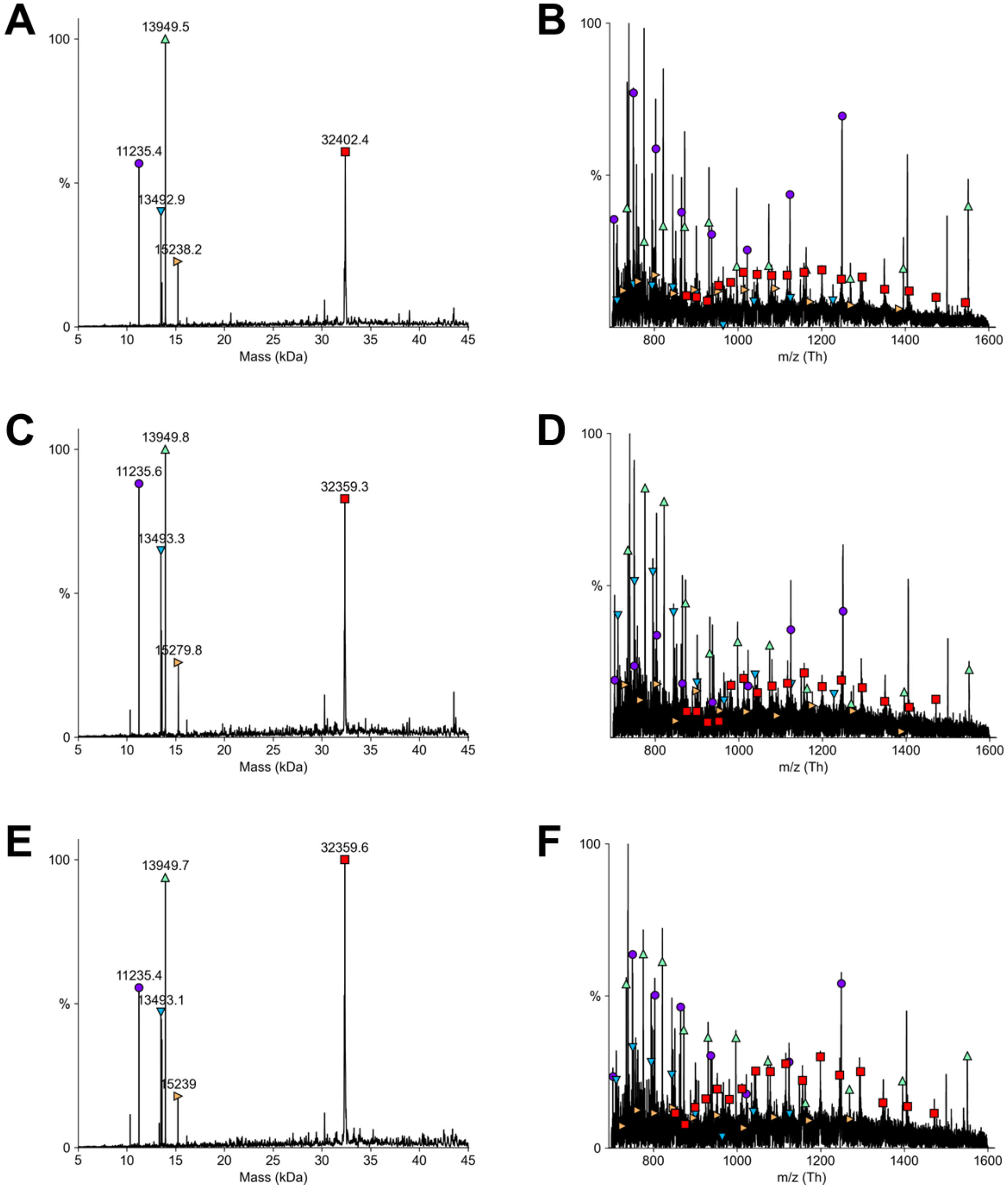

Figure S38. Mass spectrometric characterization of ubiquitinated H3 peptides and ubiquitinated nucleosome. Deconvoluted (**A**) and raw (**B**) mass spectra of asymmetric 185 bp H3K9me3/K18Ub(G76A)/K23Ub(G76A) & unmodified H3 nucleosome: H4 (purple circle) calculated mass 11236.15 Da, found: 11235.4 Da; H2B (teal downward pointed triangle) calculated mass 13493.68 Da, found: 13493.1 Da; H2A (green upward pointed triangle) calculated mass 13950.2 Da, found: 13949.7 Da; H3 (orange rightward pointed triangle) calculated mass 15238.61 Da, found: 15239.0 Da; H3K9me3/K18Ub(G76A)/K23Ub(G76A) (red square) calculated mass 32402.18 Da, found: 32402.4 Da. Deconvoluted (**C**) and raw (**D**) mass spectra of asymmetric 185 bp H3K18Ub(G76A)/K23Ub(G76A) &

H3K9me3 nucleosome: H4 (purple circle) calculated mass 11236.15 Da, found: 11235.4 Da; H2B (teal downward pointed triangle) calculated mass 13493.68 Da, found: 13493.3 Da; H2A (green upward pointed triangle) calculated mass 13950.2 Da, found: 13949.8 Da; H3K9me3 (orange rightward pointed triangle) calculated mass 15279.68 Da, found: 15279.8 Da; H3K18Ub(G76A)/K23Ub(G76A) (red square) calculated mass 32360.51 Da, found: 32359.3 Da. Deconvoluted (**E**) and raw (**F**) mass spectra of asymmetric 185 bp H3K18Ub(G76A)/K23Ub(G76A) & unmodified H3 nucleosome: H4 (purple circle) calculated mass 11236.15 Da, found: 11235.4 Da; H2B (teal downward pointed triangle) calculated mass 13493.68 Da, found: 13493.1 Da; H2A (green upward pointed triangle) calculated mass 13950.2 Da, found: 13949.7 Da; H3 (orange rightward pointed triangle) calculated mass 15238.61 Da, found: 15239.0 Da; H3K18Ub(G76A)/K23Ub(G76A) (red square) calculated mass 32360.51 Da, found: 32359.6 Da. Raw spectra deconvoluted with UniDec.[19]

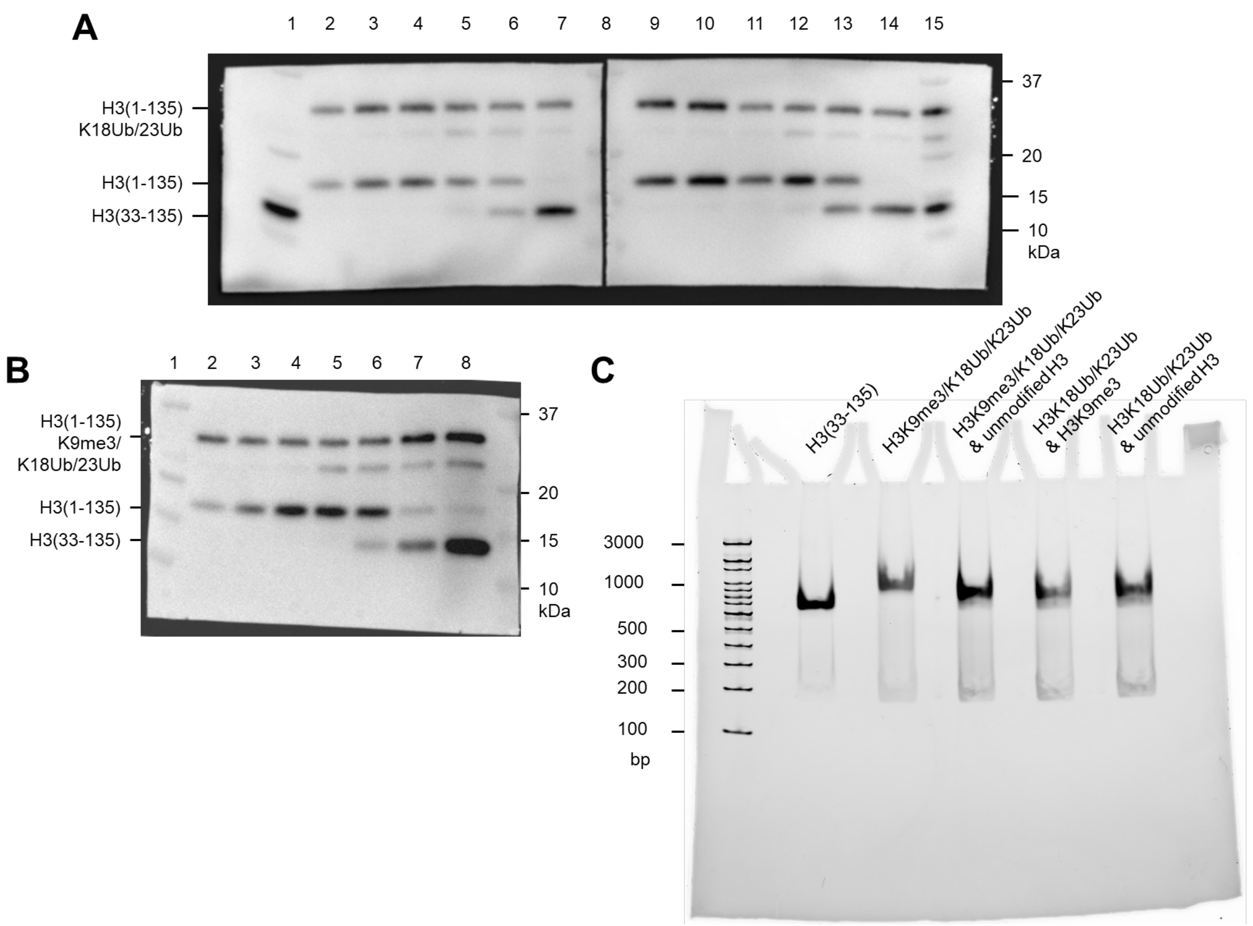

Figure S39. Characterization of asymmetric unmodified/ubiquitinated nucleosome. (**A**) Anti-H3 blot of tailless nucleosome starting material, intermediate single tail ligation product, asymmetrically modified products, and intermediate fractions from weak anion exchange purification.
Lanes: 1 – tailless starting material & ladder; 2 –final asymmetric H3K18Ub/K23Ub and unmodified H3 nucleosomes; 3-6 – impure weak anion exchange fractions in elution order ; 7 – intermediate single H3 tail product; 8 – ladder; 9 –final asymmetric H3K18Ub/K23Ub and H3K9me3 nucleosomes; 10-14 – impure weak anion exchange fractions in elution order 14 – intermediate single H3 tail product; 15 – ladder. (**B**) Anti-H3 blot of tailless nucleosome starting material, intermediate single tail ligation product, asymmetrically modified products, and intermediate fractions from weak anion exchange purification.
Lanes: 1 –ladder; 2 –final asymmetric H3K9me3/K18Ub/K23Ub and unmodified H3 nucleosomes; 3-8 – impure weak anion exchange fractions in elution order. (**C**) TBE native gel of 185 bp starting material symmetric ubiquitinated products of the cW11 sortase ligation, and asymmetric ubiquitinated products of the cW11 sortase ligation.

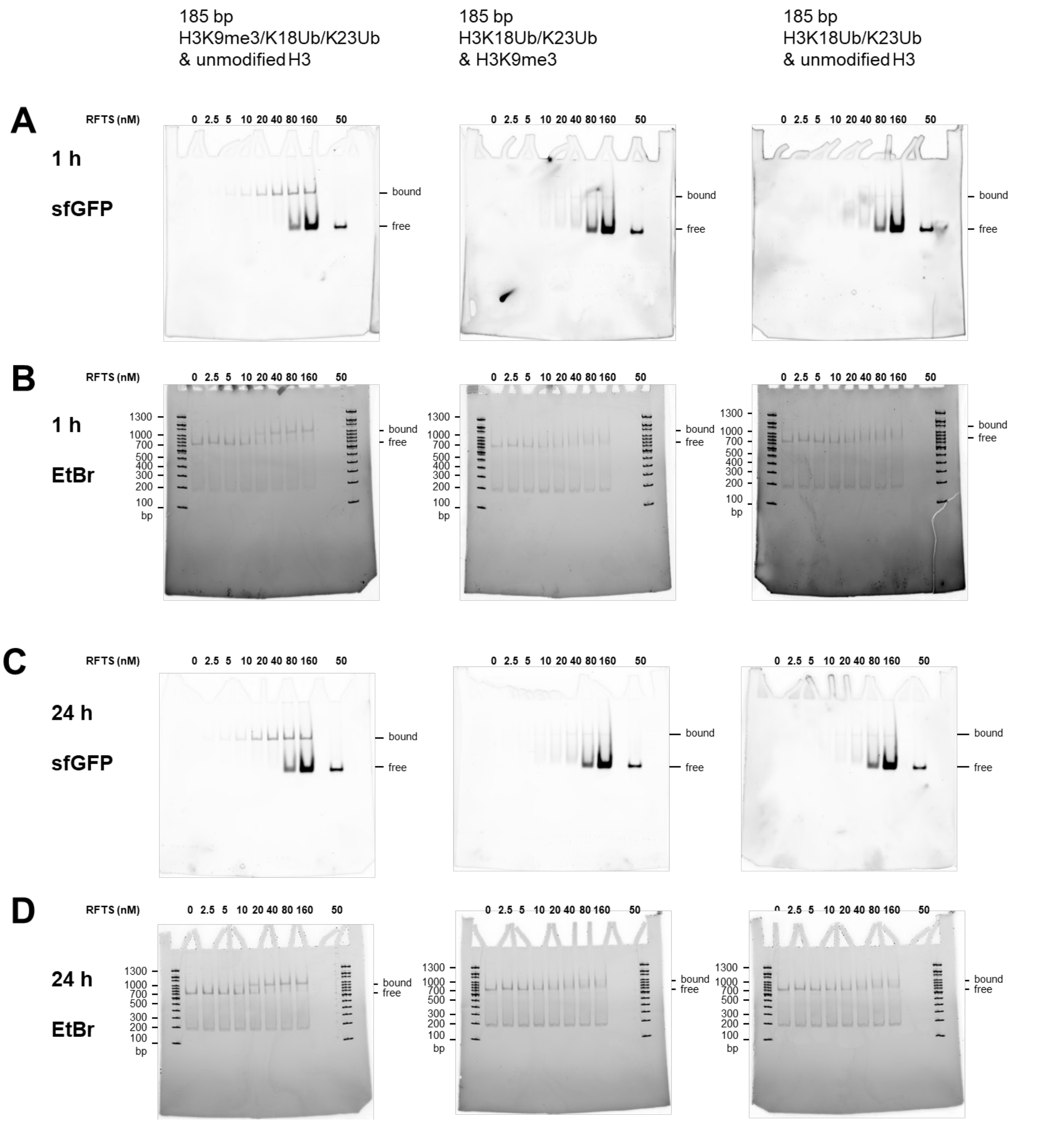

Figure S40. Electrophoretic mobility shift assay titrating asymmetric nucleosomes with sfGFP-RFTS fusion. (**A**) Fluorescence visualization of sfGFP-RFTS fusion (titrant) binding to 185 bp nucleosomes with asymmetric H3K9me3/K18Ub/K23Ub and unmodified H3 (left), asymmetric K18Ub/K23Ub and H3K9me3 (middle), or asymmetric K18Ub/K23Ub and unmodified H3 (right) after 1 hr at 4 °C. (**B**) Ethidium bromide (EtBr) visualization of sfGFP-RFTS fusion (titrant) binding to 185 bp nucleosomes with asymmetric H3K9me3/K18Ub/K23Ub and unmodified H3 (left), asymmetric K18Ub/K23Ub and H3K9me3 (middle), or asymmetric K18Ub/K23Ub and unmodified H3 (right) after 1 hr at 4 °C. (**A**) Fluorescence visualization of sfGFP-RFTS fusion (titrant) binding to 185 bp nucleosomes with asymmetric H3K9me3/K18Ub/K23Ub and unmodified H3 (left), asymmetric K18Ub/K23Ub and H3K9me3 (middle), or asymmetric K18Ub/K23Ub and unmodified H3 (right) after 24 hr at 4 °C. (**B**) Ethidium bromide (EtBr) visualization of sfGFP-RFTS fusion (titrant) binding to 185 bp nucleosomes with asymmetric H3K9me3/K18Ub/K23Ub and unmodified H3 (left), asymmetric K18Ub/K23Ub and H3K9me3 (middle), or asymmetric K18Ub/K23Ub and unmodified H3 (right) after 24 hr at 4 °C.

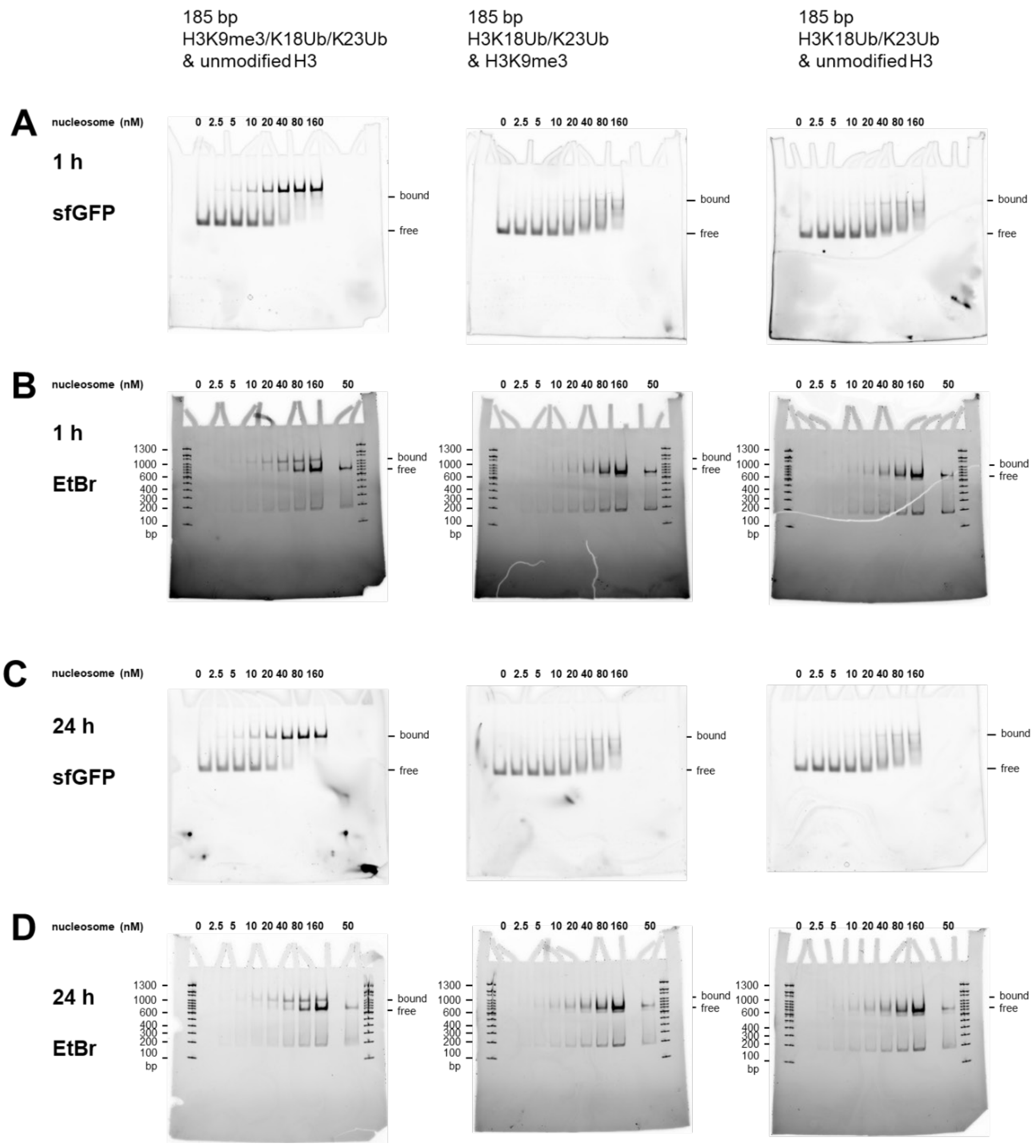

Figure S41. Electrophoretic mobility shift assay titrating sfGFP-RFTS fusion with asymmetric nucleosomes. (**A**) Fluorescence visualization of sfGFP-RFTS fusion binding to 185 bp nucleosomes (titrant) with asymmetric H3K9me3/K18Ub/K23Ub and unmodified H3 (left), asymmetric K18Ub/K23Ub and H3K9me3 (middle), or asymmetric K18Ub/K23Ub and unmodified H3 (right) after 1 hr at 4 °C. (**B**) Ethidium bromide (EtBr) visualization of sfGFP-RFTS fusion binding to 185 bp nucleosomes (titrant) with asymmetric H3K9me3/K18Ub/K23Ub and unmodified H3 (left), asymmetric K18Ub/K23Ub and H3K9me3 (middle), or asymmetric K18Ub/K23Ub and unmodified H3 (right) after 1 hr at 4 °C. (**A**) Fluorescence visualization of sfGFP-RFTS fusion binding to 185 bp nucleosomes (titrant) with asymmetric H3K9me3/K18Ub/K23Ub and unmodified H3 (left), asymmetric K18Ub/K23Ub and H3K9me3 (middle), or asymmetric K18Ub/K23Ub and unmodified H3 (right) after 24 hr at 4 °C. (**B**) Ethidium bromide (EtBr) visualization of sfGFP-RFTS fusion binding to 185 bp nucleosomes (titrant) with asymmetric H3K9me3/K18Ub/K23Ub and unmodified H3 (left), asymmetric K18Ub/K23Ub and H3K9me3 (middle), or asymmetric K18Ub/K23Ub and unmodified H3 (right) after 24 hr at 4 °C.

References:

1. Piotukh, K. *et al.* Directed Evolution of Sortase A Mutants with Altered Substrate Selectivity Profiles. *J Am Chem Soc* **133**, 17536–17539 (2011).
2. Chen, I., Dorr, B. M. & Liu, D. R. A general strategy for the evolution of bond-forming enzymes using yeast display. *Proc Natl Acad Sci U S A* **108**, 11399–11404 (2011).
3. Zhulenkovs, D., Jaudzems, K., Zajakina, A. & Leonchiks, A. Enzymatic activity of circular sortase A under denaturing conditions: An advanced tool for protein ligation. *Biochem Eng J* **82**, 200–209 (2014).
4. Musil, M. *et al.* FireProt: web server for automated design of thermostable proteins. *Nucleic Acids Res* **45**, W393–W399 (2017).
5. Shah, N. H., Dann, G. P., Vila-Perelló, M., Liu, Z. & Muir, T. W. Ultrafast protein splicing is common among cyanobacterial split inteins: Implications for protein engineering. *J Am Chem Soc* **134**, 11338–11341 (2012).
6. Abeywardana, M. Y. *et al.* Multifaceted regulation of Sirtuin 2 (Sirt2) Deacetylase Activity. *Journal of Biological Chemistry* 107722 (2024) doi:10.1016/j.jbc.2024.107722.
7. Wang, Z. *et al.* Structural Basis of Sirtuin 6-Catalyzed Nucleosome Deacetylation. *J Am Chem Soc* **145**, 6811–6822 (2023).
8. Wu, M. *et al.* Lysine-14 acetylation of histone H3 in chromatin confers resistance to the deacetylase and demethylase activities of an epigenetic silencing complex. *eLife* **7**, 1–19 (2018).
9. Kalin, J. H. *et al.* Targeting the CoREST complex with dual histone deacetylase and demethylase inhibitors. *Nat Commun* **9**, (2018).
10. Song, Y. *et al.* Mechanism of Crosstalk between the LSD1 Demethylase and HDAC1 Deacetylase in the CoREST Complex. *Cell Rep* **30**, 2699-2711.e8 (2020).
11. Turnbull, R. E. *et al.* The MiDAC histone deacetylase complex is essential for embryonic development and has a unique multivalent structure. *Nat Commun* **11**, 1–15 (2020).
12. Huang, Y. C. *et al.* Synthesis of l- and d-Ubiquitin by One-Pot Ligation and Metal-Free Desulfurization. *Chemistry – A European Journal* **22**, 7623–7628 (2016).
13. Worden, E. J., Zhang, X. & Wolberger, C. Structural basis for COMPASS recognition of an H2B-ubiquitinated nucleosome. *eLife* **9**, (2020).
14. Li, X. *et al.* Electron counting and beam-induced motion correction enable near-atomic-resolution single-particle cryo-EM. *Nat Methods* **10**, 584–590 (2013).
15. Zheng, S. Q. *et al.* MotionCor2: anisotropic correction of beam-induced motion for improved cryo-electron microscopy. *Nat Methods* **14**, 331–332 (2017).
16. Punjani, A., Rubinstein, J. L., Fleet, D. J. & Brubaker, M. A. cryoSPARC: algorithms for rapid unsupervised cryo-EM structure determination. *Nat Methods* **14**, 290–296 (2017).
17. Davey, C. A., Sargent, D. F., Luger, K., Maeder, A. W. & Richmond, T. J. Solvent Mediated Interactions in the Structure of the Nucleosome Core Particle at 1.9 Å Resolution. *J Mol Biol* **319**, 1097–1113 (2002).
18. Pettersen, E. F. *et al.* UCSF Chimera—A visualization system for exploratory research and analysis. *J Comput Chem* **25**, 1605–1612 (2004).

19. T. Marty, M. *et al.* Bayesian Deconvolution of Mass and Ion Mobility Spectra: From Binary Interactions to Polydisperse Ensembles. *Anal Chem* **87**, 4370–4376 (2015).
20. Abeywardana, M. Y. *et al.* Multifaceted regulation of Sirtuin 2 (Sirt2) Deacetylase Activity. *Journal of Biological Chemistry* **300**, 107722 (2024).
21. Wang, Z. A. *et al.* Diverse nucleosome site-selectivity among histone deacetylase complexes. *eLife* **9**, e57663 (2020).
22. Wang, Z. A. *et al.* Histone H2B Deacylation Selectivity: Exploring Chromatin's Dark Matter with an Engineered Sortase. *J Am Chem Soc* **144**, 3360–3364 (2022).
